# Global Emergency Medicine: A Scoping Review of the Literature From 2024

**DOI:** 10.1111/acem.70208

**Published:** 2025-12-23

**Authors:** J. Austin Lee, Nana Serwaa A. Quao, Amanda Collier, Chris A. Rees, Morgan C. Broccoli, Nanaba A. Dawson-Amoah, Vinay N. Kampalath, Joseph Ciano, Ashley Jacobson, Jennifer Jones, Joseph Leanza, Branden Skarpiak, Jonathan M. Strong, Braden J. Hexom, Global Emergency Medicine Literature Review (GEMLR) Group

**Affiliations:** 1 Department of Emergency Medicine, Indiana University School of Medicine, Bloomington, Indiana, USA; 2 Department of Emergency Medicine, Korle Bu Teaching Hospital, Accra, Ghana; 3 Department of Emergency Medicine, University of Ottawa, Ottawa, Ontario, Canada; 4 Department of Emergency Medicine, Queen’s University, Kingston, Ontario, Canada; 5 Division of Pediatric Emergency Medicine, Emory University School of Medicine and Children’s Healthcare of Atlanta, Atlanta, Georgia, USA; 6 Department of Emergency Medicine, Brigham and Women’s Hospital, Boston, Massachusetts, USA; 7 Department of Emergency Medicine, Greater Accra Regional Hospital, Accra, Ghana; 8 Department of Pediatrics, University of Pennsylvania School of Medicine, Children’s Hospital of Philadelphia, Philadelphia, Pennsylvania, USA; 9 Department of Emergency Medicine, Hospital of the University of Pennsylvania, Philadelphia, Pennsylvania, USA; 10 Department of Emergency Medicine, Yale University School of Medicine, New Haven, Connecticut, USA; 11 Department of Emergency Medicine, UMass Chan Medical School - Baystate Medical Center, Springfield, Massachusetts, USA; 12 Department of Emergency Medicine, Boston University School of Medicine and Boston Medical Center, Boston, Massachusetts, USA; 13 Department of Emergency Medicine, University of Colorado School of Medicine, Aurora, Colorado, USA; 14 Department of Emergency Medicine, Brigham and Women’s Hospital; Harvard Medical School, Boston, Massachusetts, USA; 15 Department of Emergency Medicine, Rush University Medical Center, Chicago, Illinois, USA

## Abstract

**Objective:**

The Global Emergency Medicine Literature Review (GEMLR) identifies impactful research in global emergency care. This 20th annual edition reviews GEM literature published in 2024 and highlights the growth of GEMLR over the years.

**Methods:**

We conducted a scoping review of 2024 GEM articles through structured PubMed and gray literature searches. Reviewers and editors from 10 countries screened abstracts using predefined criteria for three domains: disaster and humanitarian response (DHR), emergency care in limited-resource settings (ECLRS), and emergency medicine development (EMD). Duplicates and articles lacking authorship equity or ethical oversight were excluded. Remaining publications were scored using tailored rubrics for original research (OR), review articles (RE), and gray literature (GRAY). The top 5% in each category were selected for critical appraisal. A retrospective summary of 20 years of GEMLR reviews was also completed.

**Results:**

The search identified 46,714 PubMed and 12,575 gray literature articles. A total of 473 met inclusion criteria and were scored; 33 were selected as the top 5%, a decrease from 55 in 2023. Although the search string was unchanged, 2024 yielded ~10,000 fewer articles. Common themes included trauma, pediatrics, and clinical/triage protocols, with a new focus on mental health among clinicians and disaster victims. Over 20 years, 230 individuals have contributed 810 service-years to GEMLR. Nearly 75% of members were from the USA, while 32 (13.9%) were from LMICs and 27 (11.7%) from non-USA high-income countries. In total, 398,904 articles have been screened, 8476 scored, and 517 top articles narratively reviewed since 2005.

**Conclusions:**

Over two decades, GEMLR has evolved into a large-scale, multinational, methodologically rigorous initiative, highlighting more than 500 high-impact GEM publications. In 2024, despite fewer articles screened, 33 top studies were identified across key domains. GEMLR emphasizes equitable LMIC representation, rigorous quality standards, and authorship equity, aiming to help shape the future of emergency care research.

## 1 | Introduction

The Global Emergency Medicine Literature Review (GEMLR), established in 2005, is now finishing its twentieth year with the continued aim to advance the practice of emergency medicine (EM) worldwide by enhancing access to high-impact research conducted in global settings [1–20]. GEMLR identifies, evaluates, and synthesizes relevant literature to provide a curated resource for clinicians, educators, researchers, and policymakers engaged in global EM (GEM) practice and research. GEMLR operates within a framework that emphasizes clinical care and system development in resource-limited and humanitarian settings, where EM is often emerging or underdeveloped. Each year, a diverse panel of reviewers sorts and critiques literature identified through a broad search, seeking to showcase the evolving scholarship of global EM practice and identify high-quality research produced in developing emergency care systems worldwide.

The GEMLR team’s approach to surveying GEM literature has evolved over time. Across the years, the teams have brought improvements including the integration of scoring rubrics, modifications to the search string, revisions to the gray literature search, tailoring of scoring criteria, and efforts to ensure equity in authorship. A common thread has remained consistent throughout two decades of GEMLR publications: the aim of systematically identifying and highlighting the most useful publications in GEM literature. Since 2009, the GEMLR scoping review has categorized and summarized literature under three primary domains: disaster and humanitarian response (DHR), emergency care in limited-resource settings (ECLRS), and emergency medicine development (EMD). The GEMLR Group, since 2017, has also conducted a targeted systematic review each year that explores focused topics in emergency medicine where previous work has highlighted a lack of consensus [21–27].

Over the past 20 years, the GEMLR project has provided hundreds of individuals from all over the world experience in reviewing and assessing medical evidence, has provided an opportunity for those interested in academic GEM–regardless of country of origin–a pathway to authorship of peer-reviewed literature, and has supported the professional development of academic GEM physicians. As part of the twentieth anniversary milestone, the GEMLR team undertook a retrospective review of its membership and the body of articles assessed to date. This effort sought to illustrate the project’s membership growth, the trends in the number of GEM publications over time, and guide the evolution of GEMLR into its third decade.

## 2 | Methods

After several updates to the search and scoring strategy in the last few annual scoping reviews, no substantive changes were made for the 2024 research year. Each year, the editorial team updates a procedure manual that guides the review’s policies and processes (Data S1). Included in the screening process is a requirement for local co-authorship to support equity in GEM research, and all original research (OR) articles must include a statement of approval from a local ethical review board. All reporting is in accordance with Preferred Reporting Items for Systematic reviews and Meta-Analyses extension for Scoping Reviews (PRISMA-ScR) checklist (Data S2) [28].

### 2.1 | GEMLR Recruitment and Personnel

The GEMLR Group is intentional in working to actively recruit researchers and practitioners from around the world and in particular from low- and middle income countries (LMICs). This year’s review included a number of incoming members representing new institutions and countries. The returning membership included individuals with formal GEM training and broad experience in GEM research around the world. The GEMLR Group actively recruits through professional networks, GEM communities, and online postings. Applicants are selected by the editorial team to fill open reviewer and alternate reviewer slots opened through promotion, attrition, and term limits.

This year’s GEMLR team included 54 representatives from 10 countries (Cameroon, Canada, Germany, Ghana, Kenya, Nigeria, Tanzania, Uganda, Zambia, and the United States). The team included a four-member editorial team (editor in chief, managing editor, assistant managing editor, technical editor), 10 editors (two senior editors, three editors, and five assistant editors), 15 returning senior reviewers, 22 reviewers, and 3 alternate reviewers (Appendix A). There were five new advisors, in addition to seven returning advisors. Each GEMLR review team is composed of an editor supervising several reviewers, with each team containing two to five reviewers. This year’s GEMLR Group included 12 members based in seven LMICs, an increase from 10 members from five LMICs last year. All GEMLR Group members are volunteers and unfunded for their participation.

### 2.2 | Article Screening Procedures

All reviewers, both new and returning, completed a supervised practice screening and scoring exercise with their group editor to ensure familiarity with procedures and to reinforce consistency across the process. This also established a foundation for group communication and allowed editors to identify reviewers who may benefit from additional assistance.

A PubMed search covering all of 2024 was conducted in two phases—an initial search of the first 8 months, followed by a second tranche with publications from the last 4 months—to capture all GEM articles published in English, Spanish, and French. The PubMed search was complemented by a targeted search of high-yield journals: *African Journal of Emergency Medicine, Bulletin of the World Health Organization, The Lancet, and Prehospital and Disaster Medicine*. The search strategy for this year’s review can be found in Data S3.

After the PubMed search, article abstracts were divided among the reviewers. As outlined in the procedure manual, reviewers identified articles meeting the DHR, ECLRS, and EMD inclusion criteria, and articles screened-in by reviewers were again screened by their assigned editor. DHR addresses research on disaster victims and populations affected by war or humanitarian crises, ECLRS focuses on acute clinical management in low-resource settings, and EMD takes a broader view of advancing emergency medicine and care systems. Compiled screened-in lists were reviewed a third time by the managing editorial team. For articles not clearly associated with a specific country or setting, or when standard inclusion criteria could not be clearly applied, inclusion was determined at the discretion of the editorial team. The gray literature screen includes a manual screening of 11 organizations’ websites (Data S1). An editor and two reviewers collated and screened these sites to identify independent research published by these groups of particular interest to GEM practitioners.

### 2.3 | Article Scoring and Inclusion Procedures

Screened articles meeting inclusion criteria were divided among the reviewers for full text review. Full text of all articles was uploaded to a shared drive for manual review. Individual full text articles were reviewed for ethical oversight and authorship equity requirements as outlined in the procedure manual. If ethics and authorship criteria were met, reviewers and editors assign attributes to each article including review articles (RE) versus original research (OR), as well as DHR, ECLRS, or EMD. OR articles were further designated as qualitative, quantitative, or mixed methods. All articles were independently evaluated by two reviewers, blinded to one another, using standardized 20-point scoring rubrics (specific to OR and RE articles) that assessed study design, importance, and impact, with additional criteria for ethics (OR only) and clarity (RE only). Gray literature articles were also scored using a 12-point scoring rubric (Data S4). A standardized scoring excel file is used to uniformly compile all reviewers’ scores.

After scoring, a mean score and the two reviewer score-differences for each article are calculated. From this, the standard deviation for all mean scores is derived. Articles with a score-difference between the two reviewers of ≥ 2 standard deviations for OR articles and ≥ 1 standard deviation for RE articles were reviewed and potentially rescored by an editor if such a score would reach the threshold for inclusion as a top scoring article. After any rescoring, the top 5% of articles (rounded down) in each of the categories (OR, RE, DHR, ECLRS, EMD, and Gray) were then included as the top articles for full written review. Most reviewers were assigned one article to summarize and provide an accompanying commentary, using a common format to highlight each top article’s strengths, limitations, and relevance to global emergency medicine.

### 2.4 | Retrospective GEMLR Methods

This year also included a retrospective summary of all previous data from all 20 GEMLR reviews to date, including data from the current review. Each of the previously published GEMLR scoping review articles was hand reviewed and data regarding reviewers, authors, editors including years of participation and country affiliations were abstracted. Categories (EMD, ECLRS, and DHR) were not used in the first 4 years and were retrospectively categorized by an editor. Descriptive statistics were used to analyze participants and the historical number of articles screened and reviewed by the GEMLR Group. Four individuals had affiliations over time in both the USA and non-USA high-income countries, and all were tallied as non-USA high-income participants. Authorship data regarding the targeted systematic review work done by the GEMLR Group was totaled, but in almost all cases, the co-authors of these works were also serving concurrently in roles on the main review.

## 3 | Results

### 3.1 | 2024 Results

**FIGURE 1 |:**
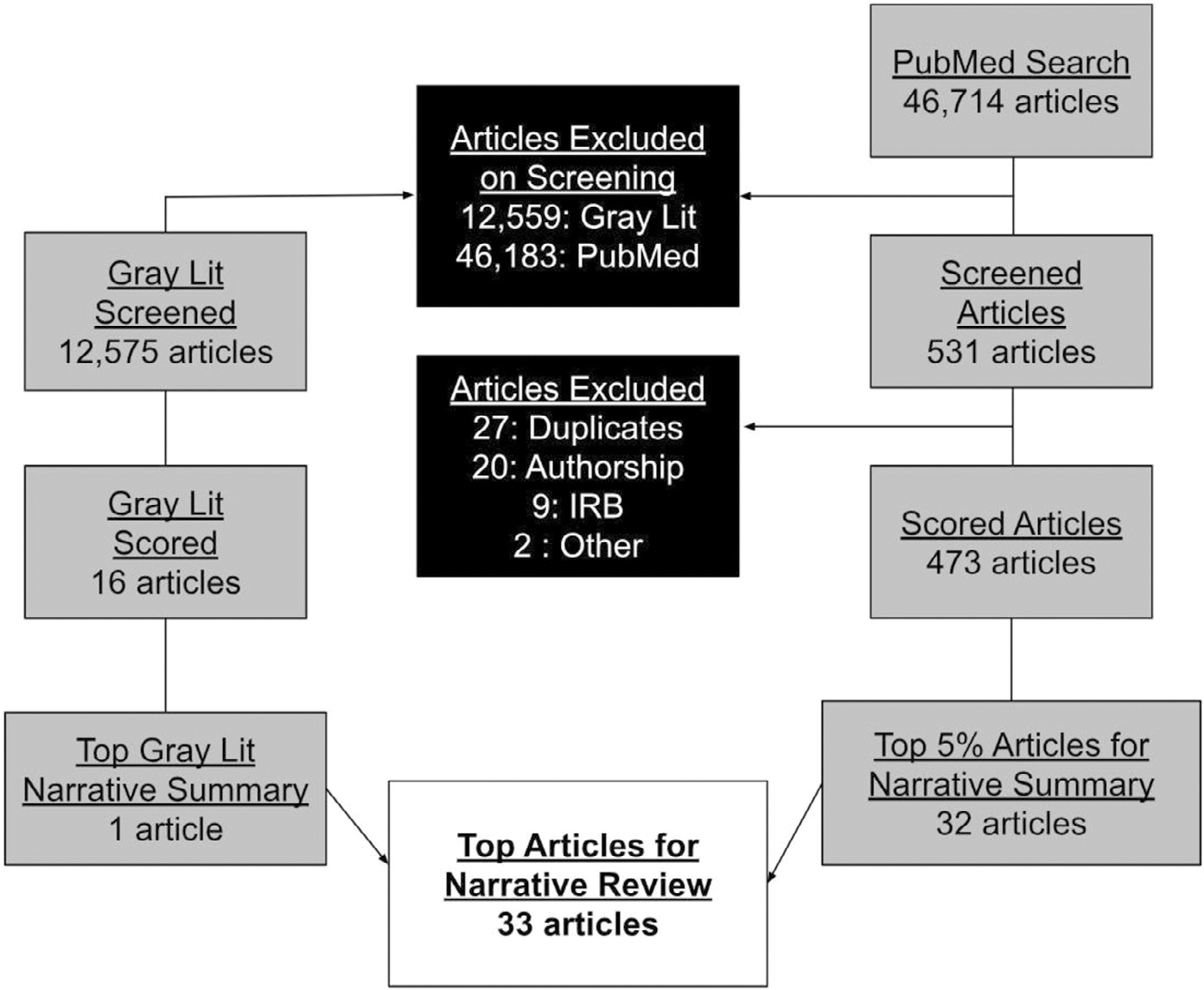
Flow diagram of articles reviewed.

For 2024, 46,714 articles were screened in the main search and another 12,575 articles were screened from the 11 gray literature sources (a total of 59,289 articles screened; Figure 1). A total of 46,183 main search and 12,559 articles were excluded on screening; an additional 27 duplicates were removed, one article was excluded as it was only published as an abstract and did not have a full text associated with it, and one article was excluded for being published in 2022. Of the remaining 502 articles, 20 were excluded for lacking an author from the country of study and nine were OR articles that were excluded for lack of review from an ethical board. A total of 473 journal articles and 16 gray literature articles were fully scored by two reviewers (Table 1). A total of 33 articles were identified in the top 5% of each category (Table 2), including 5 (15%) DHR, 24 (73%) ECLRS (including the single gray literature article), and 4 (12%) EMD. There were 22 (67%) OR and 11 (33%) RE articles; of the OR articles, none were qualitative, 21 (95%) were quantitative, and one (5%) utilized mixed methods.

The top scoring articles addressed several themes in emergency medicine. As in past years, common subject matter included: trauma (eight articles), pediatrics (five), and both clinical and triage protocols (six). This year a new theme was noted, with several top articles on the mental health of clinicians and disaster victims (three articles). Other themes included prehospital care (two articles) and one article each in toxicology, snakebites, research methods, sepsis, COVID-19, stroke, cardiac care, point-of-care ultrasound, and palliative care in the ED. Topics from last year that were not included in the top scoring articles this year were cardiopulmonary resuscitation (CPR), malaria, maternal and neonatal care, and education/training.

### 3.2 | Two Decades in Review

Looking back over the past 20 years of reviews, the GEMLR teams have reported screening a total of 398,904 main articles (Table 3). From 2022 to 2024, there were 31,365 gray literature articles (there were 6 years without a gray literature search and 11 years where the gray literature search did not report the number of articles screened). In total, GEMLR teams have scored 8476 articles over 20 years, with 517 articles given a full narrative review as top-quality articles. Over the past two decades, this equates to 2.0% of all screened articles being scored, and 6.3% of all scored articles being included as top articles for full narrative review. Of the 517 articles given a full narrative review, 109 (21.1%) have been from DHR, 333 (64.4%) from ECLRS, and 75 (14.5%) from EMD. Early years had a higher concentration of DHR articles, and over time there has been an increasing proportion of ECLRS.

Two-hundred and thirty different people have served in GEMLR team member roles for at least 1 year; 32 (13.9%) have been from LMICs, 27 (11.7%) from non-USA high-income countries, and 169 (74.3%) from the USA (Data S5). The mean duration of GEMLR participation by any one individual (in reviewer, author and advisory roles) is 3.3 years (range: 1 to 17 years). Eleven individuals served the GEMLR Group for 10 or more total years.

Across the past decades, the main GEMLR scoping reviews have had between 6 and 14 listed authors. There have been 11 different first authors and 64 total listed authors across the 20 iterations of the review. Of these 64 listed authors, 6 (9.4%) were from LMIC settings, four of whom were from Ghana, one from India, and one from Brazil. In the past decade, there has been at least one and as many as four LMIC editors/authors per year. The mean number of years in an author-level position has been 3.1 years (with a range of 1–14 years). Thirty-seven individuals have served as GEMLR advisors after completing terms as reviewers or in author-level roles, serving a mean of 2.1 years (range 1–8 years). There have been an aggregate 750 service-years volunteered to the GEMLR Group main review, and another 60 service-years among the targeted reviews.

Among the total 32 GEMLR Group members from LMICs, there have been a mean of four per year (range: 0–12 per year), serving in any GEMLR role for a mean duration of 2.5 years (range: 1–9 years). In the first 15 years of the GEMLR review, there were never more than six participants from LMICs. In the past few years, LMIC representation has increased, with six LMIC members in 2021 and 2022, ten members in 2023, and twelve members in 2024. To date, there have not been any first authors from an LMIC.

There have also been a total of 26 members (all receiving authorship) for the eight targeted systematic reviews, all but two of whom were previously reviewers and/or editors on the main scoping review. Neither participation as a targeted review author nor the number of articles screened as part of the targeted systematic reviews were included in tallies above (except for two targeted review members whose only-one-year participation was counted in the total count of 230 GEMLR Group membership).

## 4 | Discussion

Across 20 years, GEMLR has grown, from six people scoring 48 articles to select the top 10 works from the first iteration in 2005 to 54 people screening nearly 60,000 articles annually and fully scoring several hundreds of these with a rigorous reproducible methodology to identify and assess the top tier of GEM articles. Hundreds of individuals have been a part of this two-decade-long effort to volunteer over 800 service-years of work to identify and highlight the most useful top-quality research from emergency medical care in resource-limited settings. In addition to the 20 GEMLR scoping reviews, the group has also produced a number of targeted systematic reviews.

There has been an evolving discussion of equity in authorship across global health, and GEMLR has sought to contribute to improving equity and representation in global health literature [62–64]. While global researchers have identified difficulty navigating local and global academic pressures while facing concerns about the perceived value of research, mentorship is also a recognized challenge in research partnerships [65, 66]. While there is a critical need to confront the colonial antecedents of what is now recognized as global health, the current paradigm of researchers in high-income countries working to support LMIC researchers is at risk of concentrating power and expertise among a select few [67, 68]. Women comprise the majority of the global health workforce in LMICs, and yet there is not a proportional representation in research and leadership roles [69]. There is a need to acknowledge a tension between wanting to create local emergency medicine researchers around the globe and understanding who may receive protected time, funding, or is otherwise incentivized to participate in research or academic efforts to highlight global EM research. In recognition of these overdue paradigm shifts, GEMLR leadership has been intentional over the past decade in recruiting and developing a diverse group of reviewers and editors; these efforts have clearly borne fruit these past 5 years.

The 2024 GEMLR scoping review did not make any substantive changes to the search string, and yet there were 11,577 fewer articles to screen from the year prior (in 2023, GEMLR screened 58,291 articles, of which 825 were scored, and the 55 top articles were given a full write up) [19]. The cause for the significant decrease in results is not clear, and future iterations of GEMLR will help to delineate if this is a temporary aberration or a longer-term trend. The total number of top 5% scoring articles in 2022 was similar to this year, at 37 [18]. Several possible causes related to these decreases could be considered. Perhaps large swathes of global research were essentially halted during COVID-19, and the current 2024 review captures articles that most likely took place several years ago when downstream effects of COVID-19 related impacts are most felt. Both LMIC-based research as well as GEM research supported through high income country (HIC)-based academics may have waned during this time period; many institutions drastically reduced or completely suspended global travel support during that time. Future years may point towards onward growth or stagnation.

The overall percentages of top scoring articles between DHR, ECLRS and EMD were similar to recent years, and the split between OR and RE articles was nearly identical to 2023. The gray literature search included 1540 more than last year (in 2023 there were 11,035 articles screened and 37 gray literature articles scored, with one top scoring article). The majority of the increase in the gray literature articles came from one source, the United States Centers for Disease Control (which increased from 9451 articles screened in 2023 to 10,831 articles screened in 2024). In 2023, 33 articles were excluded for not meeting local authorship equity or IRB criteria, and there were 29 excluded for these reasons in 2024.

This scoping review highlights the highest-quality global EM research published in 2024 and identifies several notable thematic shifts. As expected, COVID-19 focused publications have declined. Mental health, particularly in disaster and humanitarian contexts, featured prominently among top articles, as did studies evaluating clinical and triage protocols, which are especially relevant for settings with nascent emergency care systems. Notably, no top scoring articles addressed emergency medicine education or training this year. The decline in top-scoring medical education content may partly reflect rising standards for what qualifies as a top article, as many medical education studies may not score highly given their typical methods and the impact of their findings. High-impact studies also focused on pediatric and trauma care, and a number of the top scoring articles were conducted in Iran. One particularly compelling article explores the role of emergency departments in providing palliative care. All 473 articles that met inclusion criteria and underwent full-text scoring are listed in Data S6; narrative summaries of the top 33 articles appear in Data S7.

### 4.1 | Disaster and Humanitarian Response (DHR)

Of the five top scoring DHR works, three were review articles. Cameron et al.’s systematic review and meta-analysis found high rates of mental ill-health among humanitarian aid workers across diverse settings [29]. Davidson et al.’s systematic review of gray literature found limited and inconsistent ethical guidance for research with refugees and asylum seekers, highlighting the need for improved guidance for those engaged in this important work [30]. Khedr et al. evaluated a multi-week multi-session psychological first aid intervention among post-earthquake refugee camps and shelters in Northern Syria and found that the intervention group experienced statistically significant improvements in resilience capacity, quality of life, and meaning of life measures [31]. Work by Tahernejad et al. evaluated the existing literature around the use of artificial intelligence in the development of triage systems, which could allow for more efficient care and improved triage of patients injured in disasters and emergencies [32]. In a piece published in BMC Pediatrics, Tesfay et al. evaluated children with severe acute malnutrition admitted following the outbreak of war in Tigray, and found early recovery was more likely in those with markers of mild disease such as taking oral antibiotics, those who did not need a blood transfusion or IV fluids, and the ability to tolerate oral (over nasogastric) feeding [33].

### 4.2 | Emergency Care in Limited Resource Settings (ECLRS)

There were 24 articles that met criteria for the ECLRS subsection, 16 works of original research (including the one top-scoring gray literature article) and 8 review articles. Adal et al. performed a systematic review with meta-analysis to assess mortality rates and identify factors contributing to death from traumatic chest injuries in sub-Saharan Africa; the authors found the pooled mortality rate was 9% with several clinical factors such as older age and poly-trauma to be significant predictors of mortality [34]. Ahmad et al. systematically reviewed the use of telemedicine in EDs across six heterogeneous RCTs, and they concluded that telemedicine has potential for particular benefit in triage and non-critical care support [35]. In a randomized controlled trial by Arora et al., among trauma patients in India requiring a chest tube, continuous wall suction was found to be superior to standard water seal alone in the duration of chest tube placement and length of hospitalization [36]. A modeling study by Bonnet et al. was the top scoring gray literature article. They found that the cost-effectiveness of COVID rapid diagnostic tests (RDTs) in LMICs varies by local COVID and influenza prevalence and resource availability, with RDTs generally recommended in poorer settings with high viral respiratory infection prevalence rates and in all cases for wealthier LMICs [37]. In a single-center comparative observational study of pediatric hematology-oncology patients with febrile neutropenia in Mexico, Colunga-Pedraza et al. found that a multidisciplinary “Golden Hour” intervention effectively increased timely antibiotic administration in an LMIC setting [38].

A stepped-wedge randomized controlled trial of over 2400 participants conducted in rural Ugandan hospitals in work by Conradi et al. demonstrated a statistical reduction in mortality of children 48 h from detection of hypoxemia when provided access to solar-powered oxygen concentrator systems [39]. In a randomized controlled trial by Daihimfer et al. involving 180 children aged 3–6 years in an Iranian emergency department, it was found that both music and acupressure significantly reduced venipuncture pain compared to no intervention, with music showing the greatest effect [40]. A systematic review with meta-analysis by Endeshaw et al. included 33 articles and over 6100 patients to identify predictive factors of mortality from abdominal trauma in sub-Saharan Africa, with noted increased mortality among patients with shock, blunt trauma mechanisms, admitted to the ICU, and those with postoperative complications [41]. Gyedu et al. used a quality improvement implementation approach to evaluate the utilization of a decision-support trauma intake form with clinical prompts, which were found to improve initial trauma care both in district and regional hospitals in Ghana [42]. In a Colombian ED, Jaramillo et al. performed a retrospective observational study using a physician-in-triage protocol for moderate complexity patients (triaged 3 on a 1–5 scale), which reduced patient time to disposition and length of stay [43].

In a meta-analysis by Karamian et al., the authors found a high rate of intracranial hemorrhage (9.4%) from mild traumatic brain injury mechanisms among patients on oral anticoagulation from both high and upper-middle income settings, with lower rates among those taking direct oral anticoagulants over those on vitamin K antagonist medications [44]. Kefyalew et al. evaluated a nurse-led analgesia protocol in an Ethiopian emergency department and found this reduced the time to pain medication administration and improved patient satisfaction as compared to another clinical site [45]. Legesse et al. performed a multi-institutional observational study in Ethiopia to validate the Raja Isteri Pengiran Anak Saleha Appendicitis (RIPASA) scoring system and found it to be a reasonable alternative for diagnosing acute appendicitis in resource-limited settings where imaging, particularly ultrasound, is not always accessible [46]. In a systematic review by Quake et al., the authors summarized the current variable development of prehospital trauma care systems in LMICs, highlighted common barriers to development and implementation, and presented successful case studies which can inform future efforts in LMIC settings [47]. In a prospective observational cohort in Morocco, Rahmani et al. evaluated capillary refill time assessed visually against a pulse oximeter and found it to be a cost-effective, noninvasive way to measure tissue perfusion; further, they found capillary refill time correlated with serum lactate levels in those patients with sepsis or septic shock [48].

In an Iranian cohort study, Rahnemayan et al. showed that the NIHSS-8, a shorter version of the original stroke scale, is a potentially reliable and efficient alternative to the NIHSS-11 for the diagnosis of stroke in emergency care settings [49]. Ramamoorthy et al. performed a prospective cohort study of Indian adult ocular trauma patients in the emergency department and found emergency physician performed POCUS showed high sensitivity and specificity in detecting relative afferent pupillary defects [50]. A scoping review by Ranjbar Hameghavandi et al. identified 82 articles summarizing challenges in traumatic spinal cord injury prevention and management in low- and middle-income countries, with noted challenges including gaps in injury prevention, lack of sufficient data and research, underdeveloped pre-hospital systems, inadequate in-hospital care, and post-hospital rehab and follow-u p [51]. In a large meta-analysis covering nearly 95,000 patients from 45 countries, Shakir et al. assessed the time to intervention after a TBI and found significant delays in care across prehospital and intrahospital settings, with disparities based on country-level income, region, and healthcare payment system [52]. In a retrospective descriptive study in Thailand, Sri-on et al. identified and characterized significant missed opportunities to assess the need for palliative care in the emergency department patients suffering with life-limiting illness; improved transitions to palliative care have the potential to improve overall quality of life, decrease pain, and decrease medical costs, particularly during end-of-life emergency care in LMIC settings [53].

Stephen et al. performed a prospective observational study of individuals presenting with snakebites to a tertiary hospital in eastern India and summarized factors associated with these bites and clinical outcomes, highlighting the need for programs to improve public knowledge of first aid measures to care for snakebites and the importance of seeking timely medical care for consideration of potentially life-saving antivenom [54]. A retrospective cross-sectional study in Thailand by Tanaanantarak et al. identified clinical predictors of intracranial hemorrhage on CT in pediatric head trauma, including injury mechanism and severity, reduced GCS, signs of skull fracture on exam, and vomiting ≥ 3 times [55]. In an integrative review by Wang et al., the authors evaluate how psychological first aid (PFA) has been shown to have a positive effect on anxiety and adaptive functioning following traumatic exposure, but significant variability in format and implementation complicates the development of best practices for PFA [56]. In a retrospective cohort of Chinese patients with ST-segment elevation myocardial infarction, Yang et al. analyzed emergency medical service utilization highlighting disparities in EMS use across different regions and the influence of socioeconomic factors, despite findings that EMS utilization significantly improved treatment timelines [57].

### 4.3 | Emergency Medicine Development (EMD)

There were four original research articles that met emergency medicine development criteria this year. Behnoush et al. conducted a double-blinded randomized controlled trial in Tehran, Iran, evaluating the use of intravenous lipid emulsion therapy among patients with acute tramadol poisoning and a GCS of less than 12. Researchers found those with lipid administration had significantly reduced seizure frequency, length of hospitalization, and higher GCS when compared to controls receiving treatment with saline alone [58]. Koko et al. performed a cross-sectional study regarding the use of the ABCDE approach while managing trauma patients in three of the major trauma centers in Khartoum, Sudan, showing only 37.9% adherence to the approach which suggests needed improvements in education and implementation of trauma management guidelines [59]. In the only top-scoring article utilizing mixed methods, Michalski et al. evaluated the status of the prehospital care systems in Senegal, finding 80% of regions had no such established systems, and existing first responders often lack basic emergency care and first aid knowledge and skills [60]. Nasr Isfahani et al. performed a triple-blinded, randomized controlled trial in an Iranian trauma center and assessed pain management modalities in isolated closed extremity fractures in adults. Findings showed improved pain control at 60 min in patients receiving IV ibuprofen-acetaminophen group as compared with IV morphine or IV ibuprofen alone [61].

## 5 | Conclusion

Over two decades, the GEMLR has evolved into a rigorous, large-scale scoping initiative, now involving over 50 team members annually and screening tens of thousands of articles to identify high-impact global EM research. Since 2005, GEMLR has had 230 members, who have screened nearly half of a million articles, and to date have helped to collate and highlight over 500 of the most high-impact articles in global emergency medicine research. The 2024 review has identified the top 33 articles in the DHR, ECLRS, and EMD domains. Despite a drop in the total articles screened compared to 2023, the proportional distribution of top articles across domains and methodological categories remained similar to prior. As the field of GEM evolves, GEMLR continues to emphasize methodological rigor, equitable authorship, and increased representation from LMIC collaborators. The GEMLR group continues to work to serve a role highlighting and shaping the future of emergency care research globally.

## Supplementary Material

Supp Mat 3

Supp Mat 2

Supp Mat 4

Supp Mat 6

Supp Mat 1

Supp Mat 5

Supp Mat 7

Supporting Information

Additional supporting information can be found online in the Supporting Information section. Data S1: acem70208-sup-0001-Supinfo1.pdf. Data S2: acem70208-sup-0002-PRISMA-ScR-Checklist.pdf. Data S3: acem70208-sup-0003-Supinfo2.pdf. Data S4: acem70208-sup-0004-Supinfo3.pdf. Data S5: acem70208-sup-0005-Supinfo4.pdf. Data S6: acem70208-sup-0006-Supinfo5.xlsx. Data S7: acem70208-sup-0007-Supinfo6.pdf.

## Figures and Tables

**TABLE 1 |:** Summary of articles scored (excluding GRAY).

	Number (%)	Scores (out of 20)				
		Minimum	25th Percentile	Median	75th Percentile	Maximum

Total	473	7.5	12.5	14	15	19
Article category						
Disaster and humanitarian response (DHR)	74 (16)	7.5	12	13	14.5	18
Emergency care in resource-limited settings (ECLRS)	336 (71)	7.5	12.5	14	15.5	19
Emergency medicine development (EMD)	63 (13)	8	12.5	14	15	18.5
Type of research article						
Original research (OR) article	406 (86)	7.5	12.5	13.5	15	19
Review (RE) article	67 (14)	9.5	13.5	15	16.5	19
Type of original research article						
Quantitative study	337 (83)	7.5	12.5	14	15	19
Qualitative study	57 (14)	8	11.5	13.5	15	16.5
Mixed methods study	12 (3)	10.5	13.4	14.2	15	16.5

**TABLE 2 |:** Top-scoring GEMLR 2024 articles by category and alphabetical.

Category	First author	Article title	Journal	OR or RE	Summary [29]

Disaster and Humanitarian Response (DHR)	Cameron L [29]	The occurrence of and factors associated with mental ill-health among humanitarian aid workers: A systematic review and meta-analysis	PLoS One	RE	Humanitarian aid workers across a range of humanitarian settings face high rates of mental ill-health
	Davidson N [30]	Ethical considerations in research with people from refugee and asylum seeker backgrounds: A systematic review of national and international ethics guidelines	J Bioeth Inq	RE	This systematic review of gray literature reveals scarce and heterogenous ethical guidelines for conducting research in refugee and asylum-seeking populations. The authors advocate for the development of specific ethical guidelines for these vulnerable populations
	Khedr MA [31]	The feasibility of a psychological first aid intervention as a supportive tactic for feelings of psychological distress and mental health recovery outcomes among earthquake survivors in Northern Syria	Int J Nurs Pract	OR	The implementation of a psychological first aid intervention-which is grounded in psychological safety, cognitive reframing, mobilization of social support, and installation of hope-may have favorable outcomes in the event of public health emergencies, traumatic events, or even personal crises
	Tahernejad A [32]	Application of artificial intelligence in triage in emergencies and disasters: a systematic review	BMC Public Health	RE	Use of artificial intelligence in the development of triage and assessment systems may allow for more efficient care and improved resuscitation of patients injured in disasters and emergencies
	Tesfay W [33]	Stabilizing time and its predictors among 1–59 months old children managed for severe acute malnutrition during the humanitarian crisis in Tigray regional state of Ethiopia, 2023: A prospective cohort study	BMC Pediatr	OR	Among children admitted to an Ethiopian hospital with severe acute malnutrition shortly after the Tigray War, early recovery was more likely in those who received oral rather than IV antibiotics, those who did not require blood transfusion or IV fluids, and those who tolerated oral rather than nasogastric tube feeds
Emergency Care in Limited Resource Settings (ECLRS)	Adal O [34]	Mortality of traumatic chest injury and its predictors across sub-Saharan Africa: Systematic review and meta-analysis, 2024	BMC Emerg Med	RE	This systematic review and meta-analysis aimed to assess mortality rates and identify factors contributing to death from traumatic chest injuries in sub-Saharan Africa. The pooled mortality rate was calculated at 9%, with several clinical factors identified as significant predictors
	Ahmed AA [35]	The role of telemedicine in emergency department triage and patient care: A systematic review	Cureus	RE	This systematic review looked at the breadth and depth of telemedicine in the ED via six RCTs. They conclude telemedicine has potential in triage and non-critical care, but call for more research in high acuity settings and improved regulations and policies
	Arora D [36]	Efficacy of slow negative pleural suction in thoracic trauma patients undergoing tube thoracostomy: A randomized clinical trial	Injury	OR	Continuous wall suction was superior to standard water seal for chest tube management in trauma patients
	Bonnet G [37]	Cost-effectiveness of COVID rapid diagnostic tests for patients with severe/critical illness in low- and middle-income countries: A modeling study	PLoS Med	OR	The use of rapid diagnostic tests is cost-effective in severe COVID cases when there is high prevalence of COVID and rapid testing is available, regardless of country income level. In low-income, resource-limited settings, testing is most cost-effective when there is high prevalence of COVID and less cost-effective when there is low prevalence of disease
	Colunga-Pedraza JE [38]	Overcoming challenges to reduce time to antibiotic therapy in febrile neutropenic children: Insights from a Mexican center	Hematol Transfus Cell Ther	OR	A “Golden Hour” intervention significantly shortened time to antibiotic treatment and improved clinical outcomes in pediatric hematology-oncology patients with febrile neutropenia. It highlights an effective intervention that can be implemented in other low- and middle-income countries
	Conradi N [39]	Solar-powered O2 delivery for the treatment of children with hypoxemia in Uganda: A stepped-wedge, cluster randomized controlled trial	Lancet	OR	A stepped-wedge randomized controlled trial of 2409 participants conducted in rural Ugandan hospitals demonstrated a statistical reduction in mortality of children 48 h from detection of hypoxemia when provided access to solar-powered oxygen concentrator systems
	Daihimfar F [40]	A comparison of the effects of acupressure and music on venipuncture pain intensity in children: A randomized controlled clinical trial	Pain Res Manag	OR	This randomized controlled trial involving 180 children aged three to six years in an Iranian emergency department found that both music and acupressure significantly reduced pain from venipuncture compared to no intervention, with music showing the greatest effect
	Endeshaw D [41]	Mortality and its predictors in abdominal injury across sub-Saharan Africa: Systematic review and meta-analysis	BMC Emerg Med	RE	A meta-analysis helped determine predictive factors of mortality for abdominal trauma in sub-Saharan Africa, which could help tailor strategies to reduce mortality in this patient population
	Gyedu A [42]	Differences in trauma care between district and regional hospitals and impact of a trauma intake form with decision support prompts in Ghana: A stepped-wedge cluster randomized trial	World J Surg	OR	Utilizing a decision-support trauma intake form with clinical prompts improved initial trauma care both in district and regional hospitals in Ghana
	Jaramillo GD [43]	Implementation of an early attention strategy to reduce emergency room overcrowding in an academic institution in Colombia: A pilot study	Int J Emerg Med	OR	In this retrospective observational study, a physician-in-triage protocol for moderate complexity patients (triaged 3 on 1–5 scale) reduced patient length of stay in a Colombian emergency department
	Karamian A [44]	Incidence of intracranial bleeding in mild traumatic brain injury patients taking oral anticoagulants: A systematic review and meta-analysis	J Neurol	RE	This meta-analysis demonstrates a high rate of intracranial hemorrhage, 9.4%, from mild traumatic brain injury (TBI) for patients on oral anticoagulation
	Kefyalew M [45]	Improving the time to pain relief in the emergency department through triage nurse-initiated analgesia: A quasi experimental study from Ethiopia	Afr J Emerg Med	OR	Nurse-led analgesia in an Ethiopian emergency department reduced the time to pain medication administration and improved patient satisfaction
	Legesse AT [46]	Validation of the Raja Isteri Pengiran Anak Saleha Appendicitis (RIPASA) scoring system for the diagnosis of acute appendicitis among Ethiopian patients: A multi-institutional observational study	BMC Surg	OR	This study validates the RIPASA scoring system as a reasonable alternative for diagnosing acute appendicitis in resource-limited settings where imaging, particularly ultrasound, is not always accessible
	Quake SYL [47]	The current status and challenges of prehospital trauma care in low- and middle-income countries: A systematic review	Prehospital Emergency Care	RE	This systematic review summarizes the current variable development of prehospital trauma care systems in LMICs, highlights common barriers to development and implementation, and presents successful case studies which can inform future efforts in low-resource settings
	Rahmani C [48]	Can plethysmographic capillary refill time predict lactate during sepsis?: An observational study from Morocco	Afr J Emerg Med	OR	In a prospective observational cohort, capillary refill time was assessed visually and with a pulse oximeter and found to be a cost-effective, noninvasive way to measure tissue perfusion. Capillary refill time was found to correlate with serum lactate levels in patients with sepsis or septic shock
	Rahnemayan S [49]	Shortened NIHSS for rapid stroke assessment in emergency care settings	Neurologist	OR	The NIHSS-8, a shorter version of the original stroke scale, is a potentially reliable and efficient alternative to the NIHSS-11 for the diagnosis of stroke in emergency care settings
	Ramamoorthy T [50]	Diagnostic value of point-of-care ultrasound-guided assessment of relative afferent pupillary defect in adult ocular trauma patients presenting to the emergency department: A prospective cohort study	J Ultrasound Med	OR	Emergency physician performed POCUS showed high sensitivity and specificity in detecting relative afferent pupillary defect (RAPD) in a prospective cohort of adult ocular trauma patients in an Indian emergency department
	Ranjbar Hameghavandi MH [51]	Challenges in traumatic spinal cord injury care in developing countries: A scoping review	Front Public Health	RE	This scoping review identified 82 articles summarizing challenges in traumatic spinal cord injury (TSCI) prevention and management in low- and middle-income countries
	Shakir M [52]	Temporal delays in the management of traumatic brain injury: A comparative meta-analysis of global literature	World Neurosurg	RE	This global meta-analysis assessing time to intervention after a TBI found significant delays in care across prehospital and intrahospital settings, with disparities related to country-level income, region, and healthcare payment system
	Sri-on J [53]	Missed opportunity to diagnose palliative care need among older emergency department patients in a middle-income country: A retrospective study	Open Access Emerg Med	OR	This study from Thailand identifies and characterizes significant missed opportunities to assess the need for palliative care in the emergency department for patients suffering with life-limiting illness. The provision of palliative care has the potential to improve overall quality of life, decrease pain, and decrease medical costs, particularly during end-of-life emergency care
	Stephen S [54]	Clinico-epidemiological profile, trends, and health-related outcomes of snakebite victims: A 1 year prospective study from eastern India	Wilderness Environ Med	OR	A prospective observational study of individuals presenting with snakebites to a tertiary hospital in Eastern India summarized factors associated with these bites and clinical outcomes. Based on these results, the team highlights the need for programs to improve public knowledge of first aid measures to care for snakebites and the importance of seeking timely medical care for consideration of potentially life-saving antivenom
	Tanaanantarak P [55]	Clinical characteristics associated with pediatric traumatic intracranial hemorrhage	Chin J Traumatol	OR	Clinical predictors including injury mechanism and severity, reduced GCS, signs of skull fracture on exam, and vomiting ≥ 3 times were significantly associated with intracranial hemorrhage on CT in pediatric head trauma patients in Thailand
	Wang L [56]	The effectiveness and implementation of psychological first aid as a therapeutic intervention after trauma: An integrative review	Trauma Violence Abuse	RE	Psychological First Aid (PFA) has been shown to have a positive effect on anxiety and adaptive functioning following traumatic exposure, but significant variability in format and implementation complicates the development of best practices for PFA
	Yang J [57]	Current status of emergency medical service use in ST-segment elevation myocardial infarction in China: Findings from China Acute Myocardial Infarction (CAMI) registry	Int J Cardiol	OR	This study examines emergency medical service (EMS) utilization in Chinese patients with ST-segment elevation myocardial infarction (STEMI). It analyzes patient characteristics, response times, and survival outcomes to assess the effectiveness of pre-hospital care strategies
Emergency Medicine Development (EMD)	Behnoush AH [58]	Effects of intravenous lipid emulsion administration in acute tramadol poisoning: A randomized controlled trial	J Emerg Med	OR	This randomized controlled trial assessed the use of intravenous lipid emulsion (ILE) therapy among patients with acute tramadol poisoning and a GCS of less than 12. It found that those who underwent ILE administration had significantly reduced seizure frequency, length of hospitalization, and higher GCS when compared to controls receiving treatment with saline
	Koko JAB [59]	The ABCDE approach: Evaluation of adherence in a low-income country	BMC	OR	The authors researched the use of the ABCDE approach while managing trauma patients in three of the major trauma centers in Khartoum, Sudan. The study showed only 37.9% adherence to the approach, suggesting a need for improved education and implementation of trauma management guidelines
	Michalski K [60]	Advancing access to care: An assessment of the prehospital system in Senegal	World J Surg	OR	The Senegalese experience in prehospital care highlights the urgent need for restructuring of nascent prehospital systems in low-resource settings for efficient and quality emergency medical services
	Nasar Isfahani M [61]	Comparing the efficacy of intravenous morphine versus ibuprofen or the combination of ibuprofen and acetaminophen in patients with closed limb fractures: A randomized clinical trial	BMC Emerg Med	OR	This study assessed pain management modalities in isolated closed extremity fractures in adults. Superior pain control at 60 min was found in the IV ibuprofen-acetaminophen group as compared with IV morphine or IV ibuprofen alone

**TABLE 3 |:** Historical GEMLR article screening and scoring, by year.

GEMLR year	Main screened	Gray screened	Main scored	Gray scored	Full reviewed	Reviewed DHR	Reviewed ECLRS	Reviewed EMD

2005	48	-*	48	-*	10	6	3	1
2006	1004	-*	130	-*	25	11	11	3
2007	865	-*	104	-*	30	12	12	6
2008	5247	-*	229	-*	26	6	16	4
2009	26,124	-*	384	-*	24	13	9	2
2010	6936	– **	200	0	27	6	14	7
2011	7924	– **	206	7	24	0	17	7
2012	4818	– **	224	15	28	6	18	4
2013	8768	– **	434	4	24	5	16	3
2014	6376	– **	477	13	25	4	19	2
2015	12,435	– **	723	14	24	3	19	2
2016	13,890	– **	716	11	19	1	16	2
2017	17,722	– **	848	11	21	1	16	4
2018	19,102	– **	517	19	25	4	21	0
2019	23,321	– **	356	49	16	1	11	4
2020	35,970	– **	364	21	20	2	13	5
2021	44,839	-*	444	-*	23	3	16	4
2022	58,510	7755	524	33	37	7	27	3
2023	58,291	11,035	825	37	56	13	35	8
2024	46,714	12,575	473	16	33	5	24	4
Total	398,904	31,365	8226	250	517	109	333	75

Note:

* No gray literature search was done

** Gray search was done, but numbers were not recorded.

## Data Availability

The data that support the findings of this study are available in PubMed at https://pubmed.ncbi.nlm.nih.gov/. These data were derived from the following resources available in the public domain: Data S1, https://www.gemlr.org/.

## Appendix A

### Members of the 2024–25 Global Emergency Medicine Literature Review Group (in alphabetical order)

Abdirahman Abdulle, MBBS, Department of Emergency Medicine, Makerere University, Kampala, Uganda; Halley J Alberts, MD, Department of Emergency Medicine, Prisma Health - University of South Carolina; Kimonia Bih Awanchiri, MD, Department of Emergency Medicine, Cameroon Baptist Convention Health Services, Cameroon; Emily Bartlett, MD, Department of Emergency Medicine, University of New Mexico; Tal Berkowitz, MD, MPH, Department of Pediatrics, Division of Emergency Medicine, Emory University School of Medicine, Children’s Healthcare of Atlanta; Nidhi Bhaskar, BA, Warren Alpert Medical School of Brown University, Providence, RI^*^; Corey B. Bills, MD, MPH, Department of Emergency Medicine, University of Colorado, Aurora, CO^*^; Joseph Bonney, MBChB, MPH, MScDM, MGCPS, Department of Emergency Medicine, Komfo Anokye Teaching Hospital, Kumasi, Ghana^*^; Morgan C. Broccoli, MD, MPH, MSc, Department of Emergency Medicine, Brigham and Women’s Hospital; Agatha Brzezinski, MD, MPH, DTM&H, Department of Emergency Medicine, University of Washington; Whitney K. Bryant, MD, MPH, MEd, Department of Emergency Medicine, University of Cincinnati; Jonathan Chan, MD, Department of Emergency Medicine, University of California, Davis Health; Joseph D. Ciano, DO, MPH, MS, Department of Emergency Medicine, Hospital of the University of Pennsylvania; Cassandra Clay, DO, MHA, Department of Emergency Medicine, Cherokee Nation, Tahlequah OK; Ly Cloessner, MD, MSPH, Washington University School of Medicine, St. Louis, MO; Amanda Collier, MD, MPH, DTM&H, Department of Emergency Medicine University of Ottawa and Departments of Emergency Medicine, Queen’s University; Nanaba A. Dawson-Amoah, MBChB, MBA, MGCS, Department of Emergency Medicine, Greater Accra Regional Hospital, Ghana; Kamoga Dickson, MBChB, Department of Emergency Medicine, Makerere University, Kampala Uganda; Jonathan W. Dyal, MD, MPH, Department of Emergency Medicine, Johns Hopkins University School of Medicine; Christian Engelen, MD, Department of Emergency Medicine, Lauf/Pegnitz Hospital, Lauf/Pegnitz, Germany; Brandon Friedman, MD, Department of Emergency Medicine, Atrium Health-Carolinas Medical Center, Charlotte, NC; Stephanie Chow Garbern, MD, MPH, Department of Emergency Medicine, Warren Alpert Medical School of Brown University, Providence, RI^*^; Juliette Gerardo, MD, Department of Emergency Medicine, University of California, Davis Medical Center; Mindi Guptill, MD, Department of Emergency Medicine, Loma Linda University Health, Loma Linda, CA.; Reid Haflich, MD, MPH, DTM&H, Department of Emergency Medicine, University of Washington Global Emergency Medicine and Rural HeEmergency Medicine; Alison S. Hayward, MD, MPH, Department of Emergency Medicine, Warren Alpert Medical School of Brown University, Providence, RI^*^; Braden J. Hexom, MD, Department of Emergency Medicine, Rush University Medical Center, Chicago, IL; Taylor S. Hickey, University of Pikeville: Kentucky College of Osteopathic Medicine; Anneka Hooft, MD, MPH, Department of Emergency Medicine, University of California, San Francisco; Emmanuel Oluyinka Idowu, MBBS, MSc, MPH, MD, MRCEM, FACEP, FAAEM, FMCEM Department of Emergency Medicine, Faculty of Clinical Sciences, College of Medicine, University of Ibadan, Ibadan, Nigeria; Ashley A. Jacobson, MD, Department of Emergency Medicine, Yale University School of Medicine, New Haven, Connecticut, USA; Aqeel Jawahir, MD, Department of Emergency Medicine, Medical University of South Carolina; Jennifer E. Jones, MBBS, Department of Emergency Medicine, UMass Chan Medical School - Baystate Medical Center; Vinay N. Kampalath, MD, MSc, DTM&H, Department of Pediatrics, University of Pennsylvania School of Medicine, Children’s Hospital of Philadelphia, Philadelphia, PA; Elizabeth M. Keating, MD, MSPH, Division of Pediatric Emergency Medicine, University of Utah, Salt Lake City, UT; Sean M. Kivlehan, MD, MPH, Department of Emergency Medicine, Brigham and Women’s Hospital, Boston, MA; and Harvard Humanitarian Initiative, Cambridge, MA^*^; Arthi S. Kozhumam, MScGH, Medical Scientist Training Program, Northwestern University Feinberg School of Medicine; Colleen E. Laurence, MD, MPH, Department of Emergency Medicine, Boston University School of Medicine and Boston Medical Center, Boston, MA; Joseph Leanza, MD, MPH, Department of Emergency Medicine, Boston University School of Medicine and Boston Medical Center; Samuel Lewis, MD, MPhil, Department of Emergency Medicine, University of Washington; J. Austin Lee, MD, MPH, DTMH, Department of Emergency Medicine, Indiana University School of Medicine, Bloomington, IN; Gideon Loevinsohn, MD, PhD, Department of Emergency Medicine, Mass General Brigham Department of Emergency Medicine; Parker Maddox, MD, MS, Department of Emergency Medicine, University of Cincinnati; Mallika Manyapu, MD, MPH, Department of Emergency Medicine, George Washington University; Michael Mathelier, BS, University of Florida College of Medicine; Kevin J. Mercer, PharmD, MPH, The University of Texas at Austin College of Pharmacy, Austin, TX; Rmaah Memon, MD, Department of Emergency Medicine, University of Pennsylvania; Kevin Molyneux, MD, MPH, DTMH, Department of Emergency Medicine, Texas Tech University Health Sciences Center of El Paso; Dana Naamani, MD, MPH, Department of Emergency Medicine, Duke University, Durham NC; Benjamin D. Nicholson, MD, Department of Emergency Medicine, Virginia Commonwealth University, Richmond, VA^*^; Hannah Ofosua Owusu, BSc, MBChB, MGCS, Department of Emergency Medicine, St. Joseph Hospital, Koforidua-Ghana; Gerard M. O’Reilly, MBBS, FACEM, MPH, MBiostat, PhD, School of Public Health and Preventive Medicine, Monash University, Melbourne, Victoria, Australia^*^; Mayur Patel, MD, MBA, MPH, Department of Emergency Medicine, University of Florida; Nana Serwaa Agyeman Quao, MBChB, MGCS, MPH, Department of Emergency Medicine, Korle Bu Teaching Hospital, Accra, Ghana; Sarah Rapaport, MD, Department of Emergency Medicine, Mass General Brigham, Boston, MA; Chris A. Rees, MD, MPH, Division of Pediatric Emergency Medicine, Emory University School of Medicine and Children’s Healthcare of Atlanta; Charlotte Roy, MD, MPH, Department of Emergency Medicine, University of Southern California, Los Angeles, CA; Megan M. Rybarczyk, MD, MPH, Department of Emergency Medicine, University of Pennsylvania School of Medicine, Philadelphia, PA^*^; Jessica Schmidt, MD, MPH, Department of Emergency Medicine, University of Wisconsin-Madison; Megan L. Schultz, MD, MA, Medical College of Wisconsin, Milwaukee, WI^*^; Anand Selvam, MD, MSc, DTM&H, Department of Emergency Medicine, Yale University, New Haven, CT^*^; Erin F. Shufflebarger, MD, MSPH, Department of Emergency Medicine, University of Alabama at Birmingham; Yusra Shakil, MBChB, Department of Emergency Medicine, Levy Mwanawasa University Teaching Hospital; Branden Skarpiak, MD, DTM&H, Department of Emergency Medicine, University of Colorado School of Medicine; Cheyenne Smith, MD, Department of General Pediatrics, Children’s Hospital of Philadelphia; Jonathan M. Strong, MD, MPH, Department of Emergency Medicine, Brigham and Women’s Hospital; Harvard Medical School; Janet Jebichii Sugut, MD, MMED, Department of Emergency Medicine, Kenyatta National Hospital, Nairobi Kenya; Fadhila Tekka, MD, MMED, FPECC, Department of Pediatrics and Child Health, Muhimbili National Hospital, Tanzania; W. Tyler Winders, MD, MPH, Hannibal Regional Hospital, Hannibal, MO^*^; Ann Wolski, MD, Department of Emergency Medicine, University of Cincinnati; Natalie Yabalwashi, MD, Department of Emergency Medicine, University Teaching Hospital, Zambia.

* Denotes advisor.
