# Supplementary material for: Global Emergency Medicine: A Scoping Review of the Literature From 2024
Source: Acad Emerg Med. 2025 Dec 23;33(3):e70208. doi: 10.1111/acem.70208 (PMC12925323; doi:10.1111/acem.70208)
Supplement: Supplementary file 1 — Data S1: acem70208‐sup‐0001‐Supinfo1.pdf. [file ACEM-33-0-s006.pdf]

Global Emergency Medicine Literature Review

2024-2025 Reviewer Procedure Manual

Updated *October 27, 2024*

TABLE OF CONTENTS

Mission Statement……………………………………………………..Page 3
Introduction……………………………………………………..……..Page 3
Roles, Positions and Authorship…………………………………Page 4
Expectations of Reviewers………………………………………..Page 6
Conflict of Interest Policy………………………………………….Page 8
Plagiarism Policy……………………………………………………Page 10
Search Strategy……………………………………………………..Page 11
Screening Guidelines………………………………………………Page 13
Scoring Guidelines…………………………………………………. Page 19
Reviewing Articles…………………………………………………Page 27
Appendix 1……………………………………………………………Page 31
Appendix 2……………………………………………………………Page 35

MISSION STATEMENT

The Global Emergency Medicine Literature Review (GEMLR) aims to highlight and disseminate high quality global emergency medicine research in the fields of emergency medicine development, disaster and humanitarian response, and emergency care in resource-limited settings. The goals of the review are to illustrate best practices, stimulate additional research, and promote further professionalization of the field of global emergency medicine.

INTRODUCTION

The rise of emergency medicine as a unique specialty continues to advance rapidly around the globe. As national medical organizations and ministries of health recognize this growing field, individual hospitals and municipalities are developing their own emergency departments and emergency medical care systems. At the same time, the interest in global emergency medicine (GEM) (formerly known as International Emergency Medicine) has grown exponentially in recent years. International sections, interest groups, and committees exist within each of the major US emergency medicine organizations and, in some cases, have become the largest special interest section within these organizations. In addition, the number of GEM fellowship positions in the US has increased significantly over the past decade, with more expected to open in the near future. Finally, the availability of international educational experiences, including electives abroad, has been shown to positively affect medical students' ranking of emergency medicine residency programs.

The domain of GEM encompasses a broad spectrum of activities, often without clear boundaries, though generally falling into one of two categories: (1) development of emergency medical care systems in both developed and developing nations, and (2) provision of direct patient care during humanitarian and disaster relief efforts. Emergency medical care development encompasses clinical, educational, and systems components at both the national and local levels. Included within this rubric are efforts as diverse as developing a national disaster relief plan, creating a city-wide EMS system, and training rural health practitioners to provide basic emergency services. The field of humanitarian relief also encompasses several different domains and is rapidly becoming more organized through critical analyses of operations executed. Both humanitarian relief agencies and donor governments are looking closely at ways to improve humanitarian response, including the conduct of rapid needs assessments, the deployment of personnel and supplies, and ongoing monitoring and evaluation.

One of the barriers to the development of the nascent field of GEM has been the lack of an easily accessible literature base that can be used to guide its development. Both research articles and communiqués of value to various stakeholders in GEM are spread throughout the general medical literature, the emergency medicine literature, the public health literature, the health policy/health systems literature, and the humanitarian/disaster literature. Some publications with GEM relevance may exist only in the gray literature in the form of assessments or reports by international agencies, national or local governments, foundations, donor agencies, financial institutions, or non-governmental organizations. Furthermore, GEM research may be published in a variety of different languages, limiting its accessibility to all audiences.

Recognizing the need for a clear and accessible body of literature to guide the growing field of

GEM, the Global Emergency Medicine Literature Review (GEMLR) Group was formed in 2005 (formerly known as the International Emergency Medicine Literature Review Group). It has been published annually in the journal *Academic Emergency Medicine*, and the review gathers together a number of articles from disparate sources, presenting the ones chosen as having specific relevance or value to one of the previously described domains of GEM. We hope that it will continue to be a valuable resource, both for GEM practitioners and academic researchers, for many years to come.

ROLES, POSITIONS AND AUTHORSHIP

Editorial Board: The editorial board will have primary responsibility for making decisions for the GEMLR. It will be chaired by the editor-in-chief, and will generally attempt to make decisions by consensus, though the editor-in-chief may choose to hold an official vote when necessary. The editor-in-chief, managing editors, editors, technical editor, the targeted systematic review team lead and advisors will be full voting members of the editorial board. The editorial board members, including advisors, will review the final draft of the manuscript and provide comments prior to submission.

Editor-in-Chief: Maintains overall responsibility for the review as a whole and represents the “public face” of the review to outside organizations. The editor-in-chief will chair all meetings of the editorial board and will be responsible for communicating regularly with the full editorial board regarding any new developments or major decisions regarding the review. The editor-in-chief works with the managing editor to select new reviewers each year based on the applications they submit. The editor-in-chief works closely with the managing editor and technical editor to produce the review each year and will have responsibility for filling both those roles if either becomes temporarily or permanently unavailable until such time as a replacement can be found. The editor-in-chief will be last/senior author on the print version of the main review and the systematic review. The editor-in-chief’s term will be for 3 years.

Managing Editor: Maintains primary responsibility for ensuring that the review adheres to its predetermined timeline, working with individual editors to ensure that material is submitted correctly and on time. The managing editor will not directly supervise reviewers but will be responsible for taking on the role of an editor in supervising their reviewers if an editor becomes temporarily or permanently unavailable until a replacement can be found. The managing editor will work with the editor-in-chief to select new reviewers each year based on their applications. The managing editor will work closely with the technical editor to ensure that the mechanics of the review always run smoothly. In addition, the managing editor will have primary authorship of the print version of the review and will be listed as first author of the review. The Managing Editor with be listed as a co-author on the systematic review as well. A prerequisite for serving as managing editor will include at least one year of superior prior service as an editor. The managing editor's term will be for 2 years.

Assistant Managing Editor: The assistant managing editor works alongside the Managing Editor. The assistant managing editor will support and receive training from the current managing editor and will replace the managing editor at the end of the 2 year term. The assistant managing editor will be listed as the second author of the review.

Technical Editor: Assists the managing editor in the technical aspects of review production, including performing the initial search, dividing up citations for screening, dividing up articles for scoring, creating tables and figures, etc. The technical editor will be listed as the next to last author on the review. The technical editor term will be for 3 years.

Assistant Technical Editor: This position is intended to be filled every third year and will assist the technical editor in the technical aspects of review production and works alongside the technical editor. The assistant technical editor will support and receive training from the current technical editor and will replace the managing editor at the end of their term after 3 years. The technical editor will not be listed as an author but will be added to the collaboration group.

Editors: Primary responsibility for supervising a small group of junior and senior reviewers, including ensuring the quality and timeliness of all material submitted by reviewers. ***If at any point a reviewer becomes temporarily or permanently unavailable, the editor will take over their responsibilities until such time as a replacement can be found.*** All editors will also participate as active members of the editorial board. Editors will be elected initially by a vote of the editorial board and will be given the opportunity each year thereafter to continue in their role unless they are found by the editorial board to be derelict in their duties or unless limited by term limits.

The prerequisite for serving as an editor will include at least one (preferably two or more) years of superior service as a reviewer. There are three designations for editors. Assistant editors are newly promoted senior reviewers. Editors are returning editors with one year of editorial service. Senior editors are those with two or more years of service as an assistant editor/editor. Senior editors will be responsible for mentoring and supervising the assistant editors through the steps of the review process, in particular reviewing their final screening and scoring files. All editors of each designation will be listed as authors on the print version of the review. Senior editors will be listed first, editors next, and assistant editors next. Within each designation, author positions will generally be in alphabetical order.

Reviewer: Reviewers will be primarily responsible for performing the screening, scoring, and writing up of individual reviews. They will be selected initially by the managing editor and editor-in-chief based on their applications and will be given the opportunity each year thereafter to continue in their role unless they are found by their editor or the managing editor to be derelict in their duties. Reviewers will be designated as junior reviewers during their first year on the review, and as senior reviewers each year thereafter. A team of alternate reviewers is also selected annually and they are trained in screening and scoring practices. Alternate reviewers will be ready to take over for any reviewer who is unable to complete their term and will receive priority when review positions open up in future years. Senior reviewers, junior reviewers, and alternate reviewers will all be acknowledged in the print version of the review as members of the GEMLR but will not be listed as authors.

Targeted Systematic Review (SR) Team:
The SR team consists of one SR team lead, an assistant SR team lead, an SR advisor, and several SR team members. The SR team lead serves on the editorial board. The assistant lead/lead position is a two-year combined position, with advancement from assistant lead to lead after the first year. The lead will be the first author, the assistant lead will be the second author. The assistant lead will support and receive training from the current lead. The SR advisor is a one-year position held by the previous SR lead. The remaining SR team members will be listed as authors in alphabetical order. The GEMLR managing editor will be included as an author, and the editor-in-chief will be the last author. All SR team positions will be elected by a vote of the editorial board.

Advisor: After serving for a full year, any member of the editorial board or any SR team member can choose to become an inactive member of the GEMLR, referred to as advisor. They will be full members of the editorial board with the ability to vote in all editorial board decisions, but they will not be responsible for supervising reviewers during that year. They will be acknowledged in the print version of the review as members of the GEMLR but will not be listed

as authors.

Social Media & Marketing Director: The Social Media & Marketing Director will be primarily responsible for promoting the GEMLR through its website, Twitter feed, social media networks, and the Emergency Medicine community. They will be selected by the managing editor and editor-in-chief based on an application process and will be given the opportunity each year thereafter to continue in their role unless they are found by the editorial board to be derelict in their duties. The Social Media & Marketing Director will create a schedule of tweets to highlight articles identified by the GEMLR as well as the work done by the GEMLR team. The social media manager will be responsible for monitoring and analyzing the impact of GEMLR's social media efforts. This involves tracking metrics such as engagement rates, follower growth, reach, and interactions across various platforms. This person's role involves not only maintaining the creation and sharing of infographics across existing platforms but also exploring new avenues, such as Instagram and LinkedIn, to widen GEMLR's audience reach. Continuing the development of GEMLR's podcasts or collaborating with other relevant podcasts in the medical field will be a core aspect of the social media manager. The social media manager will maintain a comprehensive list of relevant accounts to tag in GEMLR's social media posts. This list will include other EM organizations such as SAEM GEMA, AFEM, IFEM, ACEP, CAEP, CAEP Global EM, and others within the emergency medicine community. By effectively tagging these organizations, the social manager will help GEMLR foster collaboration, increase visibility, and facilitate broader discussions within the emergency medicine field. They will be acknowledged in the print version of the review as members of the GEMLR but will not be listed as authors.

See Appendix 2 for additional details including term limits

EXPECTATIONS OF REVIEWERS:

- This review is a collaborative effort between a team of approximately 9 editors and several dozen reviewers. This requires a great deal of ongoing communication in order to ensure that everyone is working in concert. As such, **it is expected that reviewers will check their email at least daily** during the course of the review to ensure they are kept abreast of any new developments.
- **It is expected that reviewers will submit screening lists, scoring lists, and reviews on or before each of the deadlines assigned.** We cannot emphasize enough how important this is for the review. If one person is late with any of their assignments, it holds up the entire review. Even relatively brief delays can add up quickly, and too many delays may mean missing our publication deadlines for *Academic Emergency Medicine*. Calendar links/reminders will be sent with each assignment and can be added to google calendar or iCal or other calendar applications. We recommend that you put all the deadlines in your calendar so that you can prepare for them in advance.
- If you anticipate being out of the country or on a busy rotation, it is important to plan ahead and complete your assignments ahead of schedule. **If you know in advance that you may have limited email access at any time, please contact your editor well in advance to work out a plan**. **If an emergency arises and you realize that you will not be able to make one of the deadlines, alert your editor right away so that alternative arrangements may be made.**
- The editors will be tracking when reviewers turn in assignments throughout the year. A consistent pattern of late assignments will count against a reviewer being invited back the following year. In fact, **late assignments are the number one reason why reviewers have not been invited back in years past.**
- We expect reviewers to ask for help early when they don't understand a step in the

process or for any other reason, and to let their team editor know early if they will not be able to complete their assignment by a given deadline. **Asking for help is not a sign of weakness, and is strongly encouraged by this review.** While most questions can be resolved by email, editors will also be available by phone or skype to answer more complicated questions. If a reviewer feels they need more assistance than is available from their editor, they should next contact the Assistant Managing Editor, Managing Editor, and Editor-in-Chief (in that order).

- A brief note about authorship. Our dozens of team members are crucial to the review but we cannot list all reviewers as authors for our publication. The first author will be the managing editor, the second author will be the assistant managing editor, the last author will be the editor-in-chief, and the next to last author will be the technical editor. The remaining author positions will generally be assigned to the other editors in alphabetical order, starting with the senior editors, then editors, then assistant editors. The managing editor and editor-in-chief *will have the flexibility to promote an editor to a higher author* order if, and only if, they take on additional objective responsibility during the year, such as filling in for a reviewer who leaves mid-year. As members of the Global Emergency Medicine Literature Review Group, all reviewers will be listed in the acknowledgement section of the article. Though limitations imposed by *Academic Emergency Medicine* prevent us from listing all reviewers individually as authors, they will also typically be listed as collaborators in PubMed. It is still appropriate for all reviewers to include this review as an official publication on their CV, as shown below:

    - Becker TK, Hansoti B, Bartels S, Bisanzo M, Jacquet GA, Lunney K, Marsh R, Osei-Ampofo M, Trehan I, Lam C, Levine AC; the Global Emergency Medicine Literature Review (GEMLR) Group. Global Emergency Medicine: A Review of the Literature From 2015. *Acad Emerg Med.* 2016; **23** (10): 1183-1191. *(member of the Global Emergency Medicine Literature Review Group)*

CONFLICT OF INTEREST POLICY FOR EDITORS AND REVIEWERS
Adapted from the Annals of Emergency Medicine COI Policy

Purpose:
To describe the policy and practice for the management of editor and reviewer conflicts of interest involving *GEMLR.*

Policy:
GEMLR believes that all editorial board members, advisors, and reviewers acting on behalf of *GEMLR* have a fiduciary duty to *GEMLR*. A conflict of interest may occur when an editorial board member or reviewer has personal or outside financial, business, or professional interests, or other responsibilities that conflict with their duties to *GEMLR*, including decisions and reviews of manuscripts submitted to *GEMLR*. Editorial board members and reviewers are required to declare to *GEMLR* any actual or potential conflicts of interest.

Practice:

1. *Definitions*
    a. Conflict of Interest: Any situation or transaction in which a person has a direct or indirect interest such that he/she may realize a personal benefit from the situation or transaction. This includes decisions to publish or not publish a manuscript.
    b. Financial conflict of interest includes more than nominal compensation from an entity that has a financial interest or stake in the subject of a publication or other activity of *GEMLR*. Nominal in value means no more than twenty-five dollars ($25.00). Direct interest includes ownership by you or a member of your immediate family, or an investment in a concept, product or another party developing those concepts or products. Indirect interests involve consulting or accepting any type of compensation for work involving the concept or product or any company involved in the concept or product. Indirect interest also includes receipt of external funding or grants from either commercial or governmental or nonprofit funding agencies, such as NIH and others.
    c. Non-financial conflict of interest includes personal (friendship, family members, co-workers, political, religious), and intellectual (competing research, fiduciary responsibilities) interests that would make a reasonable reader, author, reviewer, or editor feel misled or deceived if not disclosed. These include friendship with an author, interest in competing organizations, competing research interests, or political beliefs or religious beliefs that could be perceived by a reasonable reader of *GEMLR* to interfere with your objectivity.
2. *Procedures*
    a. General: Annually, members of the *GEMLR* editorial board participating in the review will provide information regarding their potential conflicts of interest. This will include a financial conflicts of interest statement, as defined above.
    b. Reviewers: If a reviewer is assigned an article to score or review in which there may be a potential conflict of interest as outlined above, they should notify their editor immediately. The editor will promptly review the potential conflict and determine whether the reviewer may continue to score or review the article or whether it should be reassigned to another reviewer. The editor will notify the assistant managing editor and managing editor in writing of their decision on this matter.
    c. Editors: The screening, scoring, and review for any articles co-authored by a member of the editorial board will need to be reviewed by at least 3 senior staff to ensure that they do indeed meet the same objective and stringent standards

that we would expect for any articles. If these 3 senior staff unanimously agree that the screening, scoring, and review are objectively accurate, then the article may be included. If any of the 3 senior staff do not feel this objective standard was met, then the article should be excluded. The 3 senior staff should include one member of the GEMLR editorial board plus 2 members chosen from the following: managing editor, assistant managing editor and editor-in-chief. If additional senior staff are needed, then additional members of the editorial board can be selected for this purpose.

d. Complaints: A complaint regarding a potential conflict of interest of a reviewer will be referred to the managing editor who will review the circumstances and send a written assessment of the merits of the complaint to the editor-in-chief. A complaint regarding a potential conflict of interest of an editor will be referred to the managing editor who will review the circumstances and send a written assessment of the merits of the complaint to the editor-in-chief. The editor-in-chief will make a final decision on management of the conflict and a written summary will be sent to the GEMLR Editorial Board for record keeping.

e. GEMLR Articles: All GEMLR articles, including the main review and previous targeted systematic reviews authored by the GEMLR SR group, will be excluded from the current Review.

PLAGIARISM POLICY FOR EDITORS AND REVIEWERS

Adapted from the John Hopkins Plagiarism Policy

Purpose:

To describe the policy and practice for the management of plagiarism committed by editors and reviewers of *GEMLR.*

Policy:

GEMLR believes that all editorial board members, editors and reviewers acting on behalf of *GEMLR* have a duty to *GEMLR* to prevent plagiarism. Plagiarism may occur when an editorial board member or reviewer takes another individual's work and attributes the work as his or her own. Editorial board members and reviewers are required to declare to *GEMLR* any occurrences of plagiarism and to uphold the ethical integrity of the review. The definition of plagiarism *GEMLR* uses comes from the John Hopkins Bloomberg School of Public Health. The original source for the definition can be found at the following link:

http://www.jhsph.edu/academics/degree-programs/master-of-public-health/current-students/JHSPH-ReferencingHandbook.pdf **Practice:**

1. *Definition*
    a. Plagiarism is defined as "…taking for one's own use the words, ideas, concepts or data of another without proper attribution. Plagiarism includes both direct use or paraphrasing of the words, thoughts, or concepts of another without proper attribution. Proper attribution includes: (1) use of quotation marks or single-spacing and indentation for words or phrases directly taken from another source accompanied by proper reference to that source and (2) proper reference to any source from which ideas concepts or data are taken even if the exact words are not reproduced." (The John Hopkins Bloomberg School of Public Health Policy and Procedures Memorandum Students-1 Academic Ethics: October 2006).
2. *Procedures*
    a. General: Members of the *GEMLR* editorial board participating in the review will annually agree to *GEMLR*'s plagiarism policy.
    b. Reviewers: All reviewers will receive *GEMLR*'s policy on plagiarism prior to the start of the review and will be asked to affirm they have read and agreed to follow the policies regarding plagiarism.
    c. Editors: All editors will receive *GEMLR*'s policy on plagiarism prior to the start of the review and will be asked to affirm they have read and agreed to follow the policies regarding plagiarism. Editors will be responsible for ensuring reviews are not only well written and accurate but also adhere to our plagiarism policy. At a minimum, editors should compare the review to the original article to make sure that portions of the original article have not been directly copied. Editors will also be encouraged to run the reviews through an online plagiarism checking software, such as Viper (available free at http://www.scanmyessay.com) or another similar program of their choice.
    d. Reporting: Any incident of plagiarism committed by a reviewer should be reported to the managing editor who will review the circumstances and send a written assessment of the merits of the report to the editor-in-chief. The editor-in-chief will make a final decision on management of the situation and a written summary will be sent to *GEMLR* staff for record keeping.

SEARCH STRATEGY

Main Review:

Each year, we search the medical literature for all publications in the past calendar year pertaining to Global Emergency Medicine. We do this search in two phases: the first phase includes January – August, and the second phase includes September – December. We use PubMed for our main search, specifying a series of “emergency medicine” terms and “international” terms that we have refined over the years. We are always open to suggestions about new terms that we should include.

We limit our PubMed search to languages in which our reviewers are fluent, which varies slightly each year. We also exclude news articles, letters to the editor, commentaries, and editorials, and limit the search to human research only. Some articles that do not fit these criteria may occasionally still pass through the filters, but then should be removed at the screening stage.

In addition to the main PubMed search, we also perform a “hand search” of journals that tend to have a high concentration of GEM articles, based on the number of articles from these journals that have been selected for prior reviews (**currently:** Bulletin of the World Health Organization, Prehospital and Disaster Medicine, Lancet, and the African Journal of Emergency Medicine). We screen all the articles in these journals for the given year, regardless of whether they were picked up in our PubMed search.

All the citations identified by our main PubMed search and hand search are divided into a series of Word documents. Each Word document will have a file name that is based either on the language of the citations in it (for our main PubMed search) or on the journal it came from (for our hand search). The English list of citations is generally quite long, and is therefore divided into several dozen separately numbered Word documents. These Word documents are then distributed evenly among the reviewers, so that each reviewer has a similar number of citations to screen.

Gray Literature:

We also conduct a search of the gray literature. The purpose of the gray literature review is to identify practice-changing articles not captured by our standard literature review, and may include guidelines, reports, or reviews. One dedicated editor and one reviewer will review the gray lit sources. The current gray literature source list is listed below. This list is reviewed and updated based on feedback from the gray lit team and sources that have not produced relevant articles in the past three years will be recommended for removal by the editorial board.

Epicentre	https://epicentre.msf.org/publications
International Rescue Committee	https://www.rescue.org/reports-and-resources
United Nations U	https://collections.unu.edu/
MSF	https://www.msf.org/reports-and-publications
IMC	
ICRC	https://www.icrc.org/en/resource-centre/result?f=document_type%3A%22Publication%22&sort=date+desc
IFRC	www.ifrc.org
Centre for Research on the Epidemiology of Disasters	https://www.cred.be/
Centers for Disease Control and Prevention	https://www.cdc.gov/
World Health Organisation	https://www.who.int/
Partners in Health	https://www.pih.org/

SCREENING GUIDELINES

The first task for reviewers will be to screen the citation lists generated by our search in order to find articles that are truly relevant to Global Emergency Medicine. The screening process is often the most confusing aspect of the review, partly because the boundaries of the field of GEM are not clearly defined. For the purposes of this review, we split up the field of GEM into three categories: Emergency Care in Resource Limited Settings (ECLRS), Emergency Medicine Development (EMD), and Disaster and Humanitarian Relief (DHR). The types of articles to be included in each of these categories will be described below. If a citation does not clearly fall into one of these categories, then it does not belong in this review.

Once you receive your citation lists, reviewers should divide them into two separate files and send these back to your editor **(by emailing them AND by uploading them to the appropriate folder in the Google Drive)**. The first file should include the original file name with the word KEEP at the end, and the other file should include the original file name with the word TOSS at the end. For instance, if the original file was named “H1 English8.doc”, the reviewer should return two separate files to their editor. The first would be called “H1 English8 KEEP.doc” and the second would be called “H1 English8 TOSS.doc”.

Reviewers should cut and paste citations from your original file into each of these two new files based on the screening criteria below. **In general, the TOSS file should have 10-20 times as many articles as the KEEP file.** This is because most citations will not be relevant to GEM and should be placed in the TOSS file. The entire citation, including the PMID number at the very bottom, should be included, as this will be important for the next phase of the review. **Once completed, each team’s editor will review the screening files and report any disagreements to the managing editor.**

Below are a set of general rules for screening citations, followed by specific rules for each of our three categories of articles. Afterwards are examples of how to categorize two different citations. Reviewers should read the instructions below carefully and email your editor with specific questions.

<u>General Rules for Screening Citations</u>

1) Global Emergency Medicine research does not include all emergency medicine research conducted outside of North America. The article must be relevant to those individuals with a specific interest in global emergency medicine, not simply to emergency medicine practitioners around the globe. For instance, a study conducted in Italy on the sensitivity of MRI for detecting aortic dissection would probably be relevant to emergency physicians practicing in Europe, the United States, and other developed countries. However, it is not specific to the field of global emergency medicine, and therefore should not be included in our review. Due to the large volume of articles related to the COVID-19 pandemic, articles on COVID-19 are only kept if they have specific relevance to LMICs and emergency medicine (ECRLS) as per our general criteria.

2) The citations included in our review should fall into one of the following three categories: **Emergency Care in Resource Limited Settings (ECLRS), Disaster and Humanitarian Response (DHR), and Emergency Medicine Development (EMD)**. Below are listed specific examples of articles that would fit or not fit into each of these categories.

3) In general, we are looking for original research or review articles that contribute something new to the global emergency medicine literature base. Most letters to the editor, commentaries, editorials, news articles, case studies, or general descriptive reports should

not be included in the review. When screening citations, a good rule of thumb is that *if the citation does not have an abstract, it probably does not belong*, no matter how interesting the title (this does not apply to the gray literature screening). Furthermore, if the abstract does not seem to describe an original research study or a review of original research studies, it also should probably be cut.

Specific Criteria for Each Category

Emergency Care in Low Resource Settings (ECLRS)

Emergency Care in Low Resource Settings (ECLRS) encompasses emergency care in settings that lack the standard healthcare resources of high-resource or high-income settings. Our goal is to include practice-changing research articles that have the potential to improve the clinical practice of emergency care in settings with limited resources, globally.

Emergency care includes the acute clinical management of medical, surgical, and psychiatric conditions that require immediate intervention to prevent death or serious impairment of the health (disability) of an individual. It includes pre-hospital care as well as care in the initial time period of an individual's encounter with the health system that is included within the scope of practice of Emergency Medicine as occurs in systems where it is recognized as a formal medical specialty.

Keep: Citations related to trauma care, acute medical care, triage, or pre-hospital care in Low Resource Settings. Low Resource Settings are defined as populations or geographic regions in which there is limited access to or capacity for providing care, whether due to lack of funds, limitations in medication/equipment/personnel, or lack of infrastructure. *Most articles will come from low- and middle-income countries, but Low Resource Settings exist even within high-income countries*. Low-resource settings may include remote or rural areas that have poor access to healthcare services, areas of limited healthcare services in urban regions due to poverty or limited ability to access high-resource healthcare systems. Studies may include diagnostic, therapeutic and prognostic studies and reviews of studies, as well as studies on risk factors for specific acute conditions if it seems relevant to the emergent management of the condition.

Toss: Articles that describe or are performed in High Resource Settings regardless of country income level. *Studies involving high-resource utilization in an otherwise LMIC should be tossed*, such as MRI studies in large urban cities (e.g., Wuhan, Delhi), or services that are unavailable to the majority of the population, either due to cost, insurance, distance, or exclusion of particular religious or ethnic groups. Toss articles on the prevention and management of chronic medical conditions (e.g., chronic HIV care). Most immunization studies would also not be relevant. Articles on acute care or trauma care that mainly have relevance to high-income countries or are conducted in High Resource Settings within a low- or middle-income country should not be included either.

DHR (Disaster and Humanitarian Response)

Keep: Citations on the care of civilian populations in conflict, military humanitarian operations that involve the care of affected civilian populations, studies on disaster response that are applicable to a variety of settings, and health care of IDPs/refugees in camp settings (focusing on the acute phase, but also including the process of resettlement or repatriation).

Include articles on outbreaks, as long as the outbreaks themselves constitute a humanitarian crisis requiring an international humanitarian response or the outbreaks are part of an ongoing humanitarian crisis; we will include immunization studies specifically related to containing or preventing outbreaks that meet the above definition.

Toss: Citations on the care of military combatants only, disaster studies that are only

applicable to high-income countries, and studies on the care of IDPs/refugees after repatriation or resettlement. Also, toss studies related to mental health issues in disaster survivors that aren't clearly applicable to the immediate post-disaster response.

EMD (Emergency Medicine Development)

Keep: Citations on the development of emergency medicine as a specialty or emergency medical care systems in countries without fully developed EM systems. This includes development of emergency care training programs, development of pre-hospital emergency care or trauma systems, development of emergency care referral networks, and on development of pandemic response systems. Include EM development articles from all countries as long as the articles discuss EM development issues that are also broadly relevant to middle and low-income countries. This could also include international comparisons between countries of different income levels. Also include articles on EM training programs that focus on training individuals to work in resource-limited settings, regardless of the state of EM development in that country.

Toss: Articles purely related to emergency medicine development in countries with well-developed EM systems, or articles related to health system development that don't specifically involve emergency, trauma or pre-hospital care.

EXAMPLES OF HOW TO SCREEN CITATIONS

EXAMPLE #1

1: Ann Emerg Med. 2009 Oct;54(4):561-567.e2. Epub 2009 Jun 28.

Randomized clinical trial comparing a patient-driven titration protocol of intravenous hydromorphone with traditional physician-driven management of emergency department patients with acute severe pain.

Chang AK, Bijur PE, Davitt M, Gallagher EJ.

Department of Emergency Medicine, Albert Einstein College of Medicine, Montefiore Medical Center, Bronx, NY 10467, USA. achang@montefiore.org

STUDY OBJECTIVE: We test the null hypothesis that the "1+1" hydromorphone patient-driven protocol is clinically and statistically equivalent in safety and efficacy to that of traditional physician-driven administration of opioids for emergency department (ED) treatment of acute severe pain. METHODS: This was a prospective randomized clinical trial of nonelderly adults presenting to an urban academic ED with acute pain of sufficient severity to warrant intravenous (IV) opioids in the judgment of the attending physician. Patients randomized to the 1+1 hydromorphone patient-driven protocol received 1 mg IV hydromorphone followed by a second 1-mg dose 15 minutes later if the patient responded affirmatively to the question, "Do you want more pain medication?" Patients in the physician-driven group received any IV opioid in the dose chosen by the ED attending physician, with any additional analgesia provided at the discretion of that physician. The primary outcome was the difference in improvement in pain between the 2 groups at 60 minutes, as measured by a validated and reproducible numeric rating scale. Secondary outcomes included incidence of oxygen desaturation, hypoventilation, hypotension, bradycardia, nausea, vomiting, pruritus, and use of naloxone. RESULTS: The mean decrease in numeric rating scale pain scores for the 1+1 hydromorphone patient-driven group was 5.6 versus 4.5 in the physician-driven group. The difference of 1.1 numeric rating scale units (95% confidence interval 0.3 to 1.9) was statistically significant but fell 0.2 numeric rating scale units short of the 1.3 numeric rating scale unit threshold required to attain clinically significant efficacy. Safety profiles were similarly satisfactory in both groups. Ninety-four percent of the 1+1
hydromorphone patient-driven group achieved adequate analgesia (as defined by the patient) within 60 minutes of protocol initiation. CONCLUSION: The 1+1 hydromorphone patient-driven protocol is statistically superior and at least as clinically efficacious and safe as traditional physician-driven treatment of ED patients with acute severe pain. More than 9 of 10 patients randomized to the study protocol achieved satisfactory pain control, as defined by the patient, within an hour or less.

Publication Types:
Research Support, Non-U.S. Gov't

EXAMPLE #2

Effect of the rural rescue system on reducing the mortality rate of landmine victims: a prospective study in Ilam Province, Iran.

Saghafinia M, Nafissi N, Asadollahi R.

Trauma Research Center, Baqiyatallah University of Medical Sciences, Tehran, Iran.

BACKGROUND: In several Iranian provinces, there are large numbers of landmines that threaten the lives of many civilians. Ilam is one of the most polluted areas with 1,086 injuries from landmines between 1989 to 1999, with an overall mortality rate of 36.4%. A remarkable number of deaths occurred before the injured were conveyed to the hospital. In this survey, the effects of on trauma outcome of the use of prehospital trauma life support provided by trained paramedics and rural health workers as first responders were examined. METHODS: In an interventional, prospective study, 4,834 persons (general physicians, nurses, rural health workers, and emergency technicians, high- and low-educated people, layperson villagers, and nomads) were trained in one level of advanced (for general physicians and nurses) and four levels of basic life support courses during two years (2000-2001). Following the training, the data from 288 landmine victims who were referred to the main hospital in Ilam (trauma center) were registered prospectively (2001-2005). The effects of prehospital trauma life support training were assessed by using the Injury Severity Scale (ISS) score and prehospital physiologic severity (PSS) score. RESULTS: There were 288 injuries from landmines in the Mehran region between 2002 and 2005. The mean ISS score was 20.3 with a median of 13. Forty percent were severely injured with an ISS score >15. Of the injured who received prehospital care at the Mehran Emergency Center, the mean value of the PSS scores was 6.40, which improved to 7.43 in the hospital (p = 0.01; 95% CI for difference -0.72 to -0.45), in comparison with 5.97 in the injured who were conveyed to Ilam Hospital directly (mean of ISS was approximately equal in both groups). The total mortality rate was 27% between 2001 and 2005. CONCLUSIONS: Prehospital educations and training help improve PSS scores and reduce the death toll of landmine accidents in the remote areas.

Publication Types:
Research Support, Non-U.S. Gov't

PMID: 19591306 [PubMed - indexed for MEDLINE]

APPROACH TO EXAMPLE 1

By Jennifer Chan, GEMLR Advisor

Start with just reading this article's title and abstract if available. Here is an example thought process (no need to write it out)

- RCT- strong study design- but this is relevant to the score phase, not the screening phase
- How does this pertain to IEM? EM development? Disaster/refugee response? Pandemic response? Provision of EM service in resource limited settings? It doesn't clearly indicate this in the title. *Next, we should look at the abstract.*
- Even after reading the abstract, the article still does not seem to pertain to IEM. This falls into the exclusion noted in the manual, *"The article should have relevance to those interested in the field of international emergency medicine, not simply to international practitioners of emergency medicine"*

Make the decision to TOSS.

APPROACH TO EXAMPLE 2

By Jennifer Chan, GEMLR Advisor

Appears to have an international perspective

- Rural rescue system may be related to pre-hospital care settings, and rural settings which may be provision of care in a resource limited setting.
- At this point the quality and rigor of the study is not relevant. This will be addressed in the scoring phase, which is not part of the screening process.
- This is an article that those interested in IEM may find interesting and/or relevant
- Mortality associated with pre-hospital care in a rural setting
- This may be considered a post-conflict humanitarian health issue.
- Other IEM providers may find this study interesting as it discusses the potential impact of life support courses on injury severity after landmines.

Make the decision to KEEP

SCORING GUIDELINES

At the scoring stage, each reviewer will receive two lists of citations that have been selected by the screening process for formal scoring. One will be the *primary* list, and the other the *secondary* list (each article is scored by two separate reviewers, which is why each reviewer will have two lists).

The first task will be to find the full text PDF for each of the citations on the **primary** list. This includes downloading appendices or supplemental files for qualitative studies. **Reviewers will be responsible for uploading their articles into the appropriate OR or RE Google Drive folder within 1 week of receiving their assigned lists of articles (see Timeline).** Most full-text PDFs can be found by searching PubMed through a university account, which will have access to most journals. If not available there, reviewers should also try emailing the corresponding author directly to ask for a copy of their article. Explain that the article is requested for the Global Emergency Medicine Literature Review. **If reviewers have any trouble locating an article by one of these means, they should notify their editor immediately – well before the deadline for uploading articles**. PDFs should be placed in the appropriate **Google Drive** folder within each editor's folder, including appropriate sub-folders (OR or RE).

In order to ensure that both the primary and secondary reviewers are using the same scoring criteria for a given article, the editors are expected to oversee which articles are classified as review and which are original research. The primary reviewer will still be responsible for finding the articles and uploading them (within one week of being assigned their scoring list) to their **Google Drive** folder. Editors will be responsible for scanning through the articles in the **Google Drive** folders uploaded by their reviewers and ensuring that they have: 1) actually uploaded all their articles on time, and 2) put them in the correct **Google Drive** folder. Then the reviewers will have several weeks to actually read their primary and secondary articles and score them.

The next task for citations on the Reviewer's **primary** list is to determine whether the articles meet the GEMLR criteria for justice and equity in authorship and ethics. At least one local author must be listed for articles to be scored. A paper without a local author will be excluded from the review. This will likely be more common for RE articles in which no specific country is identified. Additionally, local IRB approval or similar local governmental or approval authority, specifically mentioned, must be included for the article to be scored. There are some infrequent exceptions to this, for example when a paper does not localize to a specific low- or middle-income country. Article PDFs not meeting these requirements should be placed in the folders for editor review.

The next step will be to enter the scoring data into the shared online scoring spreadsheets. **For each article on the primary list**, the primary reviewer should enter the last name and first initial of the first author, the article title, the abbreviated journal title, the category (EMD vs DHR), the type (**O**riginal **R**esearch versus **RE**view), and the team editor's initials. **For each article on the secondary list**, just enter the last name and first initial of the first author. See Tables 2 and 3 below for examples of how to enter data on the primary and secondary lists. Scoring of articles will be separated into original research articles and review articles. As the goal of the literature is to identify important work done in global emergency medicine over the preceding year, it is critical that we develop a system to assess and rank articles in an objective manner. Ideally, two separate reviewers should be able to read the same article and give it the exact same score, based on our scoring criteria. While there are many different scoring systems in existence for evaluating research articles, the heterogeneity of the articles contained in our search and the unique logistical concerns of GEM have led us to develop our own scoring

system.

Original Research vs Review Articles: To clarify, an original research (**OR**) article is a primary source. Typically, it is a scientific article written by the researchers who performed the study. The researchers describe their research question, purpose, methods, discussion, and conclusions (often under those headings). To contrast, a review article (**RE**) might include literature reviews, systematic reviews, meta-analyses, clinical guidelines, as well as articles about the ethics or conduct of research or education in an international setting. Both **OR** and **RE** articles can be placed in any of the 3 categories (EMD, DHR, ECLRS), depending on their focus.

Each article will be scored across 4 separate categories, which will be slightly different for Original Research versus Review Articles. For each of these categories, the article will be given a score depending on whether it contains specific elements, with 0 being the lowest score and 4, 5, or 6 being the highest score depending on the category. Articles can receive a total score of 0-20. **Table 4** provides the specific elements to be used for scoring original research articles, while **Table 5** focuses on review articles. Do not award partial points: It's either all in or all out with the exception of the 'Importance' and 'Impact' scores where explicitly allowed (see scoring sheets). For example, if a category is weighted with two points, you will either give those two points if you think that the article meets the criteria listed. If it doesn't, award zero points.

Under the "Impact" score, one point is awarded for explicit consideration of cost-effectiveness. A formal cost-effectiveness analysis is not required, however authors must demonstrate an understanding of the financial costs of implementing their research findings in their practice area."

Scores for each category are entered into the Article Scoring spreadsheets for both primary and secondary lists. For the primary list, enter scores into the columns labeled 1 (i.e. Clarity 1) and for your secondary list, enter scores into the columns labeled 2 (i.e. Clarity 2). Completed scoring lists should be returned to editors prior to the deadline.

Please note that for OR articles in which there is a discrepancy of more than 2 standard deviations from the mean difference of scores (1 standard deviation for RE articles), the editor will rescore the article independently and all three scores will be averaged together to determine the final score. The editor should provide feedback to reviewers as needed on the accuracy of their scoring during this process.

GRAY LITERATURE SCORING GUIDELINES

Scoring guidelines are generally as above with some modifications. Each article will be scored across 3 categories, with a maximum score of 12. Table 6 provides the specific elements to be used for scoring gray literature articles. All scored Gray Literature articles will be included in the supplementary database. The top-scoring article (or articles in case of a tie) each year will be formally reviewed by the GEMLR Gray Literature Reviewers according to the Reviewing Articles section below.

Table 2: Format for Entering Data for Primary List

GEMLR: Article Scoring for ORIGINAL RESEARCH ARTICLES

Author(Last F)	Title	Journal	Category (ECRLS, DHR, EMD)	Original Research or Review (OR, RE)	PMID	Editor Initials	Reviewer 1	Reviewer 2	1. Clarity (Rev 1)	1. Clarity (Rev 2)	2. Ethics (Rev 1)	2. Ethics (Rev 2)	3. Importance (Rev 1)	3. Importance (Rev 2)	4. Impact (Rev 1)	4. Impact (Rev 2)	Total score (Rev 1)	Total score (Rev 2)	Avg Overall Score (Both Rev)
Abdolazimi M	Predictive factors of short-term survival from acute myocardial infarction in early and late patients in Isfahan and Najafabad, Iran	ARYA Atheroscler	ECRLS	OR	27429625	IT	CB		5		4		4		3		16		
Al-Dubai S	Pre-hospital delays in ischemic stroke patients in a Malaysian tertiary hospital	Int J Stroke	ECRLS	OR	26865155	IT	CB		4		4		3		2		13		
Andrew S	Sources of organisational resiliency during the Thailand floods of 2011: a test of the bonding and bridging hypotheses	Disasters	DHR	OR	26272101	IT	CB		2		3		4		1		10		

Table 3: Format for Entering Data for Secondary List

GEMLR: Article Scoring for ORIGINAL RESEARCH ARTICLES

Author(Last F)	Title	Journal	Category (ECRLS, DHR, EMD)	Original Research or Review (OR, RE)	PMID	Editor Initials	Reviewer 1	Reviewer 2	1. Clarity (Rev 1)	1. Clarity (Rev 2)	2. Ethics (Rev 1)	2. Ethics (Rev 2)	3. Importance (Rev 1)	3. Importance (Rev 2)	4. Impact (Rev 1)	4. Impact (Rev 2)	Total score (Rev 1)	Total score (Rev 2)	Avg Overall Score (Both Rev)
Abdolazimi M						IT		IK		5		4		4		3		16	
Al-Dubai S						IT		IK		4		4		3		2		13	
Andrew S						IT		IK		2		3		4		1		10	

Guide for Evaluating Original Research Articles (OR)

For articles describing original research studies (including randomized controlled trials, observational studies, case series, cross-sectional surveys, and diagnostic studies) consider the following 4 measures of quality and impact listed in **Table 4**. This will yield a maximum possible score of 20 points. The best articles will have the highest scores. This rubric should be used for both quantitative and qualitative studies. **If unsure about the answer to one of the questions, do not award the point(s).**

Table 4: Original Research Article

Quality Measure	Question		Points
Design	Select One	Descriptive studies (including case studies and case series, natural observation studies and descriptive surveys) including qualitative studies which only report anecdotal statements without any further analysis	1 *-or-*
		Correlation studies (case control studies, prospective observational studies, retrospective studies) **or** qualitative studies that either coded responses or used a standardized survey/interview tool	2 *-or-*
		Non-randomized or non-blinded experimental studies **or** qualitative studies that both coded responses and used a standardized survey/interview tool	3 *-or-*
		Randomized, blinded experimental studies **or** qualitative studies using a validated, standardized survey or interview structure, systematic and reproducible coding and analysis with appropriate reporting of identified themes	4
	Correct statistical tests are used to analyze quantitative data. For qualitative data analysis, involves coding that identifies emerging themes and appropriate reduction and interpretation of data (for example, into appropriate diagrams, matrices or taxonomies)		__/1
	No obvious bias in the selection of the subjects or authors attempt to limit bias		__/1
Design Total			/ Out of max score 6
Ethics	Contribution from authors with a primary affiliation in the country(ies) that the research was either performed in or is specifically relevant to, and that author is the first and/or last author		__/1
	The study was approved by a local or international IRB, a government ministry, or a community group and the study clearly adheres to the Declaration of Helsinki (see Appendix 1)*		__/1
	Either written or verbal consent was obtained in the subject's own language or consent was waived by IRB		__/1
	The authors declare that they have no significant conflicts of interest.		__/1
Ethics Total			/ Out of a max of 4

**Importance**	The study results are not specific to one area or situation but are broadly generalizable to a variety of settings:<br>● at the regional/single-country level (1)<br>● at an international level (+1 = total of 2 points if important internationally)	__/2
	The topic being studied is an important one, in that it affects…:<br>● many people (1)<br>● especially vulnerable groups as defined at www.un.org/en/fight-racism/vulnerable-groups (1)	__/2
	The study is clearly relevant to the realm of global emergency medicine.	__/1
Importance Total	/ Out of a max of 5	
**Impact**	The study describes an intervention that may be feasibly implemented by practitioners in developing countries.	__/1
	The article considers cost-effectiveness (A formal cost-effectiveness analysis is not required, however authors must demonstrate an understanding of the financial costs of implementing their research findings in their practice area)	__/1
	The results would lead to a change in policy:<br>● at the regional/single-country level (1)<br>● at an international level (1) (+1 = total of 2 points if important internationally)	__/2
	The results of the research are likely to result in improved health of the affected population	__/1
Impact Total	/ Out of a max of 5	

* Do not automatically award credit for IRB approval to research using publicly available, de-identified databases. Authors must still report IRB approval or exemption from IRB approval in the manuscript.

Guide for Evaluating Review Articles (RE)

For review articles (including formal meta-analyses, systematic reviews, and descriptive reviews), consider the following 4 measures of quality and impact listed in **Table 5**. All measures are to be scored on a 0-5 basis with 0 being the lowest score obtainable and 5 being the highest. This will yield a maximum possible score of 20 points. The best articles will have the highest scores. **If unsure about the answer to one of the questions, do not award the point(s).**

Table 5: Scoring of Review (RE) Articles

**Quality Measure**	**Question**	**Points**
**Clarity**	The review has a clearly stated hypothesis or purpose.	__/2
	The authors provide sufficient background to put the results of the review into context.	__/1
	The review can be understood by someone with general medical or public health training.	__/1
	The authors use clear language and appropriate graphs, tables, and figures throughout the article.	__/1
Clarity Total	/ Out of max score 5	
**Design**	This is a formal meta-analysis or a systematic review that only includes studies with a control group.	__/2
	There is a clear, reproducible method for the selection of studies included in this review.	__/1
	Articles for this review were selected by at least two authors blinded to each other's selection.	__/1
	The data was aggregated and/or analyzed appropriately.	__/1
	Contribution from authors with a primary affiliation in the country(ies) that the research was either performed in or is specifically relevant to, and that author is the first and/or last author	__/1
Design Total	/ Out of max score 6	
**Importance**	The study results are not specific to one area or situation but are broadly generalizable to a variety of settings:<br>● at the regional/single-country level (1)<br>● at an international level (1) (+1 = total of 2 points if important internationally)	__/2
	The topic being studied is an important one, in that it affects…:<br>● many people (1)<br>● especially vulnerable groups as defined at www.un.org/en/fight-racism/vulnerable-groups (1)	__/2
	This is clearly relevant to the realm of Global Emergency Medicine.	__/1
Importance Total	/ Out of max score 5	

**Impact**	The results would lead to a change in policy:<br>● at the regional/single-country level (1)<br>● at an international level (1) (+1 = total of 2 points if important internationally)	__/2
	The article considers cost-effectiveness (A formal cost-effectiveness analysis is not required, however authors must demonstrate an understanding of the financial costs of implementing their research findings in their practice area)	__/1
	The results of the research are likely to result in improved health of the affected population	__/1
Impact Total	/ Out of max score 4	

Guide for Evaluating Gray Literature Articles (GR)

For gray literature articles, consider the following 3 measures of design, quality, and impact listed in Table 6. All measures are to be scored with 0 being the lowest score obtainable and a maximum possible score of 12 points. The best articles will have the highest scores. If unsure about the answer to one of the questions, do not award the point(s).

Table 6: Scoring of Gray Literature (GR) Articles

Quality Measure	Question	Points
**Design**	The article relies upon:<br>● expert consensus (1)<br>● review of existing literature (1)<br>● primary data (1) (= total of 3 points if all three)	__/3
**Design Total**	**/ Out of max score 3**	
**Importance**	The study conclusions or recommendations are not specific to one area or situation but are broadly generalizable to a variety of settings:<br>● at the regional/single-country level (1)<br>● at an international level (1) (+1 = total of 2 points if important internationally)	__/2
	The topic being studied is an important one, in that it affects…:<br>● many people (1)<br>● especially vulnerable groups* (1) (total of 2 points if both)<br>*defined at un.org/en/fight-racism/vulnerable-groups	__/2
	The article is clearly relevant to the realm of Global Emergency Medicine	__/1
**Importance Total**	**/ Out of max score 5**	
**Impact**	The results would lead to a change in policy:<br>● at the local/national level (1),<br>● at an international level (1) (+1=total of 2 points if important internationally)<br>Or:<br>● recommendations or interventions suggested in the article may be feasibly implemented by practitioners in developing countries (1) (1 point total)	__/2
	The article considers cost-effectiveness (A formal cost-effectiveness analysis is not required, however authors must demonstrate an understanding of the financial costs of implementing their research findings in their practice area)	__/1
	Adoption of the article's recommendations are likely to result in improved health of the affected population.	__/1
**Impact Total**	**/ Out of max score 4**	

SELECTING ARTICLES FOR FINAL REVIEW

After the scoring is completed for both the first and second half of the year, we will average the scores for each article from the primary and secondary reviewer, to create an average score for each article. These will be compiled into a single scoring spreadsheet. The top 5% of articles from each category (RE, OR, ECRLS, EMD, and DHR) will be determined, rounding down, to establish a cutoff score for each category. The difference between reviewer scores for each article are calculated and articles with scores above 2 standard deviations (1SD for review articles) are eligible for rescore IF a perfect rescore would result in the article then exceeding the inclusion cutoff score. Editors will then rescore these articles. A final list of articles is then chosen for full review (usually about 30-50 articles).

REVIEWING ARTICLES

Each reviewer will be assigned between one to three of these articles, for which they will write a full one page review. Each review should include a summary and commentary section. Additionally, each reviewer will be required to provide a draft tweet and 1-2 slides along with the article review that they submit.

<u>The summary section should include</u>:
1. Objective of the article
2. Brief summary of the methods
3. Results of the article

<u>The comment section should address:</u>
1. Strengths of the article's design
2. Limitations of the article's findings
3. Importance of the findings to the field of GEM
4. How the article fits into the context of prior research on the topic

Because each article will present its own challenges and raise specific questions during the review process, it is difficult to give precise instructions other than the general guidelines above on how to complete the reviews. We have included sample reviews below for two Review articles and two Original Research articles. New reviewers should also refer to the online supplement section for our prior published reviews for more examples. Editors should be contacted EARLY if reviewers have any questions at all about how to proceed or what to include in the write-up!

It is very important to follow our formatting template *exactly* as outlined below:

OR vs RE
ECLRS vs EMD vs DHR
<single blank line>
<bold> Author Last Name First Initials, et al *(include first six only, no comma between last name and first initial, but before "et al.")*. Title. Journal Name *(abbreviated)*. Year; Issue: Page. (*last page abbreviated: 105 to 109 written as 105-9; 105 to 115 written as 105-15)* </bold>
<single blank line>
<italic> One to two sentence summary. </italic>
<single blank line>
<bold>Summary:</bold> Summary of the article.
<single blank line>
<bold>Comment:</bold> Editorial assessment of the articles strength and weaknesses, comment on context and importance
<single blank line>
<italic> Name of reviewer, name of supervising editor </italic>

<u>Sample GEMLR Narrative/Summary Reviews</u>

REVIEW ARTICLES:

Brennan RJ, Sondorp E. Humanitarian aid: some political realities. BMJ. 2006; 333(7573):817-8.

The task of providing humanitarian relief has become increasingly politicized, with unfortunate consequences for those most in need.

Summary: In this comment piece, the authors review some of the political obstacles to providing humanitarian relief work. Focusing on the World Health Organization Eastern Mediterranean Region (EMRO), which has contributed around 60% of the world's refugee population and hosts the majority of the world's refugees, the authors highlight three problem domains faced by the humanitarian community: interagency coordination, documentation of humanitarian need, and security for aid workers. The authors cite the difficulty of dealing with organizations such as Hezbollah that may perform positive social functions as well as participate in activities shunned by the majority of the humanitarian community. They present the difficulties in gathering and presenting data posed by lack of host country government cooperation to grant access (as in Darfur) and government discrediting of data that is not politically comfortable (as by the U.S. and U.K. in relation to Iraqi casualties from the ongoing Iraq war). The third disturbing trend presented is the increasing violence directed specifically toward humanitarian workers and organizations, such as the killing of aid workers in Afghanistan and Sudan. The authors make a call for humanitarian workers to maintain their professionalism and to hold policy makers accountable for decisions that adversely affect populations.

Comment: While this is only a short review of the problems facing humanitarian relief efforts in the WHO Eastern Mediterranean Region, it draws significant attention to the problem. Due to the increased violence noted in the world today, greater efforts are needed to provide adequate and efficient humanitarian aid to those in need. While the authors did comment on the political obstacles to providing humanitarian aid in war-torn countries, their review unfortunately failed to discuss potential solutions.

Hobgood C, et al. International Federation for Emergency Medicine Model Curriculum for Medical Student Education in Emergency Medicine. Israeli Journal of Emergency Medicine – Vol. 9, No.2 July 2009:30-35.

An effort by the International Federation for Emergency Medicine (IFEM) to create an international model curriculum in Emergency Medicine for medical students.

Summary: With more than fifty countries now incorporating emergency medicine training into their core medical student curriculum, the IFEM believes there is a need for a global standard of training. The organization suggests a longitudinal education framework over four years of schooling. The authors, a group of emergency physicians and health professionals from the United States, Australia, Asia, and Europe are described as experts in international emergency medicine. Their target population is students in both developing nations without existing EM curriculum and countries that wish to further expand existing emergency medicine training programs. The suggested curriculum is expansive- ranging from basic life support to timely stroke care and the acute management of myocardial infarction. In addition to refining students' clinical skills, they suggest training in time management, EM research, and legal aspects of care. An example curriculum is proposed that includes didactic and skills training with metrics of performance indicators and outcome measures. In its entirety the paper acts as a mission statement for the IFEM and suggests that it will be active in not only establishing curriculum guidelines, but also in further developing and keeping these educational directives current.

Comment: Most EM physicians will agree that creating a core EM curriculum in medical schools is a necessary first step towards establishing and solidifying Emergency Medicine as a global specialty. The IFEM paper is successful in creating an exhaustive subject list for topics to be covered by educators worldwide. However, the article is limited by its methodology in that the process of achieving consensus around key learning objectives, performance indicators, and outcome measures is unclear. Further review and analysis of successful training models is also needed. For example, in Germany and Israel, medical students have learned from experienced providers in pre-hospital care and traumatology, respectively. Simulation training should also be considered as a novel training method to educate medical students internationally. IFEM has the potential to use these successful models in emergency medicine education to inspire and further refine their curriculum guidelines.

ORIGINAL RESEARCH ARTICLES:

Roudsari BS, Shadman M, Ghodsi M. Childhood trauma fatality and resource allocation in injury control programs in a developing country. BMC Public Health. 2006; 6:117.

Improving pediatric trauma mortality in developing countries requires attention to the particular timing and cause of death.

Summary: Citing the generally accepted concept of a trimodal temporal distribution of trauma mortality, the authors undertook a retrospective analysis of traumatic deaths among children under 15 years old in Tehran, Iran over one year. Using date from the official government agency to which all deaths are reported, they identified 419 deaths in children under 15 years old due to trauma. Due to data limitations on the exact time of death in pre-hospital cases, the authors defined the three phases as pre-hospital, ED, and hospital. The authors reported that 6% of the 419 cases were either homicide or suicide. They did not exclude these from their data set and did not specify the mechanism of death for these cases. Of the remaining 394 victims,
29

motor vehicle crash (MVC) injuries accounted for 211 (50%), burns 74 (18%), poisoning 24 (6%), and drowning 18 (4%). Overall 43% died in the pre-hospital setting, 20% in the ED, and 37% in hospital. The distributions were quite different among the four major causes, with over 80% of poisoning and drowning deaths occurring in the pre-hospital phase and 92% of burn deaths occurring in the hospital phase. 25% to 30% of MVCs and fall-related deaths occurred in the ED phase and approximately 40% occurred in the pre-hospital phase.

Comment: This study demonstrates some of the issues surrounding childhood trauma fatality in a developing country. Despite difficulties with the quality of records, this study supplies information useful for local planners. It also confirms prior studies of trauma death distribution in developing countries. The relatively high percentage of deaths in the ED in blunt trauma from falls and MVCs suggests an area for possible improvement in death rates through further analysis of ED trauma management. Unfortunately, one of the limitations of this data set was a lack of distinction among pre-hospital deaths between those dead at the scene and those dying during transport. As the authors point out, further studies of this sort will help planners most appropriately allocate limited resources.

Tanon A, Eholie S, Binan Y, et al. [Medical emergencies related to HIV/AIDS in tropical zones: a prospective study in Cote d'Ivoire (1999-2000)]. Med Trop (Mars). 2006; 66(2):162-6. <Original Article in French>

When it comes to medical emergencies in the developing world, infectious diseases still come first, especially where HIV prevalence is high.

Summary: This prospective, observational study aims to describe medical emergencies requiring admission to Treichville Hospital in Abidjan and to outline the impact of HIV infection among that cohort. All patients over 15 years old presenting to the medical or infectious services of the hospital and requiring immediate medical attention were enrolled in the study over 8 months in 1999, excluding surgical or gynecologic emergencies. A standardized survey of demographic information, medical history, symptoms and prior medical care was administered; consent for HIV testing was obtained, and serologic screening for HIV in conjunction with a complete diagnostic work-up undertaken. Among the 400 patients enrolled, 312 (78%) tested positive for HIV. Most of the patients complained of chronic symptoms, especially protracted fever and chronic diarrhea, and had been previously evaluated and referred by satellite health centers. The most frequent chief complaints of HIV positive patients included: generalized decline (62%), fever (50%), diarrhea (39%), and cough (20%). Infectious diseases represented the majority of final diagnoses with gastrointestinal, pulmonary, and neurologic systems most frequently affected.

Comment: Practitioners working in high-HIV prevalence, resources-limited settings face many challenges in treating HIV-positive patients. This study describes the most common presenting complaints, physical signs, diagnostic findings, and final diagnoses of HIV-positive patients presenting to the medical and infectious disease services of a referral hospital in Cote D’Ivoire. The study offers an exhaustive description of common clinical presentations and a re-iteration of the need for aggressive evaluation of opportunistic infections among HIV-positive patients in resource-limited areas.

30

APPENDIX 1: DECLARATION OF HELSINKI

Adopted by the 18th WMA General Assembly, Helsinki, Finland, June 1964
and amended by the:
29th WMA General Assembly, Tokyo, Japan, October 1975
35th WMA General Assembly, Venice, Italy, October 1983
41st WMA General Assembly, Hong Kong, September 1989
48th WMA General Assembly, Somerset West, Republic of South Africa, October 1996
52nd WMA General Assembly, Edinburgh, Scotland, October 2000
53rd WMA General Assembly, Washington DC, USA, October 2002 (Note of Clarification added)
55th WMA General Assembly, Tokyo, Japan, October 2004 (Note of Clarification added) 59th WMA General Assembly, Seoul, Republic of Korea, October 2008
64th WMA General Assembly, Fortaleza, Brazil, October 2013

1. The World Medical Association (WMA) has developed the Declaration of Helsinki as a statement of ethical principles for medical research involving human subjects, including research on identifiable human material and data. The Declaration is intended to be read as a whole and each of its constituent paragraphs should be applied with consideration of all other relevant paragraphs.
2. Consistent with the mandate of the WMA, the Declaration is addressed primarily to physicians. The WMA encourages others who are involved in medical research involving human subjects to adopt these principles. General Principles
3. The Declaration of Geneva of the WMA binds the physician with the words, “The health of my patient will be my first consideration,” and the International Code of Medical Ethics declares that, “A physician shall act in the patient's best interest when providing medical care.”
4. It is the duty of the physician to promote and safeguard the health, well-being and rights of patients, including those who are involved in medical research. The physician's knowledge and conscience are dedicated to the fulfilment of this duty.
5. Medical progress is based on research that ultimately must include studies involving human subjects.
6. The primary purpose of medical research involving human subjects is to understand the causes, development and effects of diseases and improve preventive, diagnostic and therapeutic interventions (methods, procedures and treatments). Even the best-proven interventions must be evaluated continually through research for their safety, effectiveness, efficiency, accessibility and quality.
7. Medical research is subject to ethical standards that promote and ensure respect for all human subjects and protect their health and rights.
8. While the primary purpose of medical research is to generate new knowledge, this goal can never take precedence over the rights and interests of individual research subjects.
9. It is the duty of physicians who are involved in medical research to protect the life, health, dignity, integrity, right to self-determination, privacy, and confidentiality of personal information of research subjects. The responsibility for the protection of research subjects must always rest with the physician or other health care professionals and never with the research subjects, even though they have given consent.
10. Physicians must consider the ethical, legal and regulatory norms and standards for research involving human subjects in their own countries as well as applicable international norms and standards. No national or international ethical, legal or regulatory requirement should reduce or eliminate any of the protections for research subjects set forth in this Declaration.
11. Medical research should be conducted in a manner that minimises possible harm to the environment.
12. Medical research involving human subjects must be conducted only by individuals with the

appropriate ethics and scientific education, training and qualifications. Research on patients or healthy volunteers requires the supervision of a competent and appropriately qualified physician or other healthcare professional.

13. Groups that are underrepresented in medical research should be provided appropriate access to participation in research.

14. Physicians who combine medical research with medical care should involve their patients in research only to the extent that this is justified by its potential preventive, diagnostic or therapeutic value and if the physician has good reason to believe that participation in the research study will not adversely affect the health of the patients who serve as research subjects.

15. Appropriate compensation and treatment for subjects who are harmed as a result of participating in research must be ensured. Risks, Burdens and Benefits

16. In medical practice and in medical research, most interventions involve risks and burdens. Medical research involving human subjects may only be conducted if the importance of the objective outweighs the risks and burdens to the research subjects.

17. All medical research involving human subjects must be preceded by careful assessment of predictable risks and burdens to the individuals and groups involved in the research in comparison with foreseeable benefits to them and to other individuals or groups affected by the condition under investigation. Measures to minimize the risks must be implemented. The risks must be continuously monitored, assessed and documented by the researcher.

18. Physicians may not be involved in a research study involving human subjects unless they are confident that the risks have been adequately assessed and can be satisfactorily managed. When the risks are found to outweigh the potential benefits or when there is conclusive proof of definitive outcomes, physicians must assess whether to continue, modify or immediately stop the study.

Vulnerable Groups and Individuals

19. Some groups and individuals are particularly vulnerable and may have an increased likelihood of being wronged or of incurring additional harm. All vulnerable groups and individuals should receive specifically considered protection.

20. Medical research with a vulnerable group is only justified if the research is responsive to the health needs or priorities of this group and the research cannot be carried out in a non-vulnerable group. In addition, this group should stand to benefit from the knowledge, practices or interventions that result from the research. Scientific Requirements and Research Protocols

21. Medical research involving human subjects must conform to generally accepted scientific principles, be based on a thorough knowledge of the scientific literature, other relevant sources of information, and adequate laboratory and, as appropriate, animal experimentation. The welfare of animals used for research must be respected.

22. The design and performance of each research study involving human subjects must be clearly described and justified in a research protocol.

The protocol should contain a statement of the ethical considerations involved and should indicate how the principles in this Declaration have been addressed. The protocol should include information regarding funding, sponsors, institutional affiliations, potential conflicts of interest, incentives for subjects and information regarding provisions for treating and/or compensating subjects who are harmed as a consequence of participation in the research study. In clinical trials, the protocol must also describe appropriate arrangements for post-trial provisions.

Research Ethics Committees

23. The research protocol must be submitted for consideration, comment, guidance and approval to the concerned research ethics committee before the study begins. This committee must be transparent in its functioning, must be independent of the researcher, the sponsor and any other undue influence and must be duly qualified. It must take into consideration the laws

and regulations of the country or countries in which the research is to be performed as well as applicable international norms and standards but these must not be allowed to reduce or eliminate any of the protections for research subjects set forth in this Declaration.
The committee must have the right to monitor ongoing studies. The researcher must provide monitoring information to the committee, especially information about any serious adverse events. No amendment to the protocol may be made without consideration and approval by the committee. After the end of the study, the researchers must submit a final report to the committee containing a summary of the study's findings and conclusions.

Privacy and Confidentiality
24. Every precaution must be taken to protect the privacy of research subjects and the confidentiality of their personal information. Informed Consent
25. Participation by individuals capable of giving informed consent as subjects in medical research must be voluntary. Although it may be appropriate to consult family members or community leaders, no individual capable of giving informed consent may be enrolled in a research study unless he or she freely agrees.
26. In medical research involving human subjects capable of giving informed consent, each potential subject must be adequately informed of the aims, methods, sources of funding, any possible conflicts of interest, institutional affiliations of the researcher, the anticipated benefits and potential risks of the study and the discomfort it may entail, post-study provisions and any other relevant aspects of the study. The potential subject must be informed of the right to refuse to participate in the study or to withdraw consent to participate at any time without reprisal. Special attention should be given to the specific information needs of individual potential subjects as well as to the methods used to deliver the information.
After ensuring that the potential subject has understood the information, the physician or another appropriately qualified individual must then seek the potential subject's freely-given informed consent, preferably in writing. If the consent cannot be expressed in writing, the non-written consent must be formally documented and witnessed.
All medical research subjects should be given the option of being informed about the general outcome and results of the study.
27. When seeking informed consent for participation in a research study the physician must be particularly cautious if the potential subject is in a dependent relationship with the physician or may consent under duress. In such situations informed consent must be sought by an appropriately qualified individual who is completely independent of this relationship.
28. For a potential research subject who is incapable of giving informed consent, the physician must seek informed consent from the legally authorised representative. These individuals must not be included in a research study that has no likelihood of benefit for them unless it is intended to promote the health of the group represented by the potential subject, The research cannot instead be performed with persons capable of providing informed consent, and the research entails only minimal risk and minimal burden.
29. When a potential research subject who is deemed incapable of giving informed consent is able to give assent to decisions about participation in research, the physician must seek that assent in addition to the consent of the legally authorised representative. The potential subject's dissent should be respected.
30. Research involving subjects who are physically or mentally incapable of giving consent, for example, unconscious patients, may be done only if the physical or mental condition that prevents giving informed consent is a necessary characteristic of the research group. In such circumstances the physician must seek informed consent from the legally authorised representative. If no such representative is available and if the research cannot be delayed, the study may proceed without informed consent provided that the specific reasons for involving subjects with a condition that renders them unable to give informed consent have been stated in the research protocol and the study has been approved by a research ethics committee. Consent to remain in the research must be obtained as soon as possible from the subject or a

legally authorised representative.
31. The physician must fully inform the patient which aspects of their care are related to the research. The refusal of a patient to participate in a study or the patient's decision to withdraw from the study must never adversely affect the patient-physician relationship.
32. For medical research using identifiable human material or data, such as research on material or data contained in biobanks or similar repositories, physicians must seek informed consent for its collection, storage and/or reuse. There may be exceptional situations where consent would be impossible or impracticable to obtain for such research. In such situations the research may be done only after consideration and approval of a research ethics committee.

Use of Placebo
33. The benefits, risks, burdens and effectiveness of a new intervention must be tested against those of the best proven intervention(s), except in the following circumstances: Where no proven intervention exists, the use of placebo, or no intervention, is acceptable; or Where for compelling and scientifically sound methodological reasons the use of any intervention less effective than the best proven one, the use of placebo, or no intervention is necessary to determine the efficacy or safety of an intervention and the patients who receive any intervention less effective than the best proven one, placebo, or no intervention will not be subject to additional risks of serious or irreversible harm as a result of not receiving the best proven intervention. Extreme care must be taken to avoid abuse of this option.

Post-Trial Provisions
34. In advance of a clinical trial, sponsors, researchers and host country government should make provisions for post-trial access for all participants who still need an intervention identified as beneficial in the trial. This information must also be disclosed to participants during the informed consent process.

Research Registration and Publication and Dissemination of Results
35. Every research study involving human subjects must be registered in a publicly accessible database before recruitment of the first subject.
36. Researchers, authors, sponsors, editors and publishers all have ethical obligations with regard to the publication and dissemination of the results of research. Researchers have a duty to make publicly available the results of their research on human subjects and are accountable for the completeness and accuracy of their reports. All parties should adhere to accepted guidelines for ethical reporting. Negative and inconclusive as well as positive results must be published or otherwise made publicly available. Sources of funding, institutional affiliations and conflicts of interest must be declared in the publication. Reports of research not in accordance with the principles of this Declaration should not be accepted for publication.

Unproven Interventions in Clinical Practice
37. In the treatment of an individual patient, where proven interventions do not exist or other known interventions have been ineffective, the physician, after seeking expert advice, with informed consent from the patient or a legally authorised representative, may use an unproven intervention if in the physician's judgement, it offers hope of saving life, re-establishing health or alleviating suffering. This intervention should subsequently be made the object of research, designed to evaluate its safety and efficacy. In all cases, new information must be recorded and, where appropriate, made publicly available.

APPENDIX 2: POLICY ON TERM LIMITS, ROLE ELIGIBILITY, PROMOTION and EDITOR EVALUATION AND TERMINATION

Purpose:

- To clarify eligibility, selection process, and term limits for all publication author-level GEMLR positions
- To ensure advancement opportunities for reviewers
- Term limits are in place to ensure diversity and evolution in leadership positions

The GEMLR Editorial Board consists of one Editor-in-Chief, one Managing Editor, one Assistant Managing Editor, one Technical Editor, one Assistant Technical Editor, Main Review Editors and Advisors as well as the Targeted SR Team Lead.

1. Main Review
    a. Editor-in-Chief (EIC)
        i. Eligibility: Current or previous ME (previous: only if current advisor)
        ii. Voting: Editorial Board (candidates abstain)
        iii. Term Limit: 3 years
    b. Managing Editor (ME)
        i. Eligibility: Current or previous AME (previous: only if current advisor)
        ii. Voting: Editorial Board (candidates abstain)
        iii. Term Limit: 2 years
    c. Assistant Managing Editor (AME)
        i. Eligibility: Current or previous Editor or SR Lead (previous: only if current advisor)
        ii. Voting: Editorial Board (candidates abstain)
        iii. Term Limit: 2 years
    d. Editor
        i. Eligibility: Current Senior Reviewer or SR Lead/Team Member
        ii. Voting: Editorial Board
        iii. Term Limit: 4 years
        iv. First year is always as an Assistant Editor, which counts towards the 4-year term limit. The second year is as an Editor, and the third and/or fourth year(s) as a Senior Editor.
        v. Any year(s) on the targeted SR team count towards 4-year term limit
    e. Technical Editor
        i. Eligibility: preference to prior reviewer experience
        Voting: Appointed by the Editor-in-Chief
        iii. Term Limit: 3 years
    f. Assistant Technical Editor
        i. Eligibility: This role is usually filled by a student in health sciences

ii. Voting: Appointed by the Editor-in-Chief

iii. Term Limit: 3 years Systematic Review (SR)

a. Targeted SR Lead (first author)

i. Eligibility: Current or previous Assistant SR Lead or one or more years as a reviewer and/or main review editor (previous: only if current advisor)

ii. Voting: Editorial Board + SR team (candidates abstain)

iii. Required to take the Cochrane SR Course (or similar) if not previously completed (namely, if not promoted from Assistant Targeted SR Lead)

iv. Term Limit: 1 year

b. Assistant Targeted SR Lead

i. Eligibility: Current or previous SR Team Member (previous: only if current advisor)

ii. Voting: Editorial Board + SR team (candidates abstain)

iii. Required to take the Cochrane SR Course (or similar)

iv. Term Limit:1 year

c. Targeted SR Advisor

i. Eligibility: Current or previous SR lead (previous: only if current advisor)

ii. Voting: Editorial Board + SR team (candidates abstain)

iii. Term Limit: 1 year

d. Targeted SR Team Member

i. Eligibility: Current or previous Editor (previous: only if current advisor) and current senior reviewers

ii. Voting: Editorial Board + current SR team (candidates abstain)

iii. Term Limit: 4 years

iv. Any year(s) as an editor on the main review (including assistant editor, editor, or senior editor) count towards 4-year term limit

v. SR Team Members are not members of the Editorial Board

3. Advisors

a. This is not an author level position, but will be recognized as member of the GEMLR Group similar to Reviewers

b. All author-level positions (EIC, ME, AE, Editor, and SR Lead or Team Member) in good standing are eligible to become an advisor on completion of term in any position

c. Advisors are required to take leadership positions in the different GEMLR committees as assigned each year by the EIC

d. Advisors are expected to participate in board meetings and votes

e. No term limit

Exceptions to term limits

a) If GEMLR is unable to recruit enough board members, the term of the most junior board members(s) will be extended by one year. If there is only one position that needs to be extended beyond the regular term, but more than one editor eligible for this extension, the executive group consisting of the editor-in-chief, managing editor, assistant managing editor and technical editor will decide which editor's term will be extended, taking editor interest and past performance into consideration.

b) Exceptions to this policy due to unforeseen or special circumstances may be made by simple majority vote of the Editorial Board.

Editor evaluation and termination

a) After the H1 and H2 phase, the Technical Editor will collect anonymous feedback on the Editors'/SR Lead's performance from the reviewers/team members. Submission of feedback will be mandatory. The Technical Editor will compile the feedback and share it with the Editor-in-Chief, the Managing Editor, and the Assistant Managing Editor who will review the feedback and share it with editors when appropriate and indicated, *ensuring that the confidentiality of reviewers providing the feedback is maintained.*

b) The Managing Editor will monitor the Editors'/SR Team Members' compliance with the GEMLR procedures as described in the Procedures Manual as well as with regards to meeting the established deadlines. Editors/SR Team Members who receive unfavorable reviewer feedback or fail to meet the GEMLR procedures or deadlines (as defined by submission more than one day late) will be issued a warning via email by the Editor-in-Chief.

c) Editors/SR Team Members are required to reply to the warning letter within 5 days. In their reply, they should explain what steps will be taken to avoid any future occurrences of this problem. If the Editor/SR Team Member fails or refuses to respond to the complaint, she/he will be terminated from the position immediately. Any Editor/SR Team Member who has received two warning letters will be terminated from her/his position immediately upon receipt of an additional (third) complaint.

d) After the H1 and H2 phase, the Editor-in-Chief will collect feedback from the committee chairs regarding each Editor and Advisor's contribution to the committee's work throughout the year. Editors, SR Team Members and Advisors not fulfilling their duties on their assigned committee will receive a warning letter.

e) At the discretion of the Editor-in-Chief and Managing Editor, Editors, SR Team Members and Advisors who receive any warning letters during the year may not be invited to return for the following year.

This policy went into effect on July 2021. Term limits for all positions covered under the previous (2016) policy will have years already accumulated included. For new positions/positions not previously covered by the 2016, the 2021-22 GEMLR cycle will count as the first year for the above-mentioned term limits.
