# Supplementary material for: Global Emergency Medicine: A Scoping Review of the Literature From 2024
Source: Acad Emerg Med. 2025 Dec 23;33(3):e70208. doi: 10.1111/acem.70208 (PMC12925323; doi:10.1111/acem.70208)
Supplement: Supplementary file 3 — Data S3: acem70208‐sup‐0003‐Supinfo2.pdf. [file ACEM-33-0-s002.pdf]

GEMLR Search String 2024

("Emergency Medicine"[Mesh] OR "Disaster Medicine"[Mesh] OR "Emergency Responders"[Mesh] OR "Emergency Treatment"[Mesh] OR "Emergency Medical Services"[Mesh] OR "ambulances"[MeSH] OR "critical care"[MeSH] OR "emergency respon*"[tw] OR "emergency doctor*"[tw] OR "emergency clinician*"[tw] OR "emergency physician*"[tw] OR "emergency personnel"[tw] OR "emergency medical personnel"[tw] OR "emergency service*"[tw] OR "emergency medical service*"[tw] OR "emergency medicine"[tw] OR "emergency health service*"[tw] OR "emergency care"[tw] OR "emergency health care"[tw] OR "emergency treatment*"[tw] OR "emergency department*"[tw] OR "emergency room*"[tw] OR "emergency ward*"[tw] OR "emergency units*"[tw] OR "emergency hospital*"[tw] OR "emergency clinic*"[tw] OR "emergency setting*"[tw] OR "emergency staff*"[tw] OR "emergency response*"[tw] OR "emergency medical technician"[tw] OR "paramedic*"[tw] OR "ambulance*"[tw] OR "ER"[tw] OR "first responder*"[tw] OR "rescue work*"[tw] OR "relief work*"[tw] OR "firefighter*"[tw] OR "fire fighter*"[tw] OR "trauma center*"[tw] OR "trauma unit*"[tw] OR "critical care"[tw] OR Disasters[Mesh] OR "Volcanic Eruptions"[Mesh] OR "radioactive hazard release"[MeSH] OR "warfare and armed conflicts"[MeSH] OR "terrorism"[MeSH] OR "refugees"[MeSH] OR "refugee camps"[MeSH] OR "disaster*"[tw] OR "avalanche*"[tw] OR "blizzard*"[tw] OR "cyclone*"[tw] OR "cyclonic storm*"[tw] OR "drought*"[tw] OR "earthquake*"[tw] OR "famine*"[tw] OR "flood*"[tw] OR "flooding"[tw] OR "hurricane*"[tw] OR "landslide*"[tw] OR "land slide*"[tw] OR "mudslide*"[tw] OR "mud slide*"[tw] OR "rockslide*"[tw] OR "rock slide*"[tw] OR "tidalwave*"[tw] OR "tidal wave*"[tw] OR "tsunami*"[tw] OR "tornado*"[tw] OR "tropical storm*"[tw] OR "typhoon*"[tw] OR "wildfire*"[tw] OR "wild fire*"[tw] OR "forest fire*"[tw] OR "volcanic eruption*"[tw] OR "war"[tw] OR "warfare"[tw] OR "armed conflict*"[tw] OR "terrorist*"[tw] OR "terrorism"[tw] OR "refugee*"[tw] OR "asylum seek*"[tw] OR "displaced person*"[tw] OR "displaced famil*"[tw] OR "humanitarian*"[tw] OR "mass casualt*"[tw] OR "mass shooting*"[tw] OR "conflict"[tw] OR "conflict-affected"[tw] OR "post-conflict"[tw] OR "wars"[tw] OR "pregnancy complications"[MeSH] OR "pregnancy complication*"[tw] OR "obstetric complication*"[tw] OR "obstetric emergenc*"[tw] OR "fetal death"[tw] OR "nuchal cord"[tw] OR "fetal hypoxia"[tw] OR "HELLP Syndrome"[tw] OR "eclampsia"[tw] OR "pre-eclamp*"[tw] OR "placental abruption"[tw] OR "dystocia"[tw] OR "premature rupture"[tw] OR "postpartum hemorrhage"[tw] OR "uterine rupture"[tw] OR "puerperal Infection"[tw] OR "tubal pregnancy"[tw] OR "ectopic pregnancy"[tw] OR "post-partum hemorrhage"[tw] OR "postpartum haemorrhage"[tw] OR "post-partum haemorrhage"[tw] OR "Disease Outbreaks"[Mesh] OR "Hemorrhagic Fever, Ebola"[Mesh] OR "Cholera"[Mesh] OR "measles"[MeSH:noexp] OR "Starvation"[Mesh] OR "shock"[MeSH] OR "sepsis"[MeSH] OR "wounds and injuries"[MeSH] OR "disease outbreak"[tw] OR "epidemic*"[tw] OR "hemorrhagic fever"[tw] OR "haemorrhagic fever"[tw] OR "ebola"[tw] OR "cholera"[tw] OR "measles"[tw] OR "starvation"[tw] OR "nuclear accident"[tw] OR "radiation accident"[tw] OR "critical illness*"[tw] OR "resuscitation"[tw] OR "shock"[tw] OR "sepsis"[tw] OR "septicemia"[tw] OR "septicaemia"[tw] OR "acute care"[tw] OR "acute disease"[tw] OR "prehospital"[tw] OR "pre-hospital"[tw] OR "wound*"[tw] OR "triage*"[tw] OR "CPR"[tw] OR "cardiopulmonary resuscitation"[tw]) AND (Developing Countries[MeSH] OR Africa[MeSH] OR Asia[MeSH:noexp] OR Asia, Central[MeSH] OR Asia, Southeastern[MeSH] OR Asia, Western[MeSH] OR Caribbean Region[MeSH] OR South America[MeSH] OR Latin America[MeSH] OR Central America[MeSH] OR Afghanistan[MeSH] OR Albania[MeSH] OR Algeria[MeSH] OR American Samoa[MeSH] OR Angola[MeSH] OR "Antigua and Barbuda"[MeSH] OR Argentina[MeSH] OR Armenia[MeSH] OR Azerbaijan[MeSH] OR Bahrain[MeSH] OR Bangladesh[MeSH] OR Barbados[MeSH] OR Benin[MeSH] OR Byelarus[MeSH] OR Belize[MeSH] OR Bhutan[MeSH] OR Bolivia[MeSH] OR Bosnia-Herzegovina[MeSH] OR Botswana[MeSH] OR Brazil[MeSH] OR Bulgaria[MeSH] OR Burkina Faso[MeSH] OR Burundi[MeSH] OR Cambodia[MeSH] OR Cameroon[MeSH] OR Cape Verde[MeSH] OR Central African Republic[MeSH] OR Chad[MeSH] OR Chile[MeSH] OR China[MeSH] OR Colombia[MeSH] OR Comoros[MeSH] OR Congo[MeSH] OR Costa Rica[MeSH] OR Cote d'Ivoire[MeSH] OR Croatia[MeSH] OR Cuba[MeSH] OR Cyprus[MeSH] OR Czechoslovakia[MeSH] OR Czech Republic[MeSH] OR Slovakia[MeSH] OR Djibouti[MeSH] OR "Democratic Republic of the Congo"[MeSH] OR Dominica[MeSH] OR Dominican Republic[MeSH] OR East Timor[MeSH] OR Ecuador[MeSH] OR Egypt[MeSH] OR El Salvador[MeSH] OR Eritrea[MeSH] OR Estonia[MeSH] OR Ethiopia[MeSH] OR Fiji[MeSH] OR Gabon[MeSH] OR Gambia[MeSH] OR "Georgia (Republic)"[MeSH] OR Ghana[MeSH] OR Greece[MeSH] OR Grenada[MeSH] OR Guatemala[MeSH] OR Guinea[MeSH] OR Guinea-Bissau[MeSH] OR Guam[MeSH] OR Guyana[MeSH] OR Haiti[MeSH] OR Honduras[MeSH] OR Hungary[MeSH] OR India[MeSH] OR Indonesia[MeSH] OR Iran[MeSH] OR Iraq[MeSH] OR Jamaica[MeSH] OR Jordan[MeSH] OR Kazakhstan[MeSH] OR Kenya[MeSH] OR Korea[MeSH] OR

Kosovo[MeSH] OR Kyrgyzstan[MeSH] OR Laos[MeSH] OR Latvia[MeSH] OR Lebanon[MeSH] OR Lesotho[MeSH] OR Liberia[MeSH] OR Libya[MeSH] OR Lithuania[MeSH] OR Macedonia[MeSH] OR Madagascar[MeSH] OR Malaysia[MeSH] OR Malawi[MeSH] OR Mali[MeSH] OR Malta[MeSH] OR Mauritania[MeSH] OR Mauritius[MeSH] OR Mexico[MeSH] OR Micronesia[MeSH] OR Middle East[MeSH] OR Moldova[MeSH] OR Mongolia[MeSH] OR Montenegro[MeSH] OR Morocco[MeSH] OR Mozambique[MeSH] OR Myanmar[MeSH] OR Namibia[MeSH] OR Nepal[MeSH] OR Netherlands Antilles[MeSH] OR New Caledonia[MeSH] OR Nicaragua[MeSH] OR Niger[MeSH] OR Nigeria[MeSH] OR Oman[MeSH] OR Pakistan[MeSH] OR Palau[MeSH] OR Panama[MeSH] OR Papua New Guinea[MeSH] OR Paraguay[MeSH] OR Peru[MeSH] OR Philippines[MeSH] OR Poland[MeSH] OR Portugal[MeSH] OR Puerto Rico[MeSH] OR Romania[MeSH] OR Russia[MeSH] OR "Russia (Pre-1917)"[MeSH] OR Rwanda[MeSH] OR "Saint Kitts and Nevis"[MeSH] OR Saint Lucia[MeSH] OR "Saint Vincent and the Grenadines"[MeSH] OR Samoa[MeSH] OR Saudi Arabia[MeSH] OR Senegal[MeSH] OR Serbia[MeSH] OR Montenegro[MeSH] OR Seychelles[MeSH] OR Sierra Leone[MeSH] OR Slovenia[MeSH] OR Sri Lanka[MeSH] OR Somalia[MeSH] OR South Africa[MeSH] OR Sudan[MeSH] OR Suriname[MeSH] OR Swaziland[MeSH] OR Syria[MeSH] OR Tajikistan[MeSH] OR Tanzania[MeSH] OR Thailand[MeSH] OR Togo[MeSH] OR Tonga[MeSH] OR "Trinidad and Tobago"[MeSH] OR Tunisia[MeSH] OR Turkey[MeSH] OR Turkmenistan[MeSH] OR Uganda[MeSH] OR Ukraine[MeSH] OR Uruguay[MeSH] OR USSR[MeSH] OR Uzbekistan[MeSH] OR Vanuatu[MeSH] OR Venezuela[MeSH] OR Vietnam[MeSH] OR Yemen[MeSH] OR Yugoslavia[MeSH] OR Zambia[MeSH] OR Zimbabwe[MeSH] OR "developing country"[tw] OR "developing countries"[tw] OR "developing nation"[tw] OR "developing nations"[tw] OR "developing population"[tw] OR "developing populations"[tw] OR "developing world"[tw] OR "less developed country"[tw] OR "less developed countries"[tw] OR "less developed nation"[tw] OR "less developed nations"[tw] OR "less developed population"[tw] OR "less developed populations"[tw] OR "less developed world"[tw] OR "lesser developed country"[tw] OR "lesser developed countries"[tw] OR "lesser developed nation"[tw] OR "lesser developed nations"[tw] OR "lesser developed population"[tw] OR "lesser developed populations"[tw] OR "lesser developed world"[tw] OR "least developed country"[tw] OR "least developed countries"[tw] OR "least developed nation"[tw] OR "least developed nations"[tw] OR "least developed population"[tw] OR "least developed populations"[tw] OR "least developed world"[tw] OR "under developed country"[tw] OR "under developed countries"[tw] OR "under developed nation"[tw] OR "under developed nations"[tw] OR "under developed population"[tw] OR "under developed populations"[tw] OR "under developed world"[tw] OR "underdeveloped country"[tw] OR "underdeveloped countries"[tw] OR "underdeveloped nation"[tw] OR "underdeveloped nations"[tw] OR "underdeveloped population"[tw] OR "underdeveloped populations"[tw] OR "underdeveloped world"[tw] OR "middle income country"[tw] OR "middle income countries"[tw] OR "middle income nation"[tw] OR "middle income nations"[tw] OR "middle income population"[tw] OR "middle income populations"[tw] OR "low income country"[tw] OR "low income countries"[tw] OR "low income nation"[tw] OR "low income nations"[tw] OR "low income population"[tw] OR "low income populations"[tw] OR "lower income country"[tw] OR "lower income countries"[tw] OR "lower income nation"[tw] OR "lower income population"[tw] OR "underserved country"[tw] OR "underserved countries"[tw] OR "underserved nation"[tw] OR "underserved nations"[tw] OR "underserved population"[tw] OR "underserved populations"[tw] OR "underserved world"[tw] OR "under served country"[tw] OR "under served countries"[tw] OR "under served nation"[tw] OR "under served nations"[tw] OR "under served population"[tw] OR "under served populations"[tw] OR "under served world"[tw] OR "deprived country"[tw] OR "deprived countries"[tw] OR "deprived nation"[tw] OR "deprived nations"[tw] OR "deprived population"[tw] OR "deprived world"[tw] OR "poor country"[tw] OR "poor countries"[tw] OR "poor nation"[tw] OR "poor nations"[tw] OR "poor populations"[tw] OR "poor world"[tw] OR "poorer country"[tw] OR "poorer countries"[tw] OR "poorer nation"[tw] OR "poorer nations"[tw] OR "poorer population"[tw] OR "poorer populations"[tw] OR "poorer world"[tw] OR "developing economy"[tw] OR "developing economies"[tw] OR "less developed economy"[tw] OR "less developed economies"[tw] OR "lesser developed economy"[tw] OR "lesser developed economies"[tw] OR "under developed economy"[tw] OR "under developed economies"[tw] OR "underdeveloped economy"[tw] OR "underdeveloped economies"[tw] OR "middle income economy"[tw] OR "middle income economies"[tw] OR "low income economy"[tw] OR "low income economies"[tw] OR "lower income economy"[tw] OR "lower income economies"[tw] OR "low gdp"[tw] OR "low gnp"[tw] OR "low gross domestic"[tw] OR "low gross national"[tw] OR "lower gdp"[tw] OR "lower gnp"[tw] OR "lower gross domestic"[tw] OR "lower gross national"[tw] OR lmic[tw] OR lmics[tw] OR "third world"[tw] OR "lami country"[tw] OR "lami countries"[tw] OR "transitional country"[tw] OR "transitional countries"[tw] OR Africa[tw] OR Asia[tw] OR Caribbean[tw] OR West Indies[tw] OR South America[tw] OR Latin America[tw] OR Central America[tw] OR Afghanistan[tw] OR Albania[tw] OR Algeria[tw] OR Angola[tw] OR

Antigua[tw] OR Barbuda[tw] OR Argentina[tw] OR Armenia[tw] OR Armenian[tw] OR Aruba[tw] OR Azerbaijan[tw] OR Bahrain[tw] OR Bangladesh[tw] OR Barbados[tw] OR Benin[tw] OR Byelarus[tw] OR Byelorussian[tw] OR Belarus[tw] OR Belorussian[tw] OR Belorussia[tw] OR Belize[tw] OR Bhutan[tw] OR Bolivia[tw] OR Bosnia[tw] OR Herzegovina[tw] OR Hercegovina[tw] OR Botswana[tw] OR Brasil[tw] OR Brazil[tw] OR Bulgaria[tw] OR Burkina Faso[tw] OR Burkina Fasso[tw] OR Upper Volta[tw] OR Burundi[tw] OR Urundi[tw] OR Cambodia[tw] OR Khmer Republic[tw] OR Kampuchea[tw] OR Cameroon[tw] OR Cameroons[tw] OR Cameron[tw] OR Cape Verde[tw] OR Central African Republic[tw] OR Chad[tw] OR China[tw] OR Colombia[tw] OR Comoros[tw] OR Comoro Islands[tw] OR Comores[tw] OR Mayotte[tw] OR Congo[tw] OR Zaire[tw] OR Costa Rica[tw] OR Cote d'Ivoire[tw] OR Ivory Coast[tw] OR Croatia[tw] OR Cuba[tw] OR Cyprus[tw] OR Czechoslovakia[tw] OR Czech Republic[tw] OR Slovakia[tw] OR Slovak Republic[tw] OR Djibouti[tw] OR French Somaliland[tw] OR Dominica[tw] OR Dominican Republic[tw] OR East Timor[tw] OR East Timur[tw] OR Timor Leste[tw] OR Ecuador[tw] OR Egypt[tw] OR United Arab Republic[tw] OR El Salvador[tw] OR Eritrea[tw] OR Estonia[tw] OR Ethiopia[tw] OR Fiji[tw] OR Gabon[tw] OR Gabonese Republic[tw] OR Gambia[tw] OR Gaza[tw] OR Georgia Republic[tw] OR Georgian Republic[tw] OR Ghana[tw] OR Gold Coast[tw] OR Greece[tw] OR Grenada[tw] OR Guatemala[tw] OR Guinea[tw] OR Guam[tw] OR Guiana[tw] OR Guyana[tw] OR Haiti[tw] OR Honduras[tw] OR Hungary[tw] OR India[tw] OR Maldives[tw] OR Indonesia[tw] OR Iran[tw] OR Iraq[tw] OR Isle of Man[tw] OR Jamaica[tw] OR Jordan[tw] OR Kazakhstan[tw] OR Kazakh[tw] OR Kenya[tw] OR Kiribati[tw] OR Korea[tw] OR Kosovo[tw] OR Kyrgyzstan[tw] OR Kirghizia[tw] OR Kyrgyz Republic[tw] OR Kirghiz[tw] OR Kirgizstan[tw] OR "Lao PDR"[tw] OR Laos[tw] OR Latvia[tw] OR Lebanon[tw] OR Lesotho[tw] OR Basutoland[tw] OR Liberia[tw] OR Libya[tw] OR Lithuania[tw] OR Macedonia[tw] OR Madagascar[tw] OR Malagasy Republic[tw] OR "Malaysia*"[tw] OR Malaya[tw] OR Malay[tw] OR Sabah[tw] OR Sarawak[tw] OR Malawi[tw] OR Nyasaland[tw] OR Mali[tw] OR Malta[tw] OR Marshall Islands[tw] OR Mauritania[tw] OR Mauritius[tw] OR Agalega Islands[tw] OR Mexico[tw] OR Micronesia[tw] OR Middle East[tw] OR Moldova[tw] OR Moldovia[tw] OR Moldovian[tw] OR Mongolia[tw] OR Montenegro[tw] OR Morocco[tw] OR Ifni[tw] OR Mozambique[tw] OR Myanmar[tw] OR Myanma[tw] OR Burma[tw] OR Namibia[tw] OR Nepal[tw] OR Netherlands Antilles[tw] OR New Caledonia[tw] OR Nicaragua[tw] OR Niger[tw] OR Nigeria[tw] OR Northern Mariana Islands[tw] OR Oman[tw] OR Muscat[tw] OR Pakistan[tw] OR Palau[tw] OR Palestine[tw] OR Panama[tw] OR Paraguay[tw] OR Peru[tw] OR Philippines[tw] OR Philipines[tw] OR Phillipines[tw] OR Phillippines[tw] OR Poland[tw] OR Portugal[tw] OR Puerto Rico[tw] OR Rhodesia[tw] OR Romania[tw] OR Rumania[tw] OR Roumania[tw] OR Russia[tw] OR Russian[tw] OR Rwanda[tw] OR Ruanda[tw] OR Saint Kitts[tw] OR St Kitts[tw] OR Nevis[tw] OR Saint Lucia[tw] OR St Lucia[tw] OR Saint Vincent[tw] OR St Vincent[tw] OR Grenadines[tw] OR Samoa[tw] OR Samoan Islands[tw] OR Navigator Island[tw] OR Navigator Islands[tw] OR Sao Tome[tw] OR Saudi Arabia[tw] OR Senegal[tw] OR Serbia[tw] OR Montenegro[tw] OR Seychelles[tw] OR Sierra Leone[tw] OR Slovenia[tw] OR Sri Lanka[tw] OR Ceylon[tw] OR Solomon Islands[tw] OR Somalia[tw] OR Sudan[tw] OR Suriname[tw] OR Surinam[tw] OR Swaziland[tw] OR Syria[tw] OR Tajikistan[tw] OR Tadzhikistan[tw] OR Tadjikistan[tw] OR Tadzhik[tw] OR Tanzania[tw] OR Thailand[tw] OR Togo[tw] OR Togolese Republic[tw] OR Tonga[tw] OR Trinidad[tw] OR Tobago[tw] OR Tunisia[tw] OR Turkey[tw] OR Turkmenistan[tw] OR Turkmen[tw] OR Uganda[tw] OR Ukraine[tw] OR Uruguay[tw] OR USSR[tw] OR Soviet Union[tw] OR Union of Soviet Socialist Republics[tw] OR Uzbekistan[tw] OR Uzbek[tw] OR Vanuatu[tw] OR New Hebrides[tw] OR Venezuela[tw] OR Vietnam[tw] OR Viet Nam[tw] OR West Bank[tw] OR Yemen[tw] OR Yugoslavia[tw] OR Zambia[tw] OR Zimbabwe[tw] OR "world health"[tw] OR "international"[tw] OR "global"[tw] OR "worldwide"[tw] OR "resource limited setting*"[tw] OR "resource-limited setting*"[tw] OR "limited resource setting*"[tw] OR "limited-resource setting*"[tw] OR "resource limited context*"[tw] OR "resource-limited context*"[tw] OR "limited resource context*"[tw] OR "limited-resource context*"[tw] OR "conflict-affected"[tw] OR "post-conflict"[tw] OR "DRC"[tw]) OR "Afr J Emerg Med"[jour] OR "Bull World Health Organ"[jour] OR "Lancet"[jour] OR "Prehosp Disaster Med"[jour] NOT (News[ptyp] OR English Abstract[ptyp] OR Preprint[ptyp] OR Clinical Trial Protocol[ptyp] OR Case Reports[ptyp] OR Editorial[ptyp] OR Letter[ptyp] OR Newspaper Article[ptyp])
