# Supplementary material for: Global Emergency Medicine: A Scoping Review of the Literature From 2024
Source: Acad Emerg Med. 2025 Dec 23;33(3):e70208. doi: 10.1111/acem.70208 (PMC12925323; doi:10.1111/acem.70208)
Supplement: Supplementary file 4 — Data S4: acem70208‐sup‐0004‐Supinfo3.pdf. [file ACEM-33-0-s007.pdf]

<u>Scoring of Original Research Article</u>

Quality Measure	Question		Points
Design	*<u>Select One</u>*	Descriptive studies (including case studies and case series, natural observation studies and descriptive surveys) including qualitative studies which only report anecdotal statements without any further analysis	1 *-or-*
		Correlation studies (case control studies, prospective observational studies, retrospective studies) **or** qualitative studies that either coded responses or used a standardized survey/interview tool	2 *-or-*
		Non-randomized or non-blinded experimental studies **or** qualitative studies that both coded responses and used a standardized survey/interview tool	3 *-or-*
		Randomized, blinded experimental studies **or** qualitative studies using a validated, standardized survey or interview structure, systematic and reproducible coding and analysis with appropriate reporting of identified themes	4
	Correct statistical tests are used to analyze quantitative data. For qualitative data analysis, involves coding that identifies emerging themes and appropriate reduction and interpretation of data (for example, into appropriate diagrams, matrices or taxonomies)		__/1
	No obvious bias in the selection of the subjects or authors attempt to limit bias		__/1
Design Total			/ Out of max score 6
Ethics	Contribution from authors with a primary affiliation in the country(ies) that the research was either performed in or is specifically relevant to, and that author is the first and/or last author		__/1
	The study was approved by a local or international IRB, a government ministry, or a community group and the study clearly adheres to the Declaration of Helsinki (see Appendix 1)*		__/1
	Either written or verbal consent was obtained in the subject's own language or consent was waived by IRB		__/1
	The authors declare that they have no significant conflicts of interest.		__/1
Ethics Total			/ Out of a max of 4

<table>
<tr><td rowspan="3">Importance</td><td>The study results are not specific to one area or situation but are broadly generalizable to a variety of settings:
● at the regional/single-country level (1)
● at an international level (+1 = total of 2 points if important internationally)</td><td>__/2</td></tr>
<tr><td>The topic being studied is an important one, in that it affects…:
● many people (1)
● especially vulnerable groups as defined at www.un.org/en/fight-racism/vulnerable-groups (1)</td><td>__/2</td></tr>
<tr><td>The study is clearly relevant to the realm of global emergency medicine.</td><td>__/1</td></tr>
<tr><td>Importance Total</td><td colspan="2">/ Out of a max of 5</td></tr>
<tr><td rowspan="4">Impact</td><td>The study describes an intervention that may be feasibly implemented by practitioners in developing countries.</td><td>__/1</td></tr>
<tr><td>The article considers cost-effectiveness (A formal cost-effectiveness analysis is not required, however authors must demonstrate an understanding of the financial costs of implementing their research findings in their practice area)</td><td>__/1</td></tr>
<tr><td>The results would lead to a change in policy:
● at the regional/single-country level (1)
● at an international level (1) (+1 = total of 2 points if important internationally)</td><td>__/2</td></tr>
<tr><td>The results of the research are likely to result in improved health of the affected population</td><td>__/1</td></tr>
<tr><td>Impact Total</td><td colspan="2">/ Out of a max of 5</td></tr>
</table>

* Do not automatically award credit for IRB approval to research using publicly available, de-identified databases. Authors must still report IRB approval or exemption from IRB approval in the manuscript.

Scoring of Review (RE) Articles

Quality Measure	Question	Points
**Clarity**	The review has a clearly stated hypothesis or purpose.	__/2
	The authors provide sufficient background to put the results of the review into context.	__/1
	The review can be understood by someone with general medical or public health training.	__/1
	The authors use clear language and appropriate graphs, tables, and figures throughout the article.	__/1
Clarity Total	/ Out of max score 5	
**Design**	This is a formal meta-analysis or a systematic review that only includes studies with a control group.	__/2
	There is a clear, reproducible method for the selection of studies included in this review.	__/1
	Articles for this review were selected by at least two authors blinded to each other's selection.	__/1
	The data was aggregated and/or analyzed appropriately.	__/1
	Contribution from authors with a primary affiliation in the country(ies) that the research was either performed in or is specifically relevant to, and that author is the first and/or last author	__/1
Design Total	/ Out of max score 6	
**Importance**	The study results are not specific to one area or situation but are broadly generalizable to a variety of settings:<br>● at the regional/single-country level (1)<br>● at an international level (1) (+1 = total of 2 points if important internationally)	__/2
	The topic being studied is an important one, in that it affects…:<br>● many people (1)<br>● especially vulnerable groups as defined at www.un.org/en/fight-racism/vulnerable-groups (1)	__/2
	This is clearly relevant to the realm of Global Emergency Medicine.	__/1
Importance Total	/ Out of max score 5	

**Impact**	The results would lead to a change in policy:<br>● at the regional/single-country level (1)<br>● at an international level (1) (+1 = total of 2 points if important internationally)	__/2
	The article considers cost-effectiveness (A formal cost-effectiveness analysis is not required, however authors must demonstrate an understanding of the financial costs of implementing their research findings in their practice area)	__/1
	The results of the research are likely to result in improved health of the affected population	__/1
Impact Total	/ Out of max score 4	

Scoring of Gray Literature (GR) Articles

Quality Measure	Question	Points
**Design**	The article relies upon:<br>● expert consensus (1)<br>● review of existing literature (1)<br>● primary data (1) (= total of 3 points if all three)	__/3
**Design Total**	**/ Out of max score 3**	
**Importance**	The study conclusions or recommendations are not specific to one area or situation but are broadly generalizable to a variety of settings:<br>● at the regional/single-country level (1)<br>● at an international level (1) (+1 = total of 2 points if important internationally)	__/2
	The topic being studied is an important one, in that it affects…:<br>● many people (1)<br>● especially vulnerable groups* (1) (total of 2 points if both)<br>*defined at un.org/en/fight-racism/vulnerable-groups	__/2
	The article is clearly relevant to the realm of Global Emergency Medicine	__/1
**Importance Total**	**/ Out of max score 5**	
**Impact**	The results would lead to a change in policy:<br>● at the local/national level (1),<br>● at an international level (1) (+1=total of 2 points if important internationally)<br>Or:<br>● recommendations or interventions suggested in the article may be feasibly implemented by practitioners in developing countries (1) (1 point total)	__/2
	The article considers cost-effectiveness (A formal cost-effectiveness analysis is not required, however authors must demonstrate an understanding of the financial costs of implementing their research findings in their practice area)	__/1
	Adoption of the article's recommendations are likely to result in improved health of the affected population.	__/1
**Impact Total**	**/ Out of max score 4**	
