# Supplementary material for: Global Emergency Medicine: A Scoping Review of the Literature From 2024
Source: Acad Emerg Med. 2025 Dec 23;33(3):e70208. doi: 10.1111/acem.70208 (PMC12925323; doi:10.1111/acem.70208)
Supplement: Supplementary file 5 — Data S5: acem70208‐sup‐0005‐Supinfo4.pdf. [file ACEM-33-0-s001.pdf]

First Name	Last Name	Year, from 2005-2024																				Affiliation / Country	LMIC Yes	Other HIC	Years Review	Years Author	Years Advisor	Service Years	Targeted SR
		05	06	07	08	09	10	11	12	13	14	15	16	17	18	19	20	21	22	23	24								
Nada	A-Rahman									R												Omoduraman, Sudan	Y		1			1	
Abdirahman	Abdulle																				R	Kampala, Uganda	Y		1			1	
Zainab	Ahmed																		R	R		Los Angeles, CA, USA			2			2	
Peter	Aitken							R	R													Queensland, Australia		Y	2			2	
Halley J	Alberts																				R	Columbia SC, USA			1			1	
R Elanor	Anderson										R	R	R	R	R							Boston, MA; New Haven CT, USA			5			5	
David	Anthony						R															New York, NY, USA			1			1	
Maya	Arii				R																	New Haven, CT, USA			1			1	
Paige	Armstrong											R										Washington, DC, USA			1			1	
Kris	Arnold	Au	Au	Au	Au	Au	Au	Ad	Ad	Ad												Boston, MA, USA			0	6	3	9	
Lisa	Arvold					R																Boston, MA, USA			1			1	
Miriam	Aschkenasy					Au	Au	Au	Ad	Ad	Ad	Ad										Cambridge, MA, USA			0	3	4	7	
Kimonia Bih	Awanchiri																			R	R	Cameroon	Y		2			2	
Kamna S	Balhara									R	R	R	R	R	Au	Au	Au	Ad	Ad			Baltimore, MD; San Antonio TX, USA			5	3	2	10	2
N. Shakira	Bandolin															R	R	R	Au	Au		Sacramento, CA, USA			3	2		5	
Holly	Bannon-Murphy														R	R	R	R				Melbourne, Australia		Y	4			4	
Susan	Bartels							R	Au	Au	Au	Au	Au	Ad	Ad							Boston, MA, USA; Kingston Ontaria Canada		Y	1	5	2	8	
Emily	Bartlett																			R	R	Albuquerque, NM, USA			2			2	
Joseph	Becker		Au	Au	Au		Ad	Ad														Palo Alto, CA, USA			0	3	2	5	
Torben Kim	Becker					R	R	Au	Au	Au	Au	Au	Au	Au	Au	Au	Au	Au	Ad			Heiderlberg, Germany; Ann Arbor MI; Gainsville FL, USA		Y	2	11	1	14	5
Suzanne	Bentley				R																	New York, NY, USA			1			1	
Tal	Berkowitz																			R	R	Atlanta, GA, USA			2			2	
Karina	Bertsch						Au	Au														Providence, RI, USA			0	2		2	
Temesgen	Beyene												R	R	R							Addis Ababa, Ethiopia	Y		3			3	
Nidhi	Bhaskar															Au	Au	Au	Au	Ad	Ad	Providence, RI, USA			0	4	2	6	
Corey	Bills												R	R				R	R	R	Ad	San Franscico, CA; Aurora CO, USA			5		1	6	5
Mark	Bisanzo							R	R	R	R	Au										Worcester, MA, USA			4	1		5	
Aislinn	Black							R	R	R	R											Stony Brook, NY; Baltimore MD, USA			4			4	
Michael	Blake			R	R																	Washington, DC, USA			2			2	
Christina	Bloem												R									Brooklyn, NY, USA			1			1	
Joseph	Bonney												R	R	R	Au	Au	Au	Au	Au	Ad	Kumasi, Ghana	Y		3	5	1	9	
Addie	Boone																R	R				Fort Worth TX, USA			2			2	
Nichole	Bosson			R	R	R																New York, NY, USA			3			3	
Michael	Boyd									R	R	R										Ann Arbor, MI, USA			3			3	
Morgan	Broccoli																R	R	R	Au	Au	Boston, MA, USA			3	2		5	
Whitney	Bryant			R																R	R	New York, NY; Cincinati, OH, USA			3			3	
Agatha	Brzezinksi																			R	R	Anchorage, AK, USA			2			2	
Nirma D	Bustamante												R	R								Boston, MA, USA			2			2	2
Juliana	Capatosto				R																	New York, NY, USA			1			1	
Jennifer	Chan					Au	Ad	Ad	Ad	Ad	Ad	Ad	Ad	Ad								Chicago, IL, USA			0	1	8	9	
Jonathan	Chan																			R	R	Roseville, CA, USA			2			2	
Julia	Chang												Au	Ad								Providence, RI, USA			0	1	1	2	
Andrew DI	Charlton															R	R	R				Bradford, UK		Y	3			3	
Chuan-Jay Jeffrey	Chen																	R	R			St. Helena, CA, USA			2			2	
Nina	Chicharoen		Au	Ad	Ad																	Brockton, MA, USA			0	1	2	3	
Emily	Chien																			R		New York, NY, USA			1			1	
Samah	Chiry									R												Montreal, Quebec, Canada		Y	1			1	

First Name	Last Name	Year, from 2005-2024																				Affiliation / Country	LMIC Yes	Other HIC	Years Review	Years Author	Years Advisor	Service Years	Targeted SR
		05	06	07	08	09	10	11	12	13	14	15	16	17	18	19	20	21	22	23	24								
Daniel	Cho													Au	Au	Ad	Ad	Ad				Providence, RI, USA			0	2	3	5	
Joseph	Ciano																R	R	R	R	Au	New Hyde Park, NY, USA			4	1		5	
Cassandra	Clay																	R	R	R	R	Gainsville, FL; Tulsa OK; Tahlequah OK, USA			4			4	
Emily	Cloessner																			R	R	St. Louis, MO, USA			2			2	
Hannah	Cockrell								Au	Au												Providence, RI, USA			0	2		2	
Amin	Coker												R	R		R						Kumasi, Ghana; Accra Ghana	Y		3			3	2
Amanda	Collier													R	R	R	R	Au	Au	Au	Au	Kingston, Ontario, Canada		Y	4	4		8	
Jolene	Cook												R	R	R	R						Halifax Nova Scotia, Canada		Y	4			4	
Emma	Cortes																		R	R		Philadelphia, PA, USA			2			2	
Anne	Daul				R																	Charlotte, NC, USA			1			1	
Nanaba A	Dawson-Amoah																	R	R	Au	Au	Accra, Ghana	Y		2	2		4	
Gabriel Luca	de Oliveria Salvador																	R	R	Au		Parana, Brazil	Y		2	1		3	
Robery Myles	Dickason											R										New York NY, USA			1			1	
Kamoga	Dickson																				R	Kampala, Uganda	Y		1			1	
Julia	Dixon																	R				Aurora, CO, USA			1			1	
Herbert	Duber						R	R	Au	Au												Boston, MA; Seattle WA, USA			2	2		4	
Jonathan W	Dyal														R	R	R	R	Au	Au		Boston, MA; Houston TX, USA			4	2		6	1
Karen	Ekernas										R											Minneapolis, MN, USA			1			1	
Christian	Engelen																				R	Pegnitz, Germany		Y	1			1	
Kayley	Enriquez														R							San Francisco, CA, USA			1			1	
Abiola	Fasina				R																	New York, NY, USA			1			1	
Katelyn	Flaherty															R	R		R			Gainsville, FL, USA			3			3	
Mark	Foran			R	Au	Au	Au	Au	Au	Au												Boston, MA, USA			1	6		7	
Jack	Forrest				R	R																Jacksonville, FL, USA			2			2	
Brandon	Friedman																				R	Charlotte NC, USA			1			1	
Silpa	Gadiraju	Au																				Boston, MA, USA			0	1		1	
Stephanie Chow	Garbern													R				R	R	R	Ad	Providence, RI, USA			4			4	5
Juliette	Gerardo																				R	Davis CA, USA			1			1	
Melaku	Getachew																	R				Harar, Ethiopia	Y		1			1	
Maria	Glenn				R																	Charlotte, NC, USA			1			1	
Paul	Glover												R									Accra, Ghana	Y		1			1	
Ashish	Goel	Au	Au	Ad	Ad																	Delhi, India	Y		0	2	2	4	
Elizabeth	Goldberg					R	R	Au	Au													Providence, RI, USA			2	2		4	
Danica	Gomez														R							Atlanta, GA, USA			1			1	
Emily	Grover										R	R	R									Boston, MA, USA			3			3	
Vineet	Gupta			R	R																	Delhi, India	Y		2			2	
Mindi	Guptill																				R	Loma Linda CA, USA			1			1	
Camilo E	Gutierrez													R								Boston MA, USA			1			1	
Reid	Haflich																		R	R		Seattle, WA; Anchorage AK, USA			2			2	1
Heather	Hammerstedt			R																		Boston, MA, USA			1			1	
Bhakti	Hansoti							R	R	R	Au	Au	Au	Ad								Baltimore, MD, USA			3	3	1	7	
Emily	Hartford															R	R					Seattle, WA, USA			2			2	
Mohammad Adrian	Hasdianda																	R				Boston, MA, USA			1			1	
Mohamed	Hassan																			R		Tanta, Egypt	Y		1			1	
Mark	Hauswald											R	R	R								Albuquerque, NM, USA			3			3	
Alison	Hayward									R	R	R	Au	Au	Ad	Ad	Ad	Ad	Ad	Ad	Ad	New Haven, CT; Providence RI, USA			3	2	7	12	
Braden	Hexom							R	R	R	R	R	Au	Au	Au	Au	Au	Au	Au	Au	Au	New York, NY; Chicago, IL, USA			5	9		14	

First Name	Last Name	Year, from 2005-2024																				Affiliation / Country	LMIC Yes	Other HIC	Years Review	Years Author	Years Advisor	Service Years	Targeted SR
		05	06	07	08	09	10	11	12	13	14	15	16	17	18	19	20	21	22	23	24								
Taylor	Hickey																				R	Pikeville, KY, USA			1			1	
Keanoo	Hill																			R		Houston, TX, USA			1			1	
Anneka	Hooft																			R	R	San Franscico, CA, USA			2			2	
Cheryl Lynn	Horton					R	R															Boston, MA, USA			2			2	
Emily	House											R	R									Kingston Ontario, Canada		Y	2			2	
Carolyn	Hunter															R	R	R	R			Glasgow, UK		Y	4			4	
Wesam MA	Ibrahim														R							Tanta, Egypt	Y		1			1	
Emmanuel Oluyinka	Idowu																				R	Nigeria	Y		1			1	
Rachel Anne	Inbanathan								R													Sydney, Australia		Y	1			1	
Ashley	Jacobson																R	R	R		Au	Rochester, MN; Eau Claire WI, USA			3	1		4	
Gabrielle	Jacquet					R	R	Au	Au	Au	Au	Au	Ad									Denver, CO, USA			2	5	1	8	
Joshua	Jauregui					R	R	R	R	R												Providence, RI; Seattle WA, USA			5			5	
Aqeel	Jawahir																				R	Charleston SC, USA			1			1	
Tomislav	Jelic				R	R																Manitoba, Canada		Y	2			2	
Alexander	Jenson										R	R	R									Baltimore, MD, USA			3			3	
Okechukwu Ogbonnda	Jibuuke								R	R												Manchester, UK		Y	2			2	
Sandeep	Johar	Au	Au		Ad	Ad	Ad															Rochester, NY; Jacksonville, FL; Hartford CT, USA			0	2	3	5	
Anjni	Joiner															R	R					Durham, NC, USA			2			2	
Jennifer	Jones															R	R	R	R	R	Au	Shreveport, LA; Palo Alto CA; Springfield MA, USA			5	1		6	
Aditi	Joshi					R	R															Jersey City, NJ, USA			2			2	
Vinay	Kampalath																R	R	R	Au	Au	Philadelphia, PA, USA			3	2		5	
Amanda	Kao							R														Denver, CO, USA			1			1	
Stephanie	Kayden							R	R	R	R											Cambridge, MA, USA			4			4	
Alexis	Kearney										R	R	R	R								Providence, RI, USA			4			4	
Elizabeth	Keating																			R	R	Salt Lake City UT, USA			2			2	
C. Ryan	Keay		Au	Ad	Ad																	Denver, CO, USA			0	1	2	3	
Devin Mansfield	Keefe											R	R	R	R							Baltimore MD; Plymouth NH, USA			4			4	
Andrew	Kestler									R	R											Vancouver, BC, Canada		Y	2			2	
Murdoc	Khaleghi		Au	Ad																		Springfield, MA, USA			0	1	1	2	
Renee	King	Au																				Syracuse, NY, USA			0	1		1	
Sampsa	Kiuru						R	R														Lappeenranta, Finland		Y	2			2	
Sean	Kivlehan											R	R	Au	Au	Au	Au	Au	Au	Au	Ad	Boston, MA, USA			2	7	1	10	5
Ioannis	Koutroulis												R									Philadelphia, PA, USA			1			1	
Katie	Koval												R	R								Stanford, CA, USA			2			2	
Arthi	Kozhumam																				R	Chicago IL, USA			1			1	
Vijaya Arun	Kumar																			R		Detroit, MI, USA			1			1	
Christopher	Lam										Au	Au	Ad									Providence, RI, USA			0	2	1	3	
Colleen	Laurence																R	R	R	R		Cincinati, OH; Boston MA, USA			4			4	1
Cappi	Lay		Au	Ad																		New York, NY, USA			0	1	1	2	
Joseph	Leanza															R	R	R	R	R	Au	Philadelphia, PA; Boston MA, USA			5	1		6	
Elizabeth	Ledger															R	R	R				Bristol, UK		Y	3			3	
J. Austin	Lee														R	R	R	R	Au	Au	Au	Charlottesville, VA; Providence RI; Bloomington, IN, USA			4	3		7	1
Sangil	Lee													R	R							Iowa City, IA, USA			2			2	
Adam	Levine	Au	Au	Au	Au	Au	Au	Au	Au	Au	Au	Au	Au	Au	Au	Ad	Ad	Ad				Boston, MA; Providence, RI, USA			0	14	3	17	3
Samuel	Lewis																				R	Seattle WA, USA			1			1	
Xiaoguang	Li						R	R	R	R	R											Shanghai, China	Y		5			5	
Suzanne	Lippert		Au	Au	Au	Au	Au	Ad														Palo Alto, CA, USA			0	5	1	6	

First Name	Last Name	Year, from 2005-2024																				Affiliation / Country	LMIC Yes	Other HIC	Years Review	Years Author	Years Advisor	Service Years	Targeted SR
		05	06	07	08	09	10	11	12	13	14	15	16	17	18	19	20	21	22	23	24								
Gideon	Loevinsohn																	R	R	R		Boston MA, USA			3			3	1
Richard	Lowsby													R	R	R	R					Melbourne, Australia; Cheshire, UK		Y	4			4	
Kevin	Lunney				R	R	R	R	R	R	Au	Au	Au	Au								Fresno, CA; Lejeune, NC, USA			6	4		10	
Heather	Machen											R										Houston, TX, USA			1			1	
Marlow	Macht				R	R																Denver, CO, USA			2			2	
Parker	Maddox																				R	Cincinnatti OH, USA			1			1	
Daniel	Magnus												R									Bristol, UK		Y	1			1	
Jacqueline	Mahal										R	R	R									Singapore; Bronx, NY, USA		Y	3			3	
Katie	Main																	R				Seattle, WA, USA			1			1	
Mallika	Manyapu																			R	R	Boston, MA, USA			2			2	
Regan	Marsh							R	R	Au	Au	Au	Au									Boston, MA, USA			2	4		6	
Michael	Mathelier																				R	Gainsville, FL, USA			1			1	
Sarah	McCuskee																R	R	R			New York, NY, USA			3			3	
Ruth	McQuillan																	T				Edinburgh, UK		Y	0			0	1
Ben	McVane													R								New York, NY, USA			1			1	
Rishi P	Mediratta													R	R							Stanford, CA, USA			2			2	
Edward	Melnick		Au	Ad																		Manhasset, NY, USA			0	1	1	2	
Rmaah	Memon																		R	R	R	Boston, MA; Philadelphia PA, USA			3			3	
Kevin J	Mercer																				R	Austin TX, USA			1			1	
Carl	Mickman														R							New York, NY, USA			1			1	
Daniel	Milikan				R	R	R	R	R	R	R	R										Ann Arbor, MI; Providence RI; Everett WA, USA			8			8	
Payal	Modi							R	R	R	R	R	R	Au	Au							Providence, RI; Boston MA, USA			6	2		8	
Kevin	Molyneux																				R	El Paso TX, USA			1			1	
Katelyn	Moretti															R	R					Providence RI, USA			2			2	
Stephen	Morris							R	R													Seattle, WA, USA			2			2	
Mwanja	Moses																			R		Kampala, Uganda	Y		1			1	
Terrence	Mulligan						R			R												Baltimore, MD, USA			2			2	
Dana	Naamani																			R	R	Durham, NC, USA			2			2	
Takashi	Nagata					R	R															Fukuoka, Japan		Y	2			2	
Theresa	Nguyen						R	R	R													Newark, DE, USA			3			3	
Tu Carol	Nguyen										R											Baltimore, MD, USA			1			1	
Benjamin D	Nicholson											R	R	R	R	R	Au	Au	Au	Ad	Ad	Boston, MA; Richmond VA, USA			5	3	2	10	
Jeffery A	Nielson		Au	Ad																		Akron, OH, USA			0	1	1	2	
Gerard	O'Reilly														R	R		R	R	Ad	Ad	Melbourne, Australia		Y	4		2	6	2
Maxwell	Osei-Ampofo								R	R	Au	Au	Au	Au	Au							Kumasi, Ghana	Y		2	5		7	1
Kevin C	Osterhoudt													R								Philadelphia, PA, USA			1			1	
Hannah Ofosua	Owusu																		R	R	R	Kumasi, Ghana	Y		3			3	
Mayur	Patel																			R	R	Gainsville, FL, USA			2			2	
Usha	Periyanayagam						R	R	R	R	R											Chicago, IL, USA			5			5	
Jennifer	Pigoga																R					Cape Town, South Africa	Y		1			1	
Amelia	Pousson				R								R	Au	Au	Au	Au	Ad	Ad			Newark, DE; Baltimore MD, USA			2	4	2	8	
Kimberly	Pringle					R	R	R	R													Providence, RI, USA			4			4	
Nana Serwaa Agyeman	Quao												R	R	Au	Au	Au	Au	Au	Au	Au	Kumasi, Ghana	Y		2	7		9	
Amritha	Raghunathan				R																	Palo Alto, CA, USA			1			1	
Kyle	Ragins													R	R							Los Angeles, CA, USA			2			2	
Najeeb	Rahman										R	R										Al Ain, UAE		Y	2			2	
Nicolas	Ramsay																		R			Chicago, IL, USA			1			1	

First Name	Last Name	Year, from 2005-2024																				Affiliation / Country	LMIC Yes	Other HIC	Years Review	Years Author	Years Advisor	Service Years	Targeted SR
		05	06	07	08	09	10	11	12	13	14	15	16	17	18	19	20	21	22	23	24								
Sarah	Rapaport																				R	Boston MA, USA			1			1	
Chris	Rees													R		R	R	Au	Au	Au	Au	Boston, MA; Atlanta GA, USA			3	4		7	
Pryanka	Relan													R	R			R	R	R		New York, NY; Atlanta, GA; New York NY, USA			5			5	4
Daniel	Roberts																	R	R	R		East Sussex, UK		Y	3			3	
Stephanie	Rosborough		Au	Au	Au	Au	Ad															Cambridge, MA, USA			0	4	1	5	
Charlotte	Roy														R	R	R	Au	Au			Chicago, IL; New York NY, USA			3	2		5	2
Michael	Runyon							R	R	R												Charlotte, NC, USA			3			3	
Megan	Rybarczyk											R	R	R	R	Au	Au	Au	Au	Ad	Ad	Boston, MA, USA			4	4	2	10	
Julia	Schiff																		R	R		Philadelphia, PA, USA			2			2	
Jessica	Schmidt																			R	R	Madison, WI, USA			2			2	
Erika Dee	Schroeder					R	Au	Au	Au	Au	R	R										Washington, DC; Everett WA, USA			3	4		7	
Megan	Schultz												R	R	R						Ad	Milwaukee, WI, USA			3		1	4	4
Anand	Selvam										R	R	R	R	Au	Au	Au	Au	Ad	Ad	Ad	Cincinati, OH; New Haven CT, USA			4	4	3	11	2
Hayley	Severson																			T		Milwaukee, WI, USA			0			0	1
Yusra	Shakil																				R	Lusaka, Zambia	Y		1			1	
Erin	Shufflebarger																			R	R	Birmingham, AL, USA			2			2	
David	Silvestri										R	R	R	R								Boston, MA; New Haven CT, USA			4			4	1
Branden	Skarpiak																R		R	R	Au	San Antonio, TX; Aurora CO, USA			3	1		4	
Cheyenne	Smith																				R	Philadelphia PA, USA			1			1	
Nagi	Souaiby								R	R												Beirut, Lebanon	Y		2			2	
Kimberly	Stanford													R	R	R						Chicago, IL, USA			3			3	
Daniel	Still																	R	R			Cape Town, South Africa	Y		2			2	
Jonathan	Strong															R	R	R	R	Au	Au	Boston, MA, USA			4	2		6	
Janet Jebichii	Sugut																			R	R	Nairobi, Kenya	Y		2			2	
Timothy	Tan						R	R														Boston, MA, USA			2			2	
Zachary D	Tebb		Au	Ad	Ad																	Denver, CO, USA			0	1	2	3	
Fadila	Tekka																			R	R	Dar es Salaam, Tanzania	Y		2			2	
Sabrina	Titze										R											Hennigsdorf, Germany		Y	1			1	
Carlos	Torres					R																Columbus, OH, USA			1			1	
Indi	Trehan									R	R	Au	Au	Au	Au	Au	Au	Ad	Ad			St. Louis, MO, USA			2	6	2	10	5
Lara	Vogel													R	R	R	R					Boston, MA; Salem MA, USA; Christchurch New Zealand		Y	4			4	
Benjamin	Wachira									R												Nairobi, Kenya	Y		1			1	
Alex	Wang														R	R	R	R	R	R		Hartford, CT, USA			6			6	1
Charles	Washington					R	R															Albuquerque, NM, USA			2			2	
Katherine	Wegman															R	R					Boston, MA, USA			2			2	
Tyson	Welzel										R											Cape Town, South Africa	Y		1			1	
W. Tyler	Winders											R	R	R					Ad	Ad	Ad	Cincinati, OH; Charleston SC; Hannibal MO, USA			3		3	6	4
Ann	Wolski																				R	Cincinnatti OH, USA			1			1	
Ambrose	Wong							R	R	R												New York, NY, USA			3			3	
Natalie	Yabalwashi																				R	Lusaka, Zambia	Y		1			1	
Wah Hon	Yau								R	R												Hong Kong	Y		2			2	
Ayalew	Zewdie													R	R							Addis Ababa, Ethiopia	Y		2			2	
																						Totals (n)	32	27	471	201	78	750	
