# Supplementary material for: Global Emergency Medicine: A Scoping Review of the Literature From 2024
Source: Acad Emerg Med. 2025 Dec 23;33(3):e70208. doi: 10.1111/acem.70208 (PMC12925323; doi:10.1111/acem.70208)
Supplement: Supplementary file 7 — Data S7: acem70208‐sup‐0007‐Supinfo6.pdf. [file ACEM-33-0-s004.pdf]

RE
DHR

Cameron L, McCauley M, van den Broek N, McCauley H. The occurrence of and factors associated with mental ill-health amongst humanitarian aid workers: A systematic review and meta-analysis. PLoS One. 2024; 19(5).

Humanitarian aid workers across a range of humanitarian settings face high rates of mental ill-health.

Summary: This systematic review and meta-analysis aims to describe the occurrence of and factors associated with mental ill-health among humanitarian aid workers globally. A comprehensive search was conducted in accordance with PRISMA guidelines among gray and published literature between 2005-2020 on various forms of mental ill-health among humanitarian workers. Studies were screened for inclusion by independent reviewers and underwent quality appraisal using the CCAT tool. A total of 9 studies met inclusion criteria with quality scores ranging from 63-93%, comprising 3619 total respondents. The reported pooled rates of mental ill-health were: 30.8% psychological distress (95% CI: 10.8–62.1%), 25.6% burnout (95% CI: 21.1–30.8%), 18.2% anxiety (95% CI: 8.9–33.7%), 24.1% depression (95% CI: 15.1–36.1%), and 9.6% post-traumatic stress disorder (95% CI: 2.8–28.2%). Two studies reported hazardous alcohol consumption as a form of mental ill-health, with rates ranging from 16-50%. Young age, female gender, pre-existing psychiatric condition, and exposure to traumatic events or stressors were found to be associated with mental ill-health. The authors conclude that high rates of mental ill-health among humanitarian aid workers warrant further attention and resources including expanded screening and treatment among aid organizations.

Comment: As the first systematic review of the mental health burden of humanitarian aid work, this study addresses an under-studied topic with increasing relevance as humanitarian needs rise over time. The study methods align with best practices and are comprehensive in scope, including grey literature and all published languages. Inclusion of all forms of humanitarian aid workers, from front-line healthcare providers to support staff in logistics, management, and administration, appropriately reflects the scope of humanitarian work while also introducing significant heterogeneity that limits direct comparisons and interpretability of pooled results. The study is further limited by heterogeneity introduced by inclusion of all forms of humanitarian crises (e.g., natural disasters, armed conflict, etc), as well as, both international and domestic aid workers. Additionally, studies utilized different mental health screening tools and predominantly relied on self-reported measures. Nonetheless, this study is an important attempt to better characterize the scope of the mental health burden among humanitarian aid workers and highlights the need for additional standardized research.

Samuel Lewis, Ashley Jacobson

RE
DHR

Davidson N, Hammarberg K, Fisher J. Ethical considerations in research with people from refugee and asylum seeker backgrounds: A systematic review of national and international ethics guidelines. J Bioeth Inq. 2024 Jun;21(2):261-84.

This systematic review of grey literature reveals scarce and heterogenous ethical guidelines for conducting research in refugee and asylum-seeking populations. The authors advocate for the development of specific ethical guidelines for these vulnerable populations.

Summary: The aim of this systematic review was to assess if refugee and asylum seekers are adequately protected via current research guidelines. The authors explained how these populations are especially prone to risk and harm, biases, and unethical approaches when being selected for, or participating in, health research. The authors followed PRISM recommendations and a pre-published protocol in their systematic search of grey literature. Their search included Google search terms, international health and governmental websites, and hand search of specific international and national websites listed in the International Compilation of Human Research Standards. Screening and subsequent data extraction were performed by one author. Fourteen articles were included in the final analysis from the initial 2187 articles screened. Data extracted and reported included: description of vulnerability, if ethical considerations were tied to these vulnerable populations, and furthermore if ethical considerations were specific to refugees and asylees. All included documents did agree on special protections for vulnerable groups; however, there is heterogeneity when it comes to which populations are considered vulnerable and what specific considerations are recommended or required. Four documents include refugees specifically and their need for special ethical considerations. After presenting how each individual guideline defines vulnerability and general considerations, the authors discussed if and how the included documents addressed autonomy, consent, justice, beneficence, and risk/benefit assessment.

Comment: Strengths of the review included the utilization of a prior published strategy for grey literature searches and following PRISM review guidelines. However, limitations included use of English-only searches and hand search of targeted websites. Bias might also have been introduced via screening, extraction, and interpretation all performed by one author. Of paramount importance, the authors targeted only high-income research settings, and did not include multinational, unstable, nor conflict settings. Research and guidelines pertaining to these settings would be essential in capturing displaced, vulnerable populations, and would be important for future research in the field of GEM and disaster response. These are particularly vulnerable populations, and progress made here would be broadly applicable. The article adds to current literature by consolidating current guidelines, revealing the lack of specific considerations and recommendations, and drawing attention to the need for context-specific, refugee-centered guidelines in research.

Mindi Guptill, Branden Skarpiak

OR
DHR

Khedr MA, Al-Ahmed NA, Mattar FK, Alshammari M, Ali EA. The feasibility of a psychological first aid intervention as a supportive tactic for feelings of psychological distress and mental health recovery outcomes among earthquake survivors in Northern Syria. *Int J Nurs Pract.* 2024;30:e13261.

The implementation of a psychological first aid intervention–which is grounded in psychological safety, cognitive reframing, mobilization of social support, and installation of hope–may have favorable outcomes in the event of public health emergencies, traumatic events, or even personal crises.

Summary: This was a quasi-experimental research study conducted among earthquake survivors in several refugee camps and shelters in Northern Syria. The study explored the feasibility and outcomes of implementing a psychological first aid intervention for psychological distress, resilience capacity, quality of life, and meaning of life among survivors. A pilot study with 10 participants was conducted prior to the commencement of the main research to confirm the study tools' feasibility and clarity. A convenience sample of 100 earthquake survivors were recruited, 95 of whom met the eligibility criteria. Forty-nine participants were allocated to the control group and 46 to the study group. The study group participated in a psychological first aid intervention which was delivered in 10 sessions, twice weekly, and concluded at a three-month follow-up. The control group was offered routine psychological support, such as watching videos related to stress management techniques. The study ultimately showed statistically significant improvements in resilience capacity, quality of life, and meaning of life measures. Moreover, compared with the control group, members of the study group had a significant reduction in psychological distress.

Comment: Psychological and psychiatric illnesses following traumatic events have been extensively reported. Interventions for these problems are therefore necessary because of the effect they can have on individuals and societies, long after the immediate post-disaster period. This study sought to investigate the feasibility of implementing Psychological First Aid (PFA) among earthquake survivors. One of the major strengths of this study was its experimental nature. The study also used varied instruments to measure the effect of the psychological first aid applied to the study group. An additional strength of the study included its measurements of mental health competencies before, immediately after, and three months after the interventions. This study does, however, have a few limitations, in particular the authors note a short follow-up period limits inferences beyond three months. Moreover, the study did not evaluate adolescents. More studies are needed to evaluate how psychological first aid can be applied to various experiences, including floods, outbreak emergencies, and conflict settings.

Hannah Ofosua Owusu, Joseph Leanza

RE
DHR

Tahernejad A, Sahebi A, Abadi ASS, et al. Application of artificial intelligence in triage in emergencies and disasters: A systematic review. BMC Public Health. 2024; 24:3203.

Use of artificial intelligence in the development of triage and assessment systems may allow for more efficient care and improved resuscitation of patients injured in disasters and emergencies.

Summary: In this systematic review, the authors explored the use of artificial intelligence in triage for injured patients. With injury rates from disasters and emergencies climbing worldwide, this technology is increasingly utilized to maintain efficiency and potentially improve clinical care. However, this has come with certain challenges which are explored in this study. The systematic review identified 19 publications on the use of artificial intelligence in triage. The authors found that use of this technology has improved patient care in emergencies and disasters by allowing for better assessment of injury severity, improved allocation of resources, efficient and timely triage, and improved decision making regarding patient transfers. For mass casualty incidents artificial intelligence systems may also enhance disaster response and allow for continuous, real-time monitoring and assessment of injured patients, ultimately resulting in more effective resuscitation. There were certain challenges identified in the implementation and use of artificial intelligence in triage, including confidentiality, training, equipment and resource restrictions, proper communication, and the understanding of the algorithms. Ultimately, the authors conclude that while there is certainly need for further research, the use of artificial intelligence in triage may improve patient care in disasters and emergencies.

Comment: Though this review provides valuable information on multiple aspects of the use of artificial intelligence in triage, heterogeneity in study designs also makes it difficult to draw definitive conclusions. Furthermore, most of the studies examined in this review were conducted in simulated environments, which may not fully capture the true nature of real disasters and emergencies. There may be value in the proposed use of this technology as there is a significant need for efficient triage and improved patient care during disaster events. This review provides an overview of the research that has been done on the use of artificial intelligence and machine learning in triage systems and details the areas of need for further research on the topic.

Aqeel Jawahir, Chris Rees

OR
DHR

Tesfay W, Abay M, Teklehaimanot BF, Gebremedhin A. Stabilizing time and its predictors among 1-59 months old children managed for severe acute malnutrition during the humanitarian crisis in Tigray regional state of Ethiopia, 2023: A prospective cohort study. BMC Pediatr. 2024; 24: 221.

Among children admitted to an Ethiopian hospital with severe acute malnutrition shortly after the Tigray War, early recovery was more likely in those who received oral rather than IV antibiotics, those who did not require blood transfusion or IV fluids, and those who tolerated oral rather than nasogastric tube feeds.

Summary: The existing paucity of information regarding severe acute malnutrition (SAM) treatment and outcomes in humanitarian emergencies is problematic because SAM rates climb during these emergencies and hospitals/treatment centers often find themselves without access to appropriate resources. In this prospective cohort study, researchers examined 184 children under 59 months old with SAM admitted to an Ethiopian hospital over a six-month period shortly after the ceasefire in the Tigray War. The vast majority of the children (177, 96.2%) recovered from SAM, whereas 2.2% eloped from the hospital and 1.6% died. The median time to stability was eight days, although children with edematous or kwashiorkor SAM had a statistically significant increased time of 10 days. Of 20 independent variables analyzed in relation to recovery time, five had statistically significant correlation with shorter time to recovery: patients who received oral rather than IV antibiotics; patients who did not require blood transfusions; patients who did not require IV fluids; patients who tolerated oral rather than nasogastric tube feeds; and patients who met indications for an appetite test (i.e. those greater than 6 months of age and those not admitted to the hospital for other, non-nutrition issues).

Comment: This study clearly illustrates some of the successes of SAM treatment in a recently war-torn region. The fact that treatment outcomes surpassed Sphere standards is hopeful, and the differences in recovery time among varied subsets of children provides a useful basis for future treatment priorities and future research alike. However, the article's scope is limited in that it does not describe overall rates of SAM, nor SAM outcomes among non-hospitalized children. Furthermore, all data was collected after the ceasefire, in the waning phase of Ethiopia's humanitarian crisis, making its significance to wartime SAM unclear. Further research is needed into rates of SAM and effective treatment strategies in conflict zones, to better allocate resources and target solutions.

Ann Wolski, Jennifer Jones

OR
ECRLS

Adal M, Tareke AA, Bogale EK, Anagaw TF, Tiruneh MG, Fenta ET, et al. Mortality of traumatic chest injury and its predictors across sub-Saharan Africa: Systematic review and meta-analysis. BMC Emerg Med. 2024;24:14.

This systematic review and meta-analysis aimed to assess mortality rates and identify factors contributing to death from traumatic chest injuries in sub-Saharan Africa. The pooled mortality rate was calculated at 9%, with several clinical factors identified as significant predictors.

Summary: The goal of this article was to determine the combined mortality rate for traumatic chest injuries and pinpoint key predictors in sub-Saharan Africa. To achieve this, the authors conducted a systematic review and meta-analysis in accordance with the PRISMA guidelines. They included 21 observational studies from major databases. The results showed a 9% pooled mortality rate (95% CI: 6.35–11.65). Significant predictors of mortality included age over 50; delays in hospital admission; pre-existing health conditions; additional injuries to the head, neck, spine, or heart; high injury severity scores; and the need for ICU care.

Comment: The study stands out due to its rigorous search strategy and adherence to existing methodological standards (e.g., PRISMA, Newcastle-Ottawa Scale). A key limitation, however, is that all included studies utilized observational designs. These findings highlight the urgent need for improved trauma care systems and effective treatment protocols in resource-limited settings. This research adds valuable insights to the global trauma literature by highlighting the specific challenges and mortality risk factors unique to sub-Saharan Africa, a region with limited emergency care infrastructure, limited resources, and a heavy trauma burden.

Yusra Shakil, Vinay N. Kampalath

RE
ECLRS

Ahmad AA, Mojiri ME, Daghriri AA, Hakami OA, Alruwaili RF, Khan RA, et al. The role of telemedicine in emergency department triage and patient care: A systematic review. Cureus. 2024; 16(12): E75505.

This systematic review looked at the breadth and depth of telemedicine in the ED via six RCTs. They conclude telemedicine has potential in triage and non-critical care, but call for more research in high acuity settings and improved regulations and policies.

Summary: The authors performed a rigorous and standardized systematic review of all global literature on the use of telemedicine in triage and patient care in emergency departments (EDs). Partly as a result of the pandemic, telemedicine use has grown substantially in ED settings. The authors aimed to look specifically at the accuracy of telemedicine for triage and diagnosis, patient satisfaction and outcomes, and its impact on throughput times and rates of readmission. They performed a comprehensive search of all English-language articles on the topic through November 2024. Ultimately, six studies met their quality metrics and were included in the descriptive analysis. None were suitable for a planned meta-analysis. The studies took place in low-, middle-, and high-income countries, and all addressed different clinical questions. The authors found that telemedicine was a reasonable alternative for managing low acuity patients (e.g., upper respiratory infections, rashes, minor allergic reactions and wounds), improved patient satisfaction, and decreased ED visits when used as a tool for follow-up after ED visits. Teletriage and telemedicine subspecialty consultation were generally felt to be of acceptable accuracy, and patients were generally satisfied with the inclusion of telemedicine in their care. Findings were equivalent on a change in throughput times and ED length of stay across the studies. One study, looking at the use of telemedicine in supervising intubations performed by ED residents found no significant difference in time to intubation, success rate, or complications. The authors acknowledge the heterogeneous nature of the studies, small sample sizes, and inability to do a blinded RCT with this modality. They ultimately conclude that telemedicine appears to have a role in low acuity diagnosis, reducing return visits, and enhancing patient adherence to care plans. The safety and efficacy of telemedicine for critical scenarios that require hands-on assessment or procedural intervention is still limited.

Comment: This article aimed to take a comprehensive and robust review of all existing literature on telemedicine in an ED context. While the literature captured in this review is limited, authors found that telemedicine may have positive roles in triage, patient care (both during and after the acute visit), and procedural oversight. There is a clear need for further study, particularly into regulatory frameworks that acknowledge the complexity of the situations in which EDs may use telemedicine.

Whitney Bryant, Nanaba Dawson-Amoah

OR
ECRLS

Arora D, Choudhary IS, Dutt A, Banerjee N, Chauhan AS, Rodha MS, et al. Efficacy of slow negative pleural suction in thoracic trauma patients undergoing tube thoracostomy: A randomised clinical trial. Injury. 2025; 56:1.

Continuous wall suction was superior to standard water seal for chest tube management in trauma patients.

Summary: This unblinded, randomized controlled trial conducted in India compared slow negative pleural suction to water seal drainage of adult trauma patients requiring chest tube placement. The experimental group had the chest tube connected to wall-mounted suction at -20cm water pressure, and the control group had the chest tube set to water seal using a two-compartment system. Sixty-four patients were randomized to a group and analyzed. Patients requiring thoracotomy, mechanical ventilation, or bilateral thoracostomy were excluded. The authors evaluated baseline demographics, with a majority of patients being male and suffering from blunt trauma. There was no statistically significant difference between the groups and their indication (e.g., hemothorax, pneumothorax, or hemopneumothorax). Chest tubes were discontinued when there was less than 200 mL/day of non-bloody output. The authors analyzed a series of outcomes, with the primary outcome being the duration of chest tube placement and length of hospital stay, both of which were significantly shorter in the experimental group. The average duration of tube placement was three versus five days in the experimental and control groups, respectively. Length of stay was five versus ten days, respectively. Adverse events were rare in both groups, with two deaths in the control group and one death in the experimental group. This study demonstrated superior outcomes when non-emergent chest tubes were placed to low-continuous wall suction versus water seal.

Comment: This study aimed to address the burden of hospitalization duration related to chest tube placement in trauma settings. The authors demonstrated improved outcomes of length of stay and duration of tube insertion when placing tubes immediately to low-continuous suction until tube removal. While the study results are compelling for settings in which low-continuous wall suction is an option, more details from the authors on the setup of their system could provide needed information for those wishing to change practice, as it was unclear whether a valve or water seal system was used in conjunction with suction. The authors do acknowledge the small sample size and the need for a larger study, but cite similar research with similar results, demonstrating a likely true benefit to the use of slow negative pleural suction for chest tube management in the setting of trauma. Larger trials would likely improve the sensitivity for assessing rare adverse events.

Tal Berkowitz, Ashley Jacobson

OR
ECLRS

Bonnet G, Bimba J, Chavula C, Chifamba HN, Divala TH, Lescano AG, et al. Cost-effectiveness of COVID rapid diagnostic tests for patients with severe/critical illness in low- and middle-income countries: A modeling study. PLoS Med. 2024;21(7): e1004429.

The use of rapid diagnostic tests is cost-effective in severe COVID cases when there is high prevalence of COVID and rapid testing is available, regardless of country income level. In low-income, resource-limited settings, testing is most cost-effective when there is high prevalence of COVID and less cost-effective when there is low prevalence of disease.

Summary: This study assessed the cost-effectiveness of COVID rapid diagnostic tests (RDTs) in 129 low- and middle-income countries (LMICs) using disability-adjusted life years (DALYs) averted over a lifetime. There were five testing options for patients when there was a clinical suspicion of having COVID: no testing, using RDTs alone, using PCR alone, using RDTs and confirming negative tests with PCR, and using RDTs and confirming positive tests with PCR. A decision tree was utilized to compare the downstream costs associated with different testing methods, such as antibiotic use, corticosteroid use, the need for mechanical ventilation, and other scenarios. The study demonstrates that the cost-effectiveness of RDTs varies according to local COVID-19 prevalence, influenza prevalence, and the severity of resource limitation among LMICs. In poorer LMICs, patients with a high index of suspicion for COVID should be tested with RDTs. This is especially true in areas with high prevalence of influenza, because corticosteroids used to treat severe COVID may worsen influenza outcomes, making RDTs more cost-effective. In wealthy LMICss and upper-middle-income countries (UMICs), testing of suspected severe COVID is almost always recommended. At low COVID prevalence, treating patients as if they did not have COVID is the most cost-effective option in low-income countries, as influenza may mimic COVID. As prevalence increases, testing with RDTs becomes more cost-effective in most low-income countries. At very high prevalence, presumptive treatment is the most cost-effective option. In UMICs, testing is always cost-effective, even at low COVID prevalence.

Comment: This article aims to assess the cost effectiveness and feasibility of testing for COVID in LMICs. Multiple approaches were used, including a decision tree, healthcare provider perspectives, and cost-benefit analysis. The authors note that there is a complex picture between practical application and proposed treatment pathways due to variability in resources and disease prevalence. While there is no perfect decision tree to decide the testing approach for COVID in all settings, this study provides groundwork to develop general guidelines across different settings.

Kevin Molyneux, Joseph Ciano

OR
ECLRS

Colunga-Pedraza JE, Lopez-Reyna IG, Vaquera-Aparicio DN, Pena-Lozano SP, Arrieta J, Hernandez-Torres LE, et al. Overcoming challenges to reduce time to antibiotic therapy in febrile neutropenic children: Insights from a Mexican center. Hematol Transfus Cell Ther. 2024; 46: S193-201.

A "Golden Hour" intervention significantly shortened time to antibiotic treatment and improved clinical outcomes in pediatric hematology-oncology patients with febrile neutropenia. It highlights an effective intervention that can be implemented in other low- and middle-income countries.

Summary: This comparative observational study evaluated the impact of a multidisciplinary "Golden Hour" intervention conducted as part of the Mexico in Alliance with St. Jude (MAS) collaboration on antibiotic timing and clinical outcomes in pediatric hematology-oncology patients with febrile neutropenia. Retrospective data from pediatric hematology-oncology patients presenting with febrile neutropenia pre-intervention was compared to prospectively-collected data from pediatric hematology-oncology patients with febrile neutropenia post-intervention. Before the intervention, only 5.7% of patients received their first dose of antibiotics within one hour, which increased to 84.6% of patients in the post-intervention period. The median time to antibiotics improved from 269 minutes pre-intervention to 50.5 minutes post-intervention. In the post-intervention period there were significant reductions in patients who developed sepsis and who required admission to the PICU, as well as a reduction in length of hospital stay. This study demonstrated the effectiveness of the "Golden Hour" intervention in reducing the time to antibiotics and improving clinical outcomes.

Comment: While this is a single-center study, it highlights the effectiveness of a multimodal intervention that can improve clinical outcomes in a high-risk population. This intervention utilized a multidisciplinary team to improve the time to antibiotics for pediatric hematology-oncology patients and can potentially be adapted for use in other emergency departments in low- and middle- income countries once further research is conducted. One limitation of this study is its focus specifically on pediatric patients with hematologic malignancies, which limits the generalizability of this intervention to other populations. Further research could investigate the effectiveness of such an intervention in adults. Another limitation of this study is that it was conducted at a single center, and future research is needed to study the intervention in different settings.

Rmaah Memon, Morgan Broccoli

OR
ECLRS

Conradi N, Opoka R, Mian Q, Conroy A, Hermann L, Charles O, et al. Solar-powered O2 delivery for the treatment of children with hypoxaemia in Uganda: A stepped-wedge, cluster randomised controlled trial. Lancet. 2024; 10428: 756-65.

A stepped-wedge randomized controlled trial of 2,409 participants conducted in rural Ugandan hospitals demonstrated a statistical reduction in mortality of children 48 hours from detection of hypoxemia when provided access to solar-powered oxygen concentrator systems.

Summary: Lack of equitable access to supplemental oxygen has been a long-standing issue in low-middle income countries (LMICs), resulting in hypoxemic illnesses being a major source of mortality in pediatric patients. This study aimed to add to this literature by assessing the effects of introducing solar-powered O2 concentrators to hospitals in rural Uganda on the mortality of pediatric patients with hypoxemia. Twenty hospitals throughout Uganda enrolled 2,405 children with hypoxemia in a stepped-wedge cluster randomized control trial. Hospitals were selected if they had no or unreliable access to supplemental oxygen or facilities to support solar panels. Children were selected, with guardian informed consent, if they were younger than five years old and if they had symptomatic hypoxemia (cough or trouble breathing) resulting in hospital admission. Patients were then screened for hypoxemia with pulse oximetry and cough or shortness of breath. Children were then provided oxygen from a source, if available, based on randomized and stepwise installation of solar-powered oxygen concentrators throughout Ugandan hospitals, with a goal of oxygen saturation greater than 92%. The primary outcome for this study was 48-hour mortality after hypoxemia was detected. Results were analyzed utilizing a linear mixed effects logistic regression model and demonstrated a statistically significant relative reduction of 48.7% in 48-hour mortality. The primary and secondary outcomes of this study led the authors to conclude that solar-powered oxygen concentrators can reduce child hypoxemia deaths cost-effectively.

Comment: Access to supplemental oxygen is critical to the practice of emergency medicine globally and can be lifesaving. The current literature on solar-powered oxygen concentrators demonstrates their cost-efficacy, feasibility, and non-inferiority, but this study takes the next step by showing the mortality benefit in hypoxemic pediatric patients. This study has a high level of impact as these findings apply to the care of a vulnerable patient population, children, and demonstrate the efficacy of solar-powered oxygen concentrators in rural hospitals, which can be extrapolated beyond Uganda. The study is well planned as a stepped-wedge study with cluster randomization to circumvent the ethics of a non-treatment group. Limitations included a delay in equipment installation and a lack of blinding, but the authors addressed these by randomizing and concealing the order of equipment installation to the hospitals and performing a sensitivity analysis to account for delays. They also reduced bias by ensuring each hospital had all other standards of care available for hypoxemic illnesses and equally selecting hospitals across the geography of Uganda.

Parker Maddox, Vinay Kampalath

OR
ECLRS

Daihimfer F, Babamohamadi H and Ghorbani R. A comparison of the effects of acupressure and music on venipuncture pain intensity in children: A randomized controlled clinical trial. Pain Res Manag, 2024:2504732.

This randomized controlled trial involving 180 children aged three to six years in an Iranian emergency department found that both music and acupressure significantly reduced pain from venipuncture compared to no intervention, with music showing the greatest effect.

Summary: This randomized controlled clinical trial aimed to compare the effects of acupressure and music therapy on venipuncture pain intensity in children aged three to six years at Children's Medical Center Emergency Department at Tehran University of Medical Sciences. A total of 180 children were randomly assigned to one of three groups: music therapy, acupressure, or a control group receiving routine care. Interventions were administered for five minutes, starting three minutes before and continuing through the venipuncture procedure, with pain intensity assessed using the Oucher scale. The results showed that both music and acupressure significantly reduced pain compared to the control group, with mean pain scores of 3.32, 4.82, and 8.32, respectively. Pain levels in the music group were significantly lower than in both the acupressure and control groups ($p < 0.001$), and the acupressure group experienced significantly less pain than the control group ($p < 0.001$). The study concludes that while both methods are effective, music therapy is superior and should be considered a nonpharmacological approach for managing venipuncture pain in young children.

Comment: This article demonstrates several strengths in its design, including the use of a randomized controlled trial, and a reasonably large sample size of 180 children, enhancing the statistical power and reliability of the findings. The standardized administration of interventions, use of a validated pain scale, and clear inclusion/exclusion criteria contribute to the study's methodological rigor. However, the study has limitations, such as the inability to blind participants and staff, which may introduce observer or performance bias, and the use of convenience purposive sampling, which may affect generalizability. Additionally, the study focused only on a narrow age group (three to six years), limiting its applicability to older or younger children. Despite these limitations, the findings are important to the field of global emergency medicine, as they offer low-cost, nonpharmacological interventions that can be feasibly implemented in resource-limited emergency settings to improve pediatric patient care and reduce procedural pain. The article builds upon prior research demonstrating the benefits of distraction techniques and complementary therapies in pain management, while uniquely comparing acupressure and music directly in a pediatric emergency context, thus filling a gap in the literature and guiding future clinical practice and research.

Natalie Yabalwashi, Vinay N. Kampalath

RE
ECLRS

Endeshaw D, Delie AM, Adal O, Tareke AA, Bogale EK, Anagar TF, et al. Mortality and its predictors in abdominal injury across Sub-Saharan Africa: Systematic review and meta-analysis. BMC Emergency Medicine. 2024;1:57.

A meta-analysis helped determine predictive factors of mortality for abdominal trauma in sub-Saharan Africa, which could help tailor strategies to reduce mortality in this patient population.

Summary: Abdominal trauma is a major cause of morbidity and mortality globally, and can be especially consequential in certain sub-Saharan African contexts with limited healthcare infrastructure. The authors here conducted a systematic review and meta-analysis to explore predictors of mortality for abdominal trauma in this region, selecting 33 articles for analysis. The studies selected were published between 2000 and 2023 and reflected a total of 6,124 patients presenting with abdominal trauma. The authors conclude that patients who presented with shock, were admitted to the ICU, suffered blunt abdominal trauma, and those who experienced postoperative complications had a higher risk of mortality. The authors suggest that addressing these predictors in abdominal trauma can help reduce mortality.

Comment: This study provides an overview of predictive factors for mortality for patients with abdominal trauma in sub-Saharan Africa. The authors provide detailed methodology for the selection and analysis of the chosen articles, including a bias assessment. One weakness of the article is that the authors do not provide further direction on how their conclusions can be used to specifically reduce mortality from abdominal trauma. Another weakness is the authors only included English-language studies, limiting the variety of sources for analysis. This study has a significant impact in helping clinicians determine which patients may be at a higher risk for mortality to direct resources available to caring for these patients. As such, the study is important in emergency care in low-resource settings, and provides data from a sub-Saharan context.

Mayur Patel, Nanaba Dawson-Amoah

OR
ECLRS

Gyedu A, Amponsah-Manu F, Awuku K, Ameyaw E, Korankye KK, Donkor P, Mock C. Differences in trauma care between district and regional hospitals and impact of a trauma intake form with decision support prompts in Ghana: A stepped-wedge cluster randomized trial. World J Surg. 2024; 3: 527-39.

Utilizing a decision-support trauma intake form with clinical prompts improved initial trauma care both in district and regional hospitals in Ghana.

Summary: This quality improvement study assessed trauma care processes and key performance indicators (KPIs) at district and regional/referral hospitals in Ghana, evaluating how the use of a standardized trauma intake form (TIF) impacted care at each level. The TIF, a decision-support tool adapted from global best practices and tailored to the Ghanaian context, served as a checklist and provided real-time clinical prompts to support initial trauma care. Eight hospitals were sequentially introduced, in a stepped-wedge fashion, to the intervention over a 17.5-month period. Data collection included real-time direct observations using the TIF-based tool and medical record reviews. Among the 4,077 patients included, roughly half were in the pre-intervention group and half post-intervention, with two-thirds managed at district hospitals and one-third at regional hospitals. At baseline, district hospitals underperformed compared to regional hospitals in most trauma care indicators, such as vital signs monitoring and clinical documentation, though they more consistently considered pain management. Following TIF implementation, district hospitals showed notable improvement in 14 of 20 KPIs, particularly in airway assessment, chest examination, evaluation for intra-abdominal bleeding, and tetanus consideration for open wounds. Regional hospitals, meanwhile, improved in eight KPIs and achieved over 90% adherence to all measured indicators, continuing to outperform district hospitals despite the overall improvements.

Comment: Trauma care in resource-limited settings poses numerous challenges, and this study addresses some of these. It employed a pragmatic quality improvement approach, using a stepped-wedge cluster randomized design to implement and evaluate a locally adapted, decision-support intervention. The use of direct observations enhances data reliability, and the findings underscore the potential for simple, low-cost tools like the TIF to drive significant improvements in clinical practice. Nevertheless, variable implementation and the possibility of observer bias are some of the challenges encountered. Importantly, the study highlights persistent disparities in care quality between district and regional hospitals, with relative district hospital underperformance, emphasizing the need for tailored strategies to support lower-level facilities and ensure more equitable trauma care delivery.

Kimonia Bih Awanchiri, Chris Rees

OR
ECLRS

Jamarillo GD, Zuluaga NE, Avellaneda VAV, Ramirez SM, Neira FJP, Contador APL, Gallo JPV. Implementation of an early attention strategy to reduce emergency room overcrowding in an academic institution in Colombia: A pilot study. International Journal of Emergency Medicine. 2024; 17:152.

In this retrospective observational study, a physician-in-triage protocol for moderate complexity patients (triaged 3 on 1-5 scale) reduced patient length of stay in a Colombian emergency department.

Summary: The authors in this article developed and implemented a triage protocol with the goal of decreasing patient length of stay in the emergency department. This protocol places a physician in the triage area to rapidly evaluate and order studies and medications for each patient who is triaged level 3 on a 1-5 scale. Under this protocol, the physician in triage spends no more than 10 minutes evaluating each patient, then the patient waits for a definitive consultation by another physician. This study was retrospective and observational in design and compared adult patients who went through the typical care process for one month with adult patients who underwent the provider-in-triage care process for one month. A total of 969 patients were included in the study. The authors found that involving a physician at triage resulted in decreased times between definitive consultation and disposition (discharge or admission) ($p =< 0.001$). Patient length of stay in the emergency department also decreased under this new protocol ($p = 0.037$).

Comment: Research shows that boarding and extended stays in the emergency department are associated with an increase in medical complications. The authors note there is limited research on emergency department patient flow in Colombia. The findings of this study are promising in that the model reduces the time to several key metrics in emergency department flow: consult time to disposition and emergency department length of stay. While the time to disposition and length of stay in the emergency department was reduced, the study also reported that boarding time, defined as the time between disposition decision and physical transfer of the patient to the ward, remained the same between the standard and the study protocol. This raises a question as to whether this protocol could directly improve department overcrowding. Further limitations include: sample size and differences between group characteristics, retrospective nature of study, and the use of protocol limited to eight hours a day on weekdays. Additional research is needed to investigate the sustained use of this protocol, protocol expansion to other triage levels, and the interactions between the intervention and emergency department boarding.

Jonathan Chan, Branden Skarpiak

Karamian A, Seifi A, Karamian A, Lucke-Wold B. Incidence of intracranial bleeding in mild traumatic brain injury patients taking oral anticoagulants: A systematic review and meta-analysis. J Neurol. 2024; 271:3849–68.

This meta-analysis demonstrates a high rate of intracranial hemorrhage, 9.4%, from mild traumatic brain injury (TBI) for patients on oral anticoagulation.

Summary: Traumatic brain injury (TBI) is a major cause of death and disability worldwide, especially as populations age and the use of oral anticoagulants increases. Presentations for mild TBI, for instance due to falls, are increasingly common in emergency departments. This systematic review and meta-analysis aimed to assess the incidence of intracranial hemorrhage (ICH) resulting from mild TBI in patients on oral anticoagulation. The study was conducted using the PRISMA checklist and included English-language studies evaluating mild-TBI in patients taking Vitamin K antagonists (VKAs) or direct oral anticoagulants (DOACs) and evaluated immediate and delayed events, when available. Independent reviewers screened the initial 19,651 studies to identify 28 studies included in the final analysis. These studies comprised 11,172 patients from ten high to upper-middle income countries, with 5,671 patients on DOACs, and 5,501 patients on VKAs. The overall incidence of ICH was 9.4%. The rate of immediate ICH was found to be 8.5%; with a lower rate for patients taking DOACs (6.4%) than VKAs (10.5%). The incidence of delayed ICH was 1.7%. There was significant heterogeneity between studies including three studies which reported depressed skull fracture as an intracranial traumatic lesion. The rates were much higher for immediate versus delayed injury, leading the authors to emphasize the importance of an initial head CT.

Comment: This meta-analysis highlights the relatively high incidence of ICH for anticoagulated patients with mild TBI, which is a growing concern in aging populations. The study strengths include its large sample size and subgroup analysis comparing DOACs and VKAs. The overall incidence in this study was similar to results of a previous meta-analysis by Mihas et al. (2018), supporting the validity of the study's statistical model. The analysis was limited by the substantial heterogeneity between the studies, which the authors addressed using random-effects modeling. Another limitation of the study was its geographic representation, including primarily high income countries in Europe and North America, thus limiting the study’s generalizability to lower-resource settings. The largest limitation was the clinical relevance of its outcomes; while the study reflects the overall incidence of ICH, the analysis did not include clinical endpoints such as need for surgery, resulting disability, or even mortality. While it is important to know the true incidence of radiographic findings, it is also important to understand the clinical implications, especially for regions of the world where access to immediate head CT may not be readily available. For global emergency medicine, the relevance of clinically-significant ICH is more impactful as it sheds light on which patients can be safely observed without the need for imaging.

Jessica Schmidt, Nanaba Dawson-Amoah

OR
ECLRS

Kefyalew M, Deyassa N, Gidey U, Temesgen M, Mehari M. Improving the time to pain relief in the emergency department through triage nurse-initiated analgesia: A quasi-experimental study from Ethiopia. Afr J Emerg Med. 2024 Sep; 14:161-6.

Nurse-led analgesia in an Ethiopian emergency department reduced the time to pain medication administration and improved patient satisfaction.

Summary: Pain management is crucial in the emergency department (ED), yet there is a lack of data on pain prevalence and treatment in Africa. With undertreatment of pain being a common finding in the ED, this study proposes a nurse-led analgesia protocol in the ED to shorten the time to pain treatment and improve patient experience. The study employed a quasi-experimental design in two EDs in Ethiopia, comparing a nurse-led analgesia protocol (Tikur Anbessa Specialised Hospital, TASH) with the standard of care (Kidus Paulos Specialised Hospital, KPH). The study results demonstrated a shorter average time to analgesia in the intervention group (89 min) compared to the control group (420 min). The differences in time to analgesia and patient satisfaction were statistically significant ($p < 0.01$). However, there was no statistically significant association between length of stay and nurse-led analgesia ($p = 0.29$).

Comment: One of this study's strengths is that it was conducted in two large hospitals in Ethiopia that serve a large population. Additionally, researchers used a previously validated, standardized tool to assess the knowledge and practices of healthcare workers. One limitation is the intended sample size of 218 was not achieved, which limits the precision of the results. Additionally, only a small proportion of the ED staff consented and were trained; the study does not report how many nurses were eventually recruited after the post-test. This study is important for the African context, where the prevalence of pain treatment is understudied, and workforce challenges persist in most EDs. This nurse-led analgesia protocol in the ED has the potential to significantly improve time to treating pain and patient satisfaction.

Janet Sugut, Vinay N. Kampalath

OR
ECLRS

Legesse AT, Kejela S, Tesfaye AS, Gebremariam MS, Hailu MA, Workneh F, et al. Validation of the Raja Isteri Pengiran Anak Saleha Appendicitis (RIPASA) scoring system for the diagnosis of acute appendicitis among Ethiopian patients: A multi-institutional observational study. BMC Surg. 2024;24:218.

This study validates the RIPASA scoring system as a reasonable alternative for diagnosing acute appendicitis in resource-limited settings where imaging, particularly ultrasound, is not always accessible.

Summary: This multi-institutional observational study aimed to evaluate the Raja Isteri Pengiran Anak Saleha Appendicitis (RIPASA) scoring system in comparison to ultrasound for diagnosis of acute appendicitis, the most common surgical emergency in Ethiopia. A total of 315 patients with signs and symptoms of acute appendicitis were assessed using both ultrasound examination and RIPASA scoring, with results compared to intraoperative findings. The RIPASA scoring demonstrated a sensitivity of 96.2% and specificity of 30.8%, non-inferior to ultrasound sensitivity of 95.3% and specificity of 27.8%. Of those with appendicitis suggested by RIPASA and ultrasound, the intraoperative negative appendectomy rate was 3.8% and 4.7% respectively. The study concludes that given the high concordance rate, good sensitivity, and positive predictive value of both the score and ultrasound, the RIPASA is a valid tool for clinical practice in low-resource environments where a radiographic diagnosis is not always available.

Comment: This study provides a valuable tool for clinicians practicing in areas where acute appendicitis is a common surgical emergency but radiologic resources for diagnosis may be inaccessible. Its strengths lie in its prospective, multi-center design. A limitation recognized by the authors was the use of intraoperative gross examination rather than histopathology for confirming appendicitis, potentially affecting diagnostic accuracy. This research is important for global emergency medicine literature as it highlights a practical scoring system for diagnosing appendicitis that performs comparably to ultrasound for use in settings where radiologic diagnosis may not be accessible. This study supports and extends prior research validating RIPASA in other populations, reinforcing its utility as a robust alternative for diagnosis of acute appendicitis.

Erin F. Shufflebarger, Amanda Collier

RE
ECLRS

Quake SYL, Khoda F, Rad AA, Ponniah HS, Vardanyan R, Frisoni P et al. The current status and challenges of prehospital trauma care in low- and middle-income countries: A systematic review. Prehosp Emerg Care. 2024; 28:76-86.

This systematic review summarizes the current variable development of prehospital trauma care systems in LMICs, highlights common barriers to development and implementation, and presents successful case studies which can inform future efforts in low-resource settings.

Summary: Traumatic injuries cause significant morbidity and mortality globally, and have a disproportionate impact in low- and middle-income country (LMIC) settings, where the majority of deaths due to trauma occur. Prehospital emergency medical services (EMS) are an important component of systems of care for patients with traumatic injuries, however there has been limited research regarding pre-hospital care systems strengthening. This systematic review focused on published reports on the status of pre-hospital trauma care provision in LMIC settings, with a focus on the challenges encountered in pre-hospital trauma care system development and improvement in these settings. The authors included original research in the published literature on prehospital trauma care in LMICs from 2010 to 2022. After searching multiple databases, 1,164 original records were screened, and 29 papers met criteria for inclusion. Overall, the state of development of EMS systems for trauma care in LMICs is highly variable. The most common challenges for prehospital trauma care system development were funding, healthcare workforce limitations, and access to equipment and supplies. A common theme described in the included studies was increased utilization of lay providers for prehospital transportation and initial first aid. In terms of healthcare-seeking behavior, barriers to EMS utilization included inconsistent availability of national emergency telephone numbers, variation in geographic access to services, and low public knowledge of available services. The authors recommend utilizing successful examples of development to inform work in other contexts, as well as the need for ongoing research in pre-hospital trauma care system development.

Comment: Strengths of this review include a search of multiple databases and reproducible methodology for inclusion/exclusion criteria. The authors also thoughtfully synthesize and categorize key patterns and barriers across the articles. Limitations include: marked variability in study design and measures, lack of any formal quality assessment, and heterogeneity in socio-political contexts. This data was not amenable to meta-analysis. The review provides insight into the current status of development for pre-hospital trauma care systems via English-language studies; the positive examples and recurrent barriers found in these studies may inform future research and policy priorities in this field.

Emily Bartlett, Branden Skarpiak

OR
ECLRS

Rahmani C, Belhadj A, Aissaoui Y. Can plethysmographic capillary refill time predict lactate during sepsis? An observational study from Morocco. Afr J Emerg Med. 2024;14(3):167-71.

In a prospective observational cohort, capillary refill time was assessed visually and with a pulse oximeter and found to be a cost-effective, non-invasive way to measure tissue perfusion. Capillary refill time was found to correlate with serum lactate levels in patients with sepsis or septic shock.

Summary: This study aimed to test the accuracy of capillary refill time measured visually (V-CRT) compared to plethysmographic capillary refill time (P-CRT) in predicting lactate levels among patients who are septic. This prospective observational study enrolled patients consecutively with sepsis or septic shock over nine months from a tertiary referral hospital in Morocco. Fingernail beds were blanched with direct pressure and the time to normal nailbed color return or for the plethysmographic signal to return to baseline was measured. The precision of V-CRT and P-CRT on admission in predicting arterial lactate was assessed using ROC curve analysis. Forty-three patients with a mean age of 65 years were enrolled, 23 with sepsis and 20 with septic shock. Both V-CRT and P-CRT correlated with arterial lactate (correlation coefficients 0.529 and 0.517, respectively). V-CRT exhibited satisfactory accuracy in predicting arterial lactate levels >2 mmol/l with an area under the curve (AUC) of 0.8 (95% CI=0.65-0.93). P-CRT had lower prediction ability with an AUC of 0.73 (95% CI=0.57-0.89).

Comment: Given the limited availability of lactate in many resource-limited settings, this study introduces two cost-effective options, P-CRT and V-CRT, that were found to correlate with lactate in septic patients in this prospective cohort. Previous studies have demonstrated that using the plethysmographic curve to measure CRT could assist in serum lactate prediction. This study, however, suggests that P-CRT, which is slightly more resource intensive than V-CRT, did not improve the accuracy of V-CRT for predicting lactate level. V-CRT alone may serve as a viable surrogate for lactate in septic patients. The study is limited as it took place at a single site with a low sample size, and also did not collect data on patient skin color, which could limit generalizability. However, the study met its goal to collect preliminary data and makes a convincing argument that the V-CRT threshold could be used in lieu of lactate to redefine septic shock criteria in low-resource settings. Future studies with a larger sample size at more diverse sites are needed.

Elizabeth M Keating, Morgan Broccoli

OR
ECLRS

Rahnemayan S, Ala A, Taghizadeh N, Sadeghi-Hokabadi E, Entezari I, Shamsvahdati S. Shortened NIHSS for rapid stroke assessment in emergency care settings. Neurologist. 2025;30:150-4.

The NIHSS-8, a shorter version of the original stroke scale, is a potentially reliable and efficient alternative to the NIHSS-11 for the diagnosis of stroke in emergency care settings.

Summary: This cohort study, conducted at Imam Reza Hospital in Tabriz, Iran, assessed the reliability of the NIHSS-8, an abbreviated version of the NIHSS-11, for the diagnosis of acute stroke. Patients with a confirmed diagnosis of stroke were included in the study and were independently evaluated by an emergency physician and a neurologist who administered the NIHSS-8 and NIHSS-11 respectively and were blinded to each other's scores. The sensitivity and specificity of each scale for the diagnosis of stroke, as measured with the modified Rankin scale (mRS), as well as likelihood ratios were calculated and compared. The study demonstrates that the NIHSS-8 (sensitivity 97.5%, specificity 96.9%) is almost equally as sensitive and specific as NIHSS-11 (sensitivity 100%, specificity 96.9%) for the diagnosis of a stroke in this emergency care setting. This study adds to the body of evidence supporting the use of the NIHSS-8 as a reliable and efficient alternative to the NIHSS-11 in low-resource emergency settings.

Comment: This study is useful for the evaluation and management of acute strokes in emergency care settings, particularly in low-resource settings with the potential for limited staffing or availability of consultant neurologists. The study has multiple strengths. For each patient included in the study, the NIHSS-8 was conducted by an emergency physician while the NIHSS-11 was conducted by a neurologist, and the two physicians were blinded to each other's scores to minimize bias. As the NIHSS-8 was administered by an emergency physician, the results of the study are more generalizable to settings where a neurologist may not be available. The study also had some limitations; since it only included patients with a confirmed diagnosis of acute stroke, the results cannot be generalized to all patients presenting with symptoms concerning for a stroke. Also, knowledge of a patient already having the diagnosis of stroke may have led to more thoughtful and exacting assessments than may otherwise be done in some emergency care settings, and also limits the ability for the score to assess the abbreviated scale's specificity for stroke.

Dana Naamani, Morgan Broccoli

OR
ECLRS

Ramamoorthy T, Ayyan SM, Deb AK. Diagnostic value of point-of-care ultrasound-guided assessment of relative afferent pupillary defect in adult ocular trauma patients presenting to the emergency department: A prospective cohort study. J Ultrasound Med. 2024; 43: 1343-51.

Emergency physician performed POCUS showed high sensitivity and specificity in detecting relative afferent pupillary defect (RAPD) in a prospective cohort of adult ocular trauma patients in an Indian emergency department.

Summary: Ocular trauma is a common injury in trauma patients, especially those with head injuries. A portion of these patients have relative afferent pupillary defect (RAPD) as a symptom of severe eye injury that needs immediate care. Point of care ultrasound (POCUS) can be used for eye examination, particularly when periorbital ecchymosis is present and makes clinical examination difficult. This single-center, prospective cohort study in an Indian ED investigated eye examination for RAPD, comparing POCUS (performed by a single trained ED physician) and clinical examination. Out of 376 patients with eye trauma, 55 (14.6%) had RAPD diagnosis (assessed by an ophthalmologist as gold standard). Compared to clinical examination, POCUS showed a higher sensitivity (92.7 vs. 81.8), specificity (99.4 vs. 99.1), positive predictive value (96.2 vs. 93.8) and negative predictive value (98.4 vs. 96.5). This led to a positive likelihood ratio of 148.8. Data suggests that POCUS performed by ED physicians is a useful tool for eye examination after trauma for detecting RAPD as a sign of severe eye injury.

Comment: POCUS has become an indispensable tool in EM diagnostics, with an increasing number of indications for its use. Examination of the eyes after trauma, especially in patients with periorbital ecchymosis can be challenging. This prospective cohort trial shows higher sensitivity and specificity compared to expert clinical examination for identifying RAPD. Limitations of this trial included being a single center trial and utilizing only one well-trained POCUS examiner compared to non-standardized clinical examination during initial trauma assessment. The study protocol was planned for 94 RAPD cases but only 55 were included - the authors explain they used a study conducted in an ophthalmology department to calculate sample size, which may have overestimated the potential incidence in the emergency department. The authors also note that a high prevalence of alcohol consumption in their region could confound RAPD detection and affect generalizability. Finally, clinical outcomes of patients found to have RAPD were not studied. In summary, POCUS, which is frequently available even in low-resource settings, can be a useful tool for detecting RAPD particularly in cases with significant periorbital ecchymosis.

Christian Engelen, Amanda Collier

RE
ECLRS

Ranjbar Hameghavandi MH, Khodadoust E, Tabatabaei MSHZ, Farahbakhsh F, Ghodsi Z, Rostamkhani S, et al. Challenges in traumatic spinal cord injury care in developing countries: A scoping review. Front Public Health. 2024;12:1377513.

This scoping review identified 82 articles summarizing challenges in traumatic spinal cord injury (TSCI) prevention and management in low- and middle-income countries.

Summary: The burden of morbidity and mortality due to traumatic spinal cord injury (TSCI) is disproportionately higher in low- and middle-income countries compared to high income countries. This study focused on identifying challenges in preventing and managing TSCI in developing countries to establish a conceptual framework and ultimately improve care. After a systematic search of online databases, the authors included 82 articles from 26 countries in this review and classified them into four domains of TSCI care: prevention, pre-hospital care, in-hospital care, and post-hospital care. Most articles focused on in-hospital and post-hospital care, with fewer articles focused on primary prevention. Challenges in injury prevention included deficiencies in infrastructure, poor supportive legislation, and insufficient public education. Common challenges identified in pre-hospital and in-hospital care included a lack of trained human resources, lack of standardized guidelines for the management of acute TCSI, and delays in transportation and care delivery. Studies on post-hospital care focused on challenges faced by TCSI patients related to insufficient supplies and equipment, lack of access to rehabilitation services, insufficient education, and lack of employment opportunities. The study highlights a critical insufficiency of data on prevention and pre-hospital care for TSCI patients in developing countries and recommends prioritizing research in these areas.

Comment: This article explores the diverse challenges affecting the prevention and delivery of care for patients with TSCI in developing countries. The authors conducted an extensive review of high-impact studies on this topic, which strengthens the external validity of their findings across various settings and populations. However, by not including gray literature or unpublished studies, the representation of data from countries with limited research and publication capacity may be reduced. The study's focus on qualitative data limits the ability to quantify the burden of these challenges in TSCI prevention and care.

Kamoga Dickson, Morgan Broccoli

RE
ECLRS

Shakir M, Irshad HA, Ibrahim NUH, Alidina Z, Ahmed M, Pirzada S, et al. Temporal delays in the management of traumatic brain injury: A comparative meta-analysis of global literature. World Neurosurg. 2024; 188:185-98.

This global meta-analysis assessing time to intervention after a TBI found significant delays in care across prehospital and intrahospital settings, with disparities related to country-level income, region, and healthcare payment system.

Summary: In light of the critical impact of time to intervention after TBI on patient morbidity and mortality, this large meta-analysis examined time delays in TBI care across countries. The authors conducted a comprehensive literature review that included 45 studies, encompassing 94,991 patients from 45 countries. Countries were categorized by income level (low- or middle-income countries [LMIC] or high-income countries [HIC]), by WHO region, and by healthcare payment model (Single Payer Healthcare System [SPHS] or Multi-payer Healthcare System [MPHS]). Using random-effects modeling and subgroup analysis, the study found longer prehospital, intrahospital and total delays in LMICs compared to HICs, and in the African Region relative to other WHO regions. While MPHS systems experienced greater prehospital delays, SPHS systems showed greater intrahospital delays. The authors emphasize the urgent need to address global disparities in acute neurotrauma care and propose this study as a benchmark for evaluating future interventions.

Comment: This article provides a methodologically sound and timely synthesis of global disparities in TBI care. Its major strengths include a large sample size, diverse geographical representation, and detailed stratification analysis. However, high heterogeneity limits the statistical significance and generalizability of the findings. Furthermore, this study only included articles written in English, did not include gray literature, and lacked correlation with clinical outcomes such as mortality. This article does discuss potential causes for greater delays in LMICs and in Africa, which is crucial in contextualizing the findings. Further investigation is needed to elucidate the root causes of delays to design effective context-specific interventions. This study offers quantifiable evidence of global inequities in TBI care and highlights the need for health system improvements in neurotrauma.

Cheyenne Smith, Amanda Collier

OR
ECLRS

Sri-on J, Phumsrisawat J, Rojsaengroeng R. Missed opportunity to diagnose palliative care need among older emergency department patients in a middle-income country: A retrospective study. Open Access Emerg Med. 2024; 16: 65-73.

This study from Thailand identifies and characterizes significant missed opportunities to assess the need for palliative care in the emergency department for patients suffering with life-limiting illness. The provision of palliative care has the potential to improve overall quality of life, decrease pain, and decrease medical costs, particularly during end-of-life emergency care.

Summary: This retrospective descriptive study was conducted in an urban government teaching hospital in Bangkok, Thailand with a primary objective of identifying and characterizing missed opportunities for practicing palliative care in the emergency department (ED). Over a three-year period, 185 patients aged sixty-five and older with chronic medical illness were enrolled and divided into two groups: those who died in the emergency department or within seventy-two hours of disposition. In chart review, it was determined that 87% of patients would have benefited from palliative care, with 37% of cases identified as missed opportunities. Patients were more likely to have a missed opportunity if diagnosed with ischemic heart disease than if they had known cancer. Rates of intubation and CPR were higher in the missed opportunity groups. The study authors suggest that palliative care planning leading up to and during end-of-life ED presentations would benefit patients by reducing pain, improving quality of life and decreasing the use of invasive procedures, in turn decreasing the cost of medical care.

Comment: Palliative care in the ED has been identified as an important, but under-utilized treatment pathway. This study contributes to the literature by characterizing missed opportunities for introducing palliative care in the ED setting and demonstrating a cost benefit for its use. As a retrospective chart review study, there are limitations in identifying palliative care discussions that may not have been documented in the medical records. The study authors further recognize that given the cultural complexities of palliative care provision, their findings may not be generalizable to the greater Thai or global population.

Cassandra Clay, Jennifer Jones

OR
ECLRS

Stephen S, Mohanty CR, Radhakrishnan RV, Issac A, Jacob J, Krishnan N, et al. Clinico-epidemiological profile, trends, and health-related outcomes of snakebite victims: A one-Year prospective study from Eastern India. *Wilderness Environ Med.* 2024;35:155-65.

A prospective observational study of individuals presenting with snakebites to a tertiary hospital in Eastern India summarized factors associated with these bites and clinical outcomes. Based on these results, the team highlights the need for programs to improve public knowledge of first aid measures to care for snakebites and the importance of seeking timely medical care for consideration of potentially life-saving antivenom.

Summary: This prospective observational study sought to characterize the socio-epidemiological factors associated with snakebites in Bhubaneswar, India. The research team identified 145 individuals presenting to a tertiary care hospital over a one-year study period, and collected information related to their demographics, the circumstances surrounding the bite, prehospital barriers to care, treatments and associated adverse reactions, and complications associated with the envenomation. Their results demonstrated that snakebites in this region commonly occurred during the early monsoon period (June-August) and primarily affected men, younger individuals (aged 17-45), farmers, and those residing in rural areas. They also examined the patients' knowledge regarding first aid care for snakebites and further identified transportation issues as a major barrier to timely care. They characterized the local effects and systemic complications of the bites across the four species of snake included in the analysis. Finally, they noted excellent efficacy of the equine polyvalent anti-snake venom, with a low overall mortality rate (2.1%) for those treated with antivenom. The authors call for social programs to educate communities on first-aid measures for snakebites and the importance of seeking timely care from medical experts.

Comment: The prospective observational format of this study was an appropriate choice to collect a large breadth of data on demographics, patient factors, and outcomes; this minimizes the risk of response bias. Several values within the results section are compared to those of similar studies by other groups and are found to corroborate the results of these studies. There is some limitation in the generalizability of the results to other groups, given that the study only included patients cared for within a single tertiary center. This may underestimate the true morbidity and mortality of these bites, by excluding those cared for at centers without antivenom, or those unable to present to a medical facility. Further, there was no post-hospitalization follow-up, which may underestimate the long-term morbidity and mortality of these injuries. Overall, this is a quality study that helps to characterize an important topic related to the study population. The authors' proposed initiatives to educate local populations are a crucial next step to minimize the consequences of snakebites in the region and beyond.

Brandon Friedman, Vinay N. Kampalath

OR
ECLRS

Tanaanantarak P, Suntornsawat S, and Samphao S. Clinical characteristics associated with pediatric traumatic intracranial hemorrhage. Chin J Traumatol. 2024. 27: 334-9.

Clinical predictors including injury mechanism and severity, reduced GCS, signs of skull fracture on exam, and vomiting ≥3 times were significantly associated with intracranial hemorrhage on CT in pediatric head trauma patients in Thailand.

Summary: This retrospective cross-sectional study conducted in a level one trauma center in southern Thailand investigated clinical predictors of intracranial hemorrhage (ICH) in 475 children aged less than15 years of age with traumatic brain injury (TBI) . All patients underwent CT within 24 hours of injury. An ICH was detected in 98 (20.6%) patients, with six clinical factors significantly associated with ICH on multivariable analysis: blunt high-energy injury, motor vehicle accident, Glascow Coma Scale (GCS) less than 13, palpable skull fracture, signs of basilar skull fracture, or vomiting ≥3 times. In those falling in the mild head injury group (GCS ≥13), ICH was found in 16% and associated with these same clinical predictors. Among 203 patients in the observation/shared decision-making (SDM) group per the previously developed Pediatric Emergency Care Applied Research Network (PECARN) guidelines, 32 (15.7%) had ICH, most of whom exhibited multiple clinical risk factors. One child with mild head injury classified as SDM per PECARN required neurosurgical intervention.

Comment: These findings suggest that clinical criteria can reliably guide the need for CT imaging in children with traumatic head injury in settings outside the US with high rates of road traffic accidents. In children with signs of mild injury (GCS>13), additional consideration of need for CT should be taken if the child has multiple high-risk clinical signs and symptoms. Limitations of this study include the use of retrospective data from a single trauma center with timely access to neurosurgery, limited cases of severe injury, and lack of inclusion of longer-term outcomes or data on missed cases of children who did not initially undergo CT after injury.

Anneka Hooft, Chris Rees

RE
ECLRS

Wang L, Norman I, Edleston V, Oyo C, Leamy M. The effectiveness and implementation of psychological first aid as a therapeutic intervention after trauma: An integrative review. Trauma Violence Abuse. 2024 Oct;25(4):2638-56.

Psychological First Aid (PFA) has been shown to have a positive effect on anxiety and adaptive functioning following traumatic exposure, but significant variability in format and implementation complicates the development of best practices for PFA.

Summary: Psychological First Aid (PFA) is a widely used initial psychosocial approach to helping trauma-affected populations with roots dating back to World War II. PFA has since been adopted and adapted to many different contexts, leading to significant heterogeneity in format, timing, and duration. In this review article, the authors sought to compare and evaluate PFA models to determine their effectiveness and alignment with Hobfoll's five intervention elements. In addition, they sought to compare the variation in implementation of the different models, as well as, how they were experienced by both PFA providers and recipients. Their analysis found that PFA interventions most positively affect anxiety and adaptive functioning after trauma, but the effect on depression and post-traumatic stress disorder (PTSD) is less pronounced. In addition, most PFA approaches addressed four of the five Hobfall elements—safety, calm, efficacy, and connectedness—although the fifth element, hope, was not as widely or strongly incorporated. Components common to many PFA models include active listening, stabilization, practical assistance, and social connection. Furthermore, while both those receiving and delivering PFA find it helpful, those delivering PFA raised concerns about inadequate training, secondary traumatic stress, and the balance between tailoring an approach to best serve a specific context and maintaining fidelity to the approach. Overall, the degree of heterogeneity makes it difficult to inform an overall set of best practices.

Comment: With increasing attention regarding the use of PFA in trauma-affected populations, the authors raise the important question of what truly constitutes PFA. While the adaptability of PFA to different contexts is a strength, it has also contributed to the development of many different published approaches, each with their own format, duration, and timing, ranging from single interventions in the immediate aftermath of a traumatic event to multiple sessions up to two years later. This variability, combined with practical and other implementation concerns, impedes training and efforts of frontline providers to deliver PFA. Furthermore, it significantly complicates efforts to determine a set of best practices. However, despite these considerable differences in format, it is encouraging that the evidence demonstrates a positive effect on anxiety, adaptive functioning (including quality of life and coping skills), resilience, and self-efficacy, even if the effect is not as strong on PTSD and depression, especially in the long-term.

Agatha Brzezinski, Ashley Jacobson

OR
ECLRS

Yang J, Zhao Y, Wang J, Ma L, Xu H, Leng W, Wang Y, et al. Current status of emergency medical service use in ST-segment elevation myocardial infarction in China: Findings from China Acute Myocardial Infarction (CAMI) registry. *Int J Cardiol*. 2024; 406:132040.

This study examines emergency medical service (EMS) utilization in Chinese patients with ST-segment elevation myocardial infarction (STEMI). It analyzes patient characteristics, response times, and survival outcomes to assess the effectiveness of pre-hospital care strategies.

Summary: The article evaluates the impact of EMS utilization on STEMI patient outcomes in China, focusing on key metrics such as symptom-to-door time, EMS arrival time, and in-hospital mortality. Using a retrospective cohort analysis of national health data that included 26,305 STEMI patients, 4,336 were transported by EMS. EMS transport was linked to faster symptom onset-to-door times and increased reperfusion rates, but EMS transport did not significantly reduce treatment delays or mortality. The study highlights disparities in EMS use across different regions and the influence of socioeconomic factors. Findings suggest that EMS utilization significantly improves treatment timelines, yet adoption remains suboptimal due to system inefficiencies and public awareness gaps. Recommendations emphasize enhanced public education and policy reforms to optimize emergency cardiac care accessibility.

Comment: The study provides valuable insights into EMS effectiveness for STEMI care, revealing both systemic strengths and weaknesses. While this cohort demonstrated the valuable role of EMS in reducing STEMI patient onset-to-door times and higher rates of reperfusion, it also highlighted barriers to utilization, particularly in rural areas. The authors propose regionalized coordinated EMS for STEMI care as a potential solution to further improve outcomes. The article contributes to global discussions on pre-hospital cardiac care and calls for structural improvements in emergency response protocols.

Abdirahman Abdulle, Nanaba Dawson-Amoah

OR
EMD

Behnoush AH, Alizadeh N, Emami M, Bazmi E, Alimohamadi Y, Behnoush B. Effects of intravenous lipid emulsion administration in acute tramadol poisoning: A randomized controlled trial. J Emerg Med. 2024; 66:154-62.

This randomized controlled trial assessed the use of intravenous lipid emulsion (ILE) therapy among patients with acute tramadol poisoning and a GCS of less than 12. It found that those who underwent ILE administration had significantly reduced seizure frequency, length of hospitalization, and higher GCS when compared to controls receiving treatment with saline.

Summary: In this double-blinded randomized control trial conducted in Tehran, Iran, researchers assessed the efficacy of intravenous lipid emulsion (ILE) therapy in managing acute tramadol poisoning. The study enrolled 120 patients aged 16 to 50 years, all presenting with a pure tramadol ingestion of greater than 400mg, a positive urine test for tramadol and no other substances, and a Glasgow Coma Scale (GCS) score of 12 or lower. Participants were randomly assigned to receive either standard supportive care and a control infusion, which was 0.9% saline or standard supportive care supplemented with 20% ILE. The primary outcomes measured included seizure occurrence, GCS, and length of hospital stay. Findings revealed that the ILE group experienced a significantly lower incidence of seizures (26.7%) compared to the control group (50%). Patients receiving ILE demonstrated improved consciousness levels, indicated by a higher median GCS score post-treatment (12 [IQR 10–13] vs. 10 [IQR 8–12]), and had shorter hospital stays (median of two days [IQR 1–3] versus four days [IQR 4–6]). These findings suggest that ILE may be beneficial for acute tramadol poisoning, although further research is recommended to assess these findings in a larger sample size.

Comment: This study supports the use of intravenous lipid emulsion as a treatment for acute tramadol poisoning in patients with decreased GCS scores. A strength of this study is its design as a double-blinded randomized control trial. A weakness is the absence of long-term follow-up data, which raises questions regarding the sustainability of clinical improvements and any potential delayed adverse effects or impact on longer term clinical outcomes. The sample size is both a possible strength and weakness, as patients presenting with an isolated tramadol overdose are uncommon, although a larger study would strengthen the power of the findings. This study is particularly relevant in regions where tramadol overdose is common. Given the study was limited to patients with pure tramadol ingestion, the results may not be generalizable to mixed ingestions. Additionally, many locations may not have access to ILE, decreasing the feasibility of implementing this treatment in low-resource settings. Overall, this trial contributes important data to a relatively limited body of literature on lipid therapy in non-local anesthetic toxicity and provides a foundation for future studies to expand its potential indications.

Sarah Rapaport, Joseph Leanza

OR
EMD

Koko JAB, Mohamed OSA, Koko BAB, Musa OAY. The ABCDE approach: Evaluation of adherence in a low-income country. Injury. 2024. 55:111268.

The authors researched the use of the ABCDE approach while managing trauma patients in three of the major trauma centers in Khartoum, Sudan. The study showed only 37.9% adherence to the approach, suggesting a need for improved education and implementation of trauma management guidelines.

Summary: The assessment of Airway, Breathing, Circulation, Disability and Exposure (ABCDE) approach helps quickly identify and treat life threatening injuries in trauma patients. This cross-sectional study, done in three major trauma centers in Khartoum, Sudan used an observation checklist to assess the adherence of emergency care teams to the ABCDE approach when managing trauma patients. It showed only 37.9% adherence to the approach in this setting. Overcrowding and poor facility preparation were identified as major obstacles to following the approach, while clinical experience and adequate training were potential facilitators.

Comment: The study showed that although the ABCDE approach was often rapidly started after patient arrival, it was rarely followed to completion. The main medical staff responsible for trauma care in this setting were general practitioners or medical officers, which may limit generalizability to other settings with different providers available. The authors note limitations of a short study period and low enrollment (50). They also note that an item was marked done if it was started, even if it was not done completely (and it is unknown if steps were done correctly).This might overestimate the already low completion rate. This study suggests a need for more research from other low-income countries to further identify the barriers to using the ABCDE approach. This would inform training programs and QI projects to improve adherence, and therefore might improve trauma patient management. Standardized guidelines should be emphasized in training for care teams managing trauma patients.

Fadhila Tekka, Amanda Collier

OR
EMD

Michalski K, Diedhiou M, Grushka J, Tendeng J, Diao M, Beye M, et al. Advancing access to care: An assessment of the prehospital system in Senegal. World J Surg. 2024:1056-65

The Senegalese experience in prehospital care highlights the urgent need for restructuring of nascent prehospital systems in low-resource settings for efficient and quality emergency medical services.

Summary: This study reported on the critical need to strengthen underdeveloped prehospital care infrastructure serving regional health systems in Senegal. A mixed-methods study was conducted to appraise the prehospital care quality and coverage across 10 regions of Senegal. A purposeful sampling of relevant key stakeholders and emergency medical service providers was done using a locally developed, adapted and tailored questionnaire alongside key informant interviews or phone surveys. Findings showed that 80% of the included regions did not have an established prehospital system in place. Emergency medical responses were provided most often by firefighters who did not have the requisite knowledge and skillsets for providing basic emergency care and first aid on the scene or in transit. The authors advocated for key players, including government agencies, to strengthen prehospital systems through formal emergency medical service training, coordination, and restructuring.

Comment: This study presented novel findings on prehospital care and informed a course of action for improving prehospital care in Senegal. The findings are consistent with contemporary reports in counterpart African nations with similar challenges. Hence, the findings drive changes in practice and inform decision-making by relevant stakeholders in Senegal and, by extension, neighboring developing countries. Nevertheless, this was a single-country study which limits its full generalizability. The authors attempted to limit bias in their findings by including a diverse population of respondents and by double checking participant responses to ensure what was reported reflected what was intended. This study is relevant to global emergency medicine as it calls for emergency medicine development in prehospital care that is particularly relevant to the African context.

Idowu Emmanuel Oluyinka, Chris Rees

OR
ECLRS

Nasr Isfahani M, Etesami H, Ahmadi O, Masoumi B. Comparing the efficacy of intravenous morphine versus ibuprofen or the combination of ibuprofen and acetaminophen in patients with closed limb fractures: A randomized clinical trial. BMC Emerg Med. 2024;24:15.

This study assessed pain management modalities in isolated closed extremity fractures in adults. Superior pain control at 60 minutes was found in the IV ibuprofen-acetaminophen group as compared with IV morphine or IV ibuprofen alone.

Summary: The majority of fractures sustained by patients are closed extremity fractures, which often cause significant pain. Multiple studies have explored multimodal approaches to pain control for patients with fractures. This study evaluated analgesia effectiveness in three groups: intravenous (IV) ibuprofen alone; IV ibuprofen in combination with IV acetaminophen; and IV morphine alone. This triple-blinded, randomized controlled trial was conducted at a trauma center in Iran. All participants received standard of care non-pharmacologic therapies (including ice, compression, and elevation therapy), were triaged as Emergency Severity Index level two or three, and received medication dosing in accordance with prior studies. Pain levels were assessed using the Visual Analog Scale (VAS) at multiple time points over the course of an hour after medication administration. Most participants presented with upper extremity fractures. At the first time point, five minutes after administration, all three groups showed a significant reduction in VAS scores, with a greater initial decrease observed in the ibuprofen and ibuprofen-acetaminophen groups. Although morphine yielded the most substantial pain reduction at 15 minutes, its effect plateaued, and morphine's analgesia effects were eventually surpassed by the ibuprofen-containing regimens. At one-hour, the ibuprofen-acetaminophen combination group reported the lowest overall pain scores. All changes in pain levels were statistically significant across time points, except at the 30-minute mark, where no significant difference was found between morphine and the ibuprofen-acetaminophen combination.

Commentary: This study was conducted over a relatively short period, among individuals aged 15 to 60 presenting with isolated extremity fractures. The limited study period may affect the generalizability of findings across different seasons or trauma patterns. A key limitation of the study is the short follow-up period, with pain outcomes measured only up to one hour after medication administration. The need for redosing or longer-term analgesic efficacy was not captured. The timing of peak analgesic effect varies by medication type, and a longer observation window could have better assessed the duration and sustainability of pain relief. An additional concern is the availability of IV ibuprofen and acetaminophen as compared to their enteral counterparts, which may limit the generalizability of this study. Additionally, the study notes variability in the type of closed extremity fractures. Finally, while the VAS is a commonly used tool for pain assessment, it is subject to potential bias influenced by patient interaction, facial expression, and observer interpretation; the authors did attempt to mitigate this by analyzing changes in pain score over time rather than absolute values.

Juliette Gerardo, Joseph Leanza
